# Screening archaeological bone for palaeogenetic and palaeoproteomic studies

**DOI:** 10.1371/journal.pone.0235146

**Published:** 2020-06-25

**Authors:** Ioannis Kontopoulos, Kirsty Penkman, Victoria E. Mullin, Laura Winkelbach, Martina Unterländer, Amelie Scheu, Susanne Kreutzer, Henrik B. Hansen, Ashot Margaryan, Matthew D. Teasdale, Birgit Gehlen, Martin Street, Niels Lynnerup, Ioannis Liritzis, Adamantios Sampson, Christina Papageorgopoulou, Morten E. Allentoft, Joachim Burger, Daniel G. Bradley, Matthew J. Collins

**Affiliations:** 1 Department of Archaeology, BioArCh, University of York, York, United Kingdom; 2 Department of Chemistry, BioArCh, University of York, York, United Kingdom; 3 Smurfit Institute of Genetics, Trinity College Dublin, Dublin, Ireland; 4 Department of Earth Sciences, Natural History Museum, London, United Kingdom; 5 Palaeogenetics Group, Institute of Organismic and Molecular Evolution (iomE), Johannes Gutenberg-University Mainz, Mainz, Germany; 6 Laboratory of Physical Anthropology, Department of History and Ethnology, Democritus University of Thrace, Komotini, Greece; 7 German Federal Criminal Police Office, Wiesbaden, Germany; 8 Centre for GeoGenetics, GLOBE Institute, University of Copenhagen, Copenhagen, Denmark; 9 Department of Archaeology, McDonald Institute for Archaeological Research, University of Cambridge, Cambridge, United Kingdom; 10 Collaborative Research Centre, project D4, Cologne University, Cologne, Germany; 11 MONREPOS Archaeological Research Centre and Museum for Human Behavioural Evolution, RGZM Leibniz Research Institute for Archaeology, Neuwied, Germany; 12 Unit of Forensic Anthropology, Department of Forensic Medicine, University of Copenhagen, Copenhagen, Denmark; 13 Laboratory of Archaeometry, Department of Mediterranean Studies, University of the Aegean, Rhodes, Greece; 14 Center on Yellow River Civilization of Henan Province, Key Research Institute of Yellow River Civilization and Sustainable Development and Collaborative Innovation, Henan University, Kaifeng, China; 15 Department of Mediterranean Studies, University of the Aegean, Rhodes, Greece; 16 Centre for Evogenomics, Globe Institute, University of Copenhagen, Copenhagen, Denmark; Max Planck Institute for the Science of Human History, GERMANY

## Abstract

The recovery and analysis of ancient DNA and protein from archaeological bone is time-consuming and expensive to carry out, while it involves the partial or complete destruction of valuable or rare specimens. The fields of palaeogenetic and palaeoproteomic research would benefit greatly from techniques that can assess the molecular quality prior to sampling. To be relevant, such screening methods should be effective, minimally-destructive, and rapid. This study reports results based on spectroscopic (Fourier-transform infrared spectroscopy in attenuated total reflectance [FTIR-ATR]; n = 266), palaeoproteomic (collagen content; n = 226), and palaeogenetic (endogenous DNA content; n = 88) techniques. We establish thresholds for three different FTIR indices, a) the infrared splitting factor [IRSF] that assesses relative changes in bioapatite crystals’ size and homogeneity; b) the carbonate-to-phosphate [C/P] ratio as a relative measure of carbonate content in bioapatite crystals; and c) the amide-to-phosphate ratio [Am/P] for assessing the relative organic content preserved in bone. These thresholds are both extremely reliable and easy to apply for the successful and rapid distinction between well- and poorly-preserved specimens. This is a milestone for choosing appropriate samples prior to genomic and collagen analyses, with important implications for biomolecular archaeology and palaeontology.

## 1. Introduction

Ancient DNA (aDNA) extracted from archaeological and palaeontological bone (palaeogenetics) can provide insights on a plethora of different topics related to human evolution [1,2] and past societies (mobility [3,4]; biological sex [5,6]; kinship [7,8]; pathology [9,10]; animal domestication [11,12]). However, DNA is a relatively unstable biomolecule which undergoes progressive fragmentation post-mortem [13–18]. DNA degradation is caused by a wide range of biological, chemical, and environmental factors in the burial environment [16,19,20], and the post-excavation treatment and storage of archaeological bone may further damage the DNA [15,21–24].

The preservation of ancient proteins (palaeoproteomics) is of interest to archaeologists, anthropologists, and archaeological scientists as they can provide valuable information on the chronological age (^14^C dating), amino acid racemization [25,26], past dietary habits [27,28], and animal species identification [29–31]. Although proteins are more stable than DNA, and can therefore survive much longer (e.g. [32] and references therein), the dominant protein in bone (collagen) also suffers post-mortem from biological, chemical, and environmental factors [33–35].

Despite the falling costs in palaeogenetic and palaeoproteomic studies, the establishment of any reliable screening method is a valuable tool. This is due to the technical difficulties associated with the recovery and analysis of aDNA and protein from bone, the partial destruction of valuable or rare specimens, and the still-high sequencing costs.

Previous studies have explored the association of endogenous DNA yields with several parameters including gross morphology and bone size [36,37], histology [36,38–40], collagen preservation [20,41,42], or amino acid racemization [43–45]. The efficiency of the macroscopic and/or microscopic appearance of bone as tools for screening have been disputed due to their subjective nature (inter-observer error), and the lack of accuracy and precision which result in unreliable screening practices. Aspartic acid racemization has been challenged [43] on the basis that in bone (and open-system) racemization records the proportional loss of (highly racemized) gelatin, not the state of DNA preservation, while the relationship of collagen content to endogenous DNA yield requires further investigation.

The efficiency of the mid-IR spectra for screening archaeological bone has also been previously investigated, as it can potentially provide valuable information for predicting the endogenous DNA content. As DNA molecules have been found to be protected from further degradation by adsorbing onto the bioapatite (BAp) crystal surfaces under favourable environmental conditions [16,18,19,41,46], any structural and compositional changes observed in BAp crystals using the semi-quantitative infrared splitting factor [IRSF; 47] and carbonate-to-phosphate ratio [C/P; 48] should be linked to endogenous DNA survival. The IRSF reflects the relative changes in average BAp crystal size and homogeneity, while the carbonate-to-phosphate ratio reflects the relative changes in the carbonate environment of BAp (i.e. carbonate loss, carbonate uptake via adsorption to crystal surfaces or substitution in the crystal lattice, and phosphate-carbonate exchange [49]). However, although FTIR spectroscopy is often proposed as a potential screening tool for endogenous DNA [39,40,45,50] in archaeological bone due to its minimally-destructive, time- and cost-efficient nature, the prediction of the endogenous DNA survival has been deemed extremely difficult and it has never been linked to the amount of endogenous DNA preserved in bone [39,45].

Efforts have also been made to screen archaeological bone for collagen preservation, with the use of the whole bone nitrogen [51–53] and carbon content [40] currently being the most promising predictors of collagen survival, although these are destructive. FTIR spectroscopy also has the potential to constitute a reliable screening tool for archaeological bone collagen preservation. However, post-mortem changes in BAp crystals, which can expose collagen fibrils and accelerate to some extent collagen degradation (i.e. fragmentation of the polypeptide chains [35,54–57] reflected in changes in IRSF and C/P), often show no strong correlation with collagen wt. % [49]. The Am/P ratio (amide-to-phosphate; 1640 cm^-1^/ 1010 cm^-1^) has been extensively used for the assessment of the organic content in bone [58,59], as the amide I peak at c. 1640 cm^-1^ is considered characteristic of proteins and peptides [60,61]. Nevertheless, issues related to the presence of overtones due to the chemical treatment of bone, the O-H stretch vibration at 1640–1660 cm^-1^ related with structural water, or the presence of humic acids, can often lead to inaccurate reporting of collagen preservation [59,62].

This study proposes FTIR-ATR (Fourier transform infrared spectroscopy in attenuated total reflectance) as a reliable, rapid screening tool for endogenous DNA and collagen preservation in archaeological bone. As archaeological specimens are finite and palaeogenetic and palaeoproteomic research projects have limited financial resources, thresholding specimens for endogenous DNA and collagen preservation prior to sampling could be advantageous for specimen and/or methodology choice. Here we follow a different approach as we focus on identifying samples with > 1% and > 10% endogenous DNA; these are plausible minimum thresholds for researchers interested in the generation of nuclear ancient genomes via Next Generation Sequencing technology and employing different levels of analysis. Similarly, two different collagen yield (wt.%) thresholds have been selected (i.e. 3% [63] and 2% [60]); these have been widely used for the distinction between well- and poorly-preserved collagen samples in palaeodietary, radiocarbon, and palaeoproteomic studies and are therefore applied in this study for collagen screening.

The minimally destructive nature of FTIR-ATR means that it is a breakthrough in choosing appropriate samples prior to palaeogenetic and palaeoproteomic analyses. This method has important implications for biomolecular archaeology and palaeontology as it enables the distinction between well- and poorly-preserved specimens. By linking the % endogenous DNA and collagen wt. % to defined thresholds for IRSF, C/P and/or Am/P, this standardised approach is very reliable and easy to apply.

## 2. Materials and methods

### 2.1. Materials

Samples came from 20 archaeological sites (see S1 and S2 Tables), of which one was from Germany (c. 10,000 B.C.), one from Jordan (7500–5500 B.C.), six from Greece (8300–800 B.C.), one from Central Asia (2100–1800 B.C.), nine from Britain (3200 BC-1100 A.D.), one from Belgium (900–1800 A.D.), and one from Denmark (1650–1850 A.D.). The aim was to study both human and animal skeletal remains of different chronological ages that originate from different geographic locations and burial environments. Petrous bones are representative of the b and/or c areas as presented in Pinhasi et al. [64]. All necessary permits, which complied with all relevant regulations, were obtained from the authorities, institutions and/or researchers mentioned in the acknowledgement section.

### 2.2. FTIR-ATR

The preparation and analysis of 266 samples (including 102 petrous bones; S1 Table), was carried out following Kontopoulos et al. [65]. This methodological approach was selected as it significantly improves accuracy, precision, reproducibility and comparability of data, as seen in a subset of fifteen archaeological, one modern bovine and one synthetic hydroxyapatite (HAp) samples which were run in triplicate in a Bruker Alpha Platinum and a Bruker Vertex 70v in vacuum using the same parameters (see S3 Table).

Bone samples were ground using an agate pestle and mortar following the mechanical cleaning of the outer and inner bone surfaces. Pulverization of the samples from British archaeological sites was carried out using a Retsch oscillating steel ball mill grinder at 20 Hz for 20–25 seconds x 3–5 times. Samples were run in triplicate and about 2–3 mg of bone powder of 20–50 μm particle size were used for each measurement using a Bruker Alpha Platinum. The crystal plate and the anvil of the pressure applicator were thoroughly cleaned using isopropyl alcohol after each measurement. The mid-IR spectra were analysed using OPUS 7.5 software and FTIR indices were calculated after baseline correction, as reported in Kontopoulos et al. [65]. The infrared splitting factor (IRSF = 600 cm^-1^ + 560 cm^-1^ / 590 cm^-1^) was assessed after Weiner and Bar-Yosef [47], the carbonate-to-phosphate ratio (C/P = 1410 cm^-1^ / 1010 cm^-1^) was calculated as in Wright and Schwarcz [48], and the amide-to-phosphate ratio (Am/P = 1640 cm^-1^ / 1010 cm^-1^) was assessed according to Trueman et al. [58]. The Am/C_1_ (1640 cm^-1^ / 1410 cm^-1^) and Am/C_2_ (1640 cm^-1^ / 872 cm^-1^) ratios used two different CO_3_^2ˉ^ bands. The 872 cm^-1^ peak height was calculated after baseline correction drawn from c. 830 to c. 890 cm^-1^.

### 2.3. Collagen analysis

Collagen was extracted from 226 samples (including 62 petrous bones; see S1 Table) using a modified Longin [66] method as in Kontopoulos et al. [49]. The exterior surfaces of bone samples were mechanically cleaned using a scalpel. Bone chunks of 300–500 mg were demineralized in 8 mL 0.6 M HCl at 4° C. Samples were agitated twice daily, and acid solution was changed every two days. When demineralization was completed, the supernatant was drained off and samples were rinsed three times with distilled water. Gelatinization was carried out by adding 8 mL pH 3 HCl and samples were placed in hot blocks at 80°C for 48 h. The supernatant liquor which contains the collagen was filtered off by using Eezee filters and was freeze-dried for 2 days in pre-weighed sterile plastic tubes. Collagen yields (wt.%) were estimated using the following formula: ***[bone mass (mg) / collagen mass (mg)] × 100***, where bone mass is the weight of bone chunks after cleaning the exterior surfaces, and collagen mass is the extracted material that remains following demineralization, gelatinization, and filtering.

### 2.4. DNA analysis

The endogenous DNA content of 88 samples (including 80 petrous bones; S1 Table), was provided by three different research groups in Europe. Laboratory work was performed according to strict aDNA standards in dedicated clean laboratory facilities at Trinity College Dublin (Daniel Bradley’s group), Johannes Gutenberg-University Mainz (Joachim Burger’s group), and the University of Copenhagen (Morten Allentoft’s group). The exterior surfaces of bones were mechanically removed using a drill or by sandblasting, and UV-irradiated prior to pulverization. DNA extraction, library preparation, sequencing, bioinformatics and DNA authenticity were conducted as described in Verdugo et al. [12], Hofmanová et al. [3] with modifications, Botigué et al. [67], and Hansen et al. [68] for Trinity College Dublin, Johannes Gutenberg-University Mainz and University of Copenhagen, respectively (see **section 2**—S1 Text for laboratory protocols). Endogenous DNA percentages were also estimated using the same bioinformatics pipeline (see **section 2**—S1 Text for the pipeline) for all samples to eliminate the potential effects of the different bioinformatics protocols followed by the three different labs on the estimated yields.

## 3. Results and discussion

### 3.1. Endogenous DNA screening using the IRSF and C/P

Despite the weak correlation (R^2^ = 0.24) between aDNA and crystallinity (IRSF) and the very weak relationship (R^2^ = 0.13) with carbonate content (C/P), likely due to the complex interactions during diagenesis (e.g. local hydrology, pH, temperature), there appear to be thresholds of both IRSF and C/P that are related to endogenous DNA preservation. This dataset, therefore, demonstrates that IRSF and C/P mid-IR indices can be considered reliable predictors of endogenous DNA preservation.

**Fig 1:**
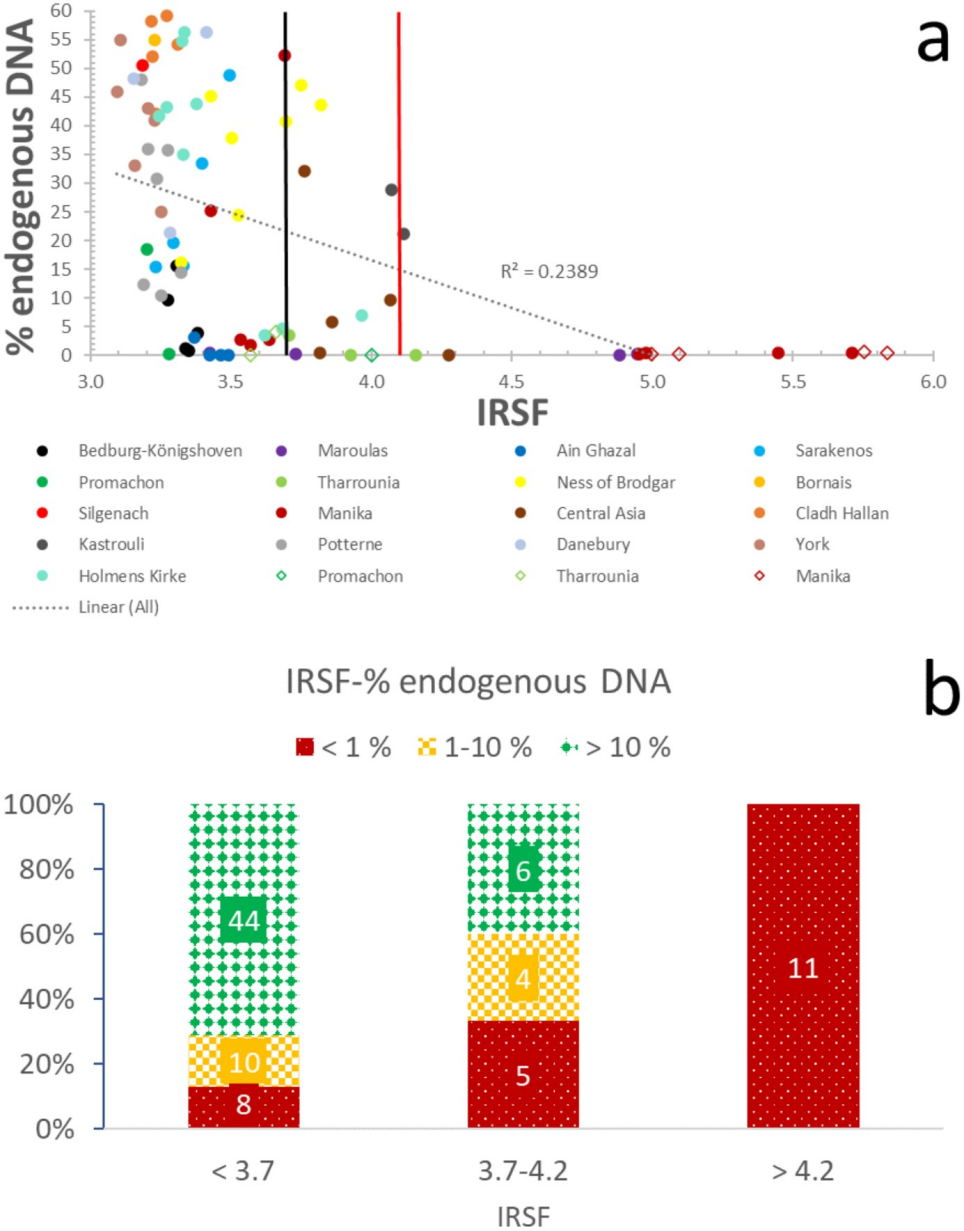
Endogenous DNA-crystallinity relationship. (a) Correlation of IRSF with endogenous DNA yields. The black line at 3.7 IRSF represents the proposed ‘strict’ threshold, while the red line at 4.2 IRSF represents the ‘moderate’ threshold. The circles represent the petrous bones, and the rhombuses the other skeletal elements. (b) Distribution of samples with well- (> 10%; green/solid diamond), moderately- (1–10%; yellow/large checkerboard), and poorly-preserved (< 1%; red/dotted) endogenous DNA in categories based on crystallinity.

The categorization of samples into three groups based on their endogenous DNA yields (i.e. > 10%, 1–10%, and < 1%) allows some very interesting observations to be made. In the case of well-preserved specimens with endogenous DNA > 10%, c. 90% of the samples (n = 44) display IRSF values ≤ 3.7, and only c. 10% (n = 6) have 3.7 ≤ IRSF ≤ 4.2 (Fig 1; S4 Table). When endogenous DNA yields drop between 1–10%, 71% of bones (n = 10) have an IRSF ≤ 3.7, whereas 29% (n = 4) have 3.7 ≤ IRSF ≤ 4.2 (Fig 1; S4 Table). Bones that yield endogenous DNA below 1% predominantly display IRSF values over 3.7 (c. 70%; n = 16), with a small subset (c. 30%; n = 8) characterized by crystallinity below the 3.7 threshold (Fig 1; S4 Table). Overall, c. 70% of all samples with IRSF ≤ 3.7 contain endogenous DNA > 10%, and a total of c. 90% contain endogenous DNA over 1%. All bones with IRSF > 4.2 yielded endogenous DNA below 1% (Fig 1; S4 Table). Furthermore, these results demonstrate that the effectiveness of the IRSF as a screening is not affected by fluctuations in endogenous DNA percentages due to differences in bioinformatic pipelines (S4 Table and S1 Fig).

Therefore, IRSF can successfully distinguish bones likely to be suitable for palaeogenetic analysis from those with poor endogenous DNA preservation, without any false negative samples (i.e. do not pass screening but have endogenous DNA content over 1%) and only a small number of false positive samples (i.e. 12 samples pass screening but have endogenous DNA content below 1%). For screening purposes, a small number of false positives is acceptable, while false negatives are less acceptable, so based on this dataset these IRSF parameters are likely to be useful. We thus suggest two different thresholds which allow aDNA labs to decide the optimal threshold depending on the characteristics and/or the number of their samples, the lab resources, etc. A ‘moderate’ cut-off value of 4.2 which identifies 100% of samples with endogenous DNA yields > 1% and eliminates about 40% of the samples with endogenous aDNA < 1%. A ‘strict’ cut-off value of 3.7 which identifies c. 90% of samples with endogenous DNA yields > 10%, c. 70% of bones with 1–10% endogenous DNA, while it eliminates c. 70% of samples with endogenous DNA < 1%.

**Fig 2:**
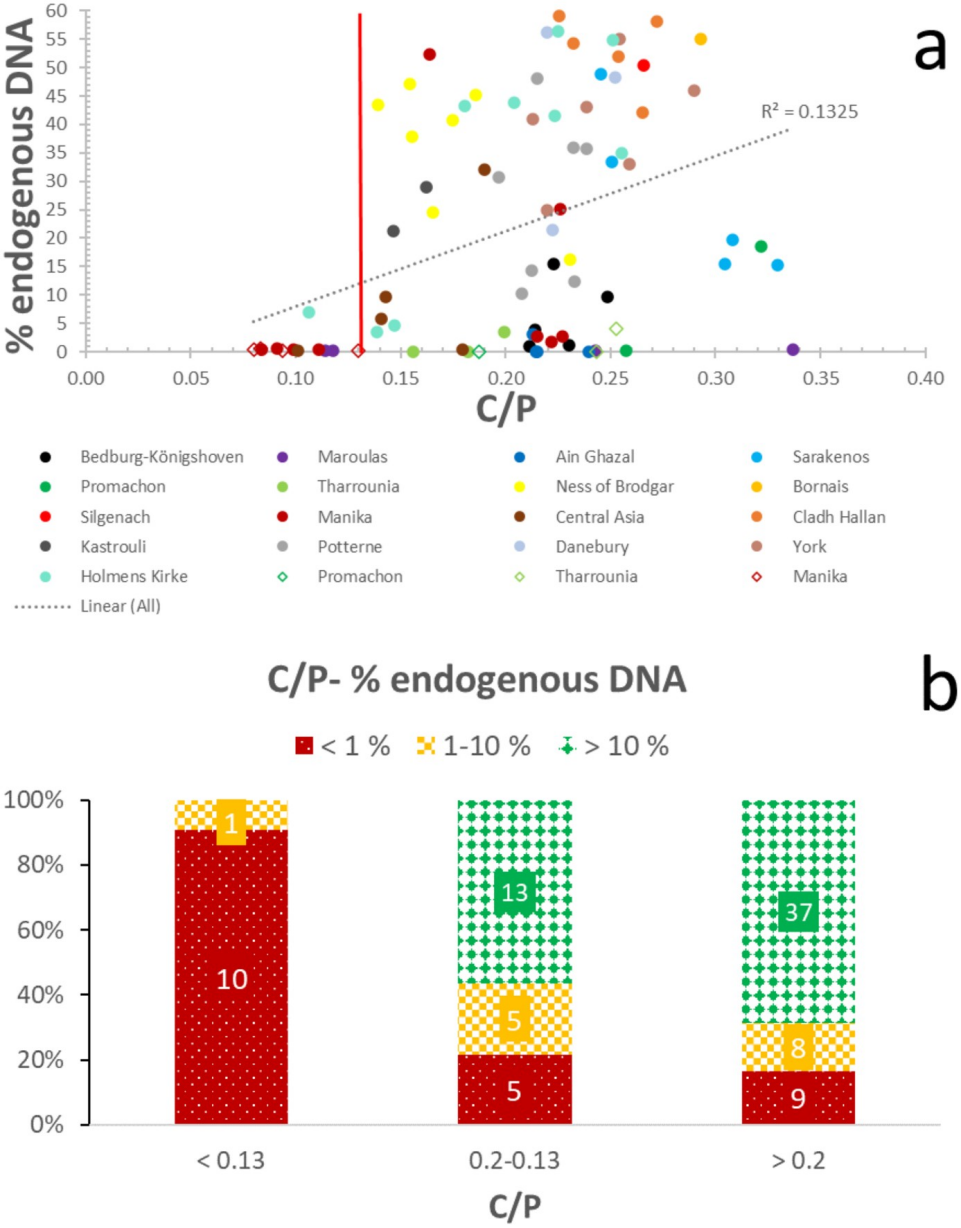
Endogenous DNA-carbonate content relationship. (a) Correlation of C/P with endogenous DNA yields. Line represents the proposed C/P = 0.13 cut-off point. The circles represent the petrous bones and rhombuses the other skeletal elements. (b) Distribution of samples with well- (>10%; green/solid dimond), moderately- (1–10%; yellow/large checkerboard), and poorly-preserved (< 1%; red/dotted) endogenous DNA in categories based on carbonate content.

In tandem, carbonate content can be used as a complementary endogenous DNA indicator, as c. 70% (n = 37) of the samples with C/P ratios > 0.20 contain endogenous DNA > 10%, while c. 85% (n = 45) display DNA yields over 1%, and only c. 15% (n = 10) yield endogenous DNA below 1% (Fig 2; S4 Table). When C/P decreases to the 0.13–0.20 levels, still 55% (n = 13) of bones yield more than 10% endogenous DNA, and c. 80% (n = 18) of bones yielding more than 1% endogenous DNA, while c. 20% (n = 5) of the samples have endogenous DNA yields below 1% (Fig 2; S4 Table). In levels below 0.13 C/P ratio, samples with endogenous DNA yields < 1% prevail (i.e. c. 90%; n = 10), and only c. 10% (n = 1) of samples contain more than 1% endogenous DNA (Fig 2; S4 Table). Thus, the success rate of C/P values ≥ 0.13 for the distinction of archaeological bone with endogenous DNA yields > 1% is almost 100%, and it eliminates c. 40% of poorly-preserved specimens. Compared to IRSF, there is only one false negative sample and the same false positive samples, while the processing of all samples using the same bioinformatic pipeline does not affect the success rates of this screening method (S4 Table and S2 Fig).

Consequently, FTIR-ATR can be an effective, minimally destructive, and rapid tool for DNA screening of archaeological and palaeontological bone with 100% successful identification of bones containing > 1% endogenous DNA and an elimination of about half of the samples with endogenous DNA yields below 1%.

### 3.2. Endogenous DNA screening using the collagen wt. %

**Fig 3:**
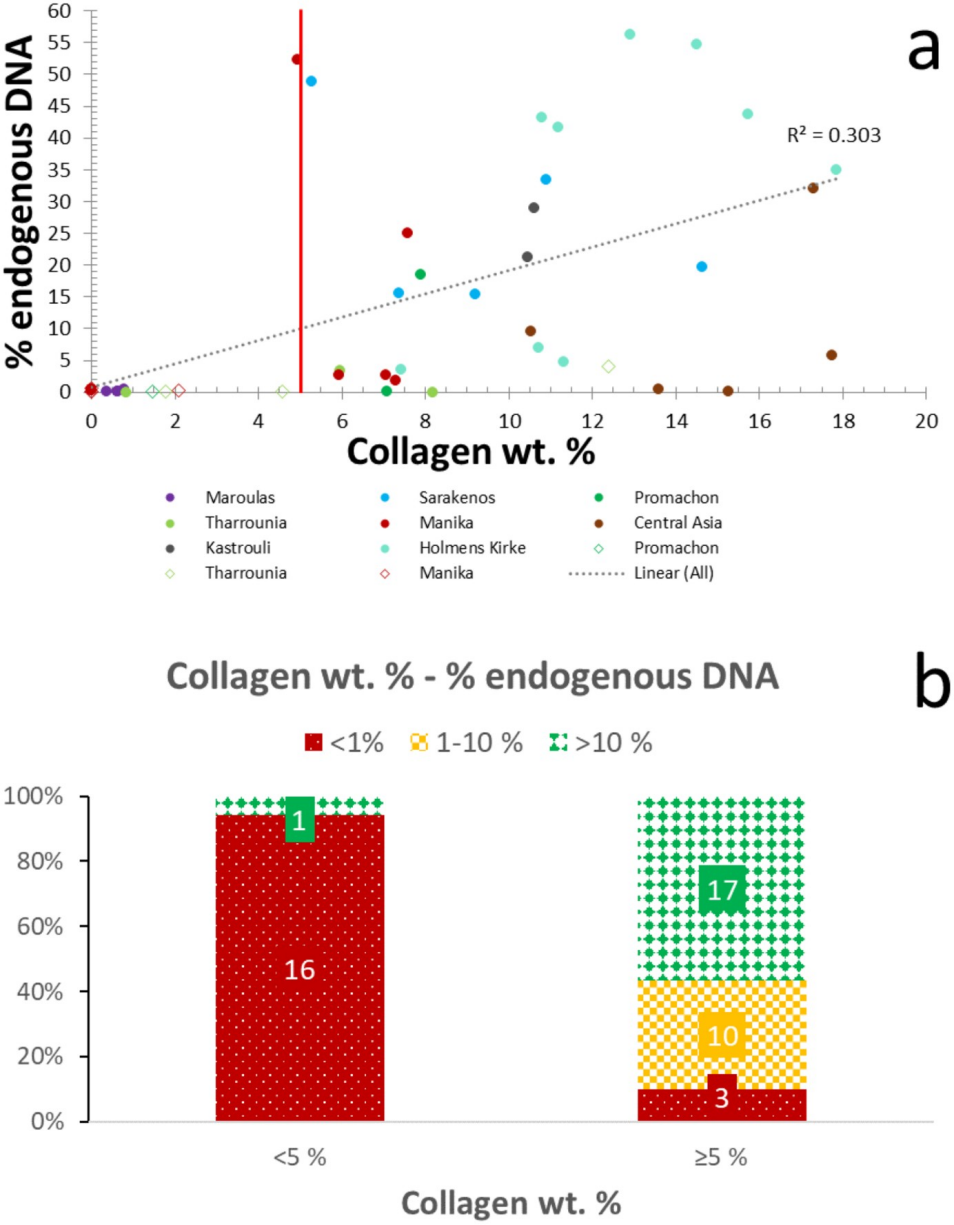
Endogenous DNA-collagen content relationship. (a) Endogenous DNA preservation shows poor correlation with collagen wt. %. Line represents the proposed collagen wt. % = 5 cut-off point. The circles represent the petrous bones, while the rhombuses represent the other skeletal elements. (b) Distribution of samples with well- (> 10%; green/solid diamond), moderately- (1–10%; yellow/large checkerboard), and poorly-preserved (< 1%; red/dotted) endogenous DNA in categories based on collagen yield.

A handful of studies have previously reported a link between collagen and DNA degradation [20,41,42]. DNA molecules have the ability to bind to the collagen fibrils and create a DNA-collagen complex [69,70]. Fig 3 demonstrates a weak correlation (R^2^ = 0.30) between collagen wt. % and endogenous DNA yields, as samples with relatively good collagen preservation may not yield endogenous DNA, and vice versa. Although a lack of correlation has also been reported in previous studies [e.g. 71], this dataset shows a possible cut-off point at c. 5% collagen by weight which can be potentially used as endogenous DNA predictor (Fig 3; S4 Table). Specifically, c. 95% (n = 12) of the samples with collagen wt. below 5% display endogenous DNA yields < 1%, and only c. 5% (n = 1) have high endogenous DNA preservation (i.e. > 10%) (Fig 3). Similarly, c. 90% of the samples containing over 1% endogenous DNA also have collagen wt. % over 5 (Fig 3). Therefore, when collagen content drops below 5%, the chances for aDNA survival seem to diminish.

However, even if DNA decay accelerates with increasing collagen hydrolysis, screening archaeological bone using collagen analysis is typically a destructive method, (but see the recent report of the successful use of near-infrared [72]) is also relatively time-consuming and labour-intensive, while the widespread use of an additional ultrafiltration step can have a strong influence on the collagen yields [73–75]. Therefore, while collagen yield seems to be an effective method to predict endogenous DNA content, it is most useful as an additional screening tool when samples have been already prepared for radiocarbon / stable isotope / palaeoproteomic studies, yielding this type of collagen data.

### 3.3. Collagen screening using Am/P, Am/C_1_, and Am/C_2_

**Fig 4:**
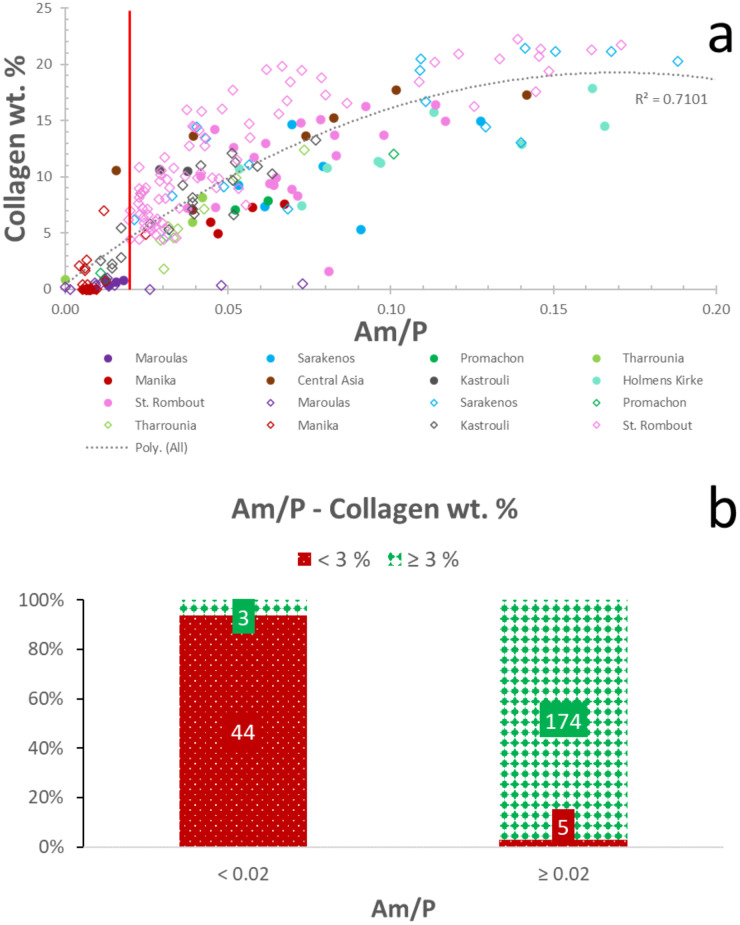
Am/P-collagen content relationship. (a) Collagen preservation shows strong polynomial (order 2) correlation with Am/P. The red line represents the proposed Am/P = 0.02 cut-off point. The circles represent the petrous bones and rhombuses the other skeletal elements. (b) Distribution of samples with well- (≥ 3%; green/solid diamond), and poorly-preserved (< 3%; red/dotted) collagen in categories based on Am/P.

The Am/P ratio can potentially provide valuable information on the relative amount of organic content in bone [58,59,76]. A strong correlation (R^2^ = 0.71; polynomial order = 2) between Am/P and collagen wt. % can be seen in Fig 4 (see also S4 Table), highlighting the potential of Am/P as collagen predictor for rapid screening of archaeological bone. Nonetheless, overtones related to O-H stretching vibrations at 1640–1660 cm^-1^ (water), have been linked to increased collagen estimates [59,62,76].

The relative phosphate content (exogenous phosphate uptake) can also lead to poor agreement of collagen estimates with collagen yields [49,59]. It can be argued that improving the accuracy of the amide I component requires its decoupling from the phosphate signal [49]. However, the use of the peak height at 1640 cm^-1^ after baseline correction (see 2.2.) does not improve the correlation with collagen content (R^2^ = 0.70; polynomial order = 2). What clearly improves collagen screening is the use of a cut-off point of 0.02 for Am/P, which allows the distinction of c. 98% of the samples (n = 174) which display collagen yields ≥ 3% (Fig 4; Table 1). This is combined with an elimination of 90% of specimens (n = 44) that contain < 3% collagen by weight (Fig 4; Table 1). The success rate of the 0.02 Am/P threshold drops only slightly to c. 96% (n = 170) levels for samples with ≥ 2% collagen by weight, while there is no change in the elimination of poorly-preserved archaeological bone.

**Table 1:** Summary of FTIR-ATR screening success rates for DNA and collagen preservation.

DNA screening		Successful identification (%)		Successful elimination (%)	
**Index**	**Threshold**	**Endogenous DNA > 10%**	**Endogenous DNA > 1%**	**Endogenous DNA < 1%**	
IRSF	≤ 4.2 (moderate)	100	100	46	
IRSF	≤ 3.7 (strict)	88	84	67	
C/P	≥ 0.13	100	93	42	
IRSF and C/P*	IRSF ≤ 4.2 C/P ≥ 0.13	100	100	42	
IRSF and C/P*	IRSF ≤ 3.7 C/P ≥ 0.13	92	91	46	
Collagen wt. %	≥ 5%	94	100	84	
Collagen screening		Successful identification (%)		Successful elimination (%)	
**Index**	**Threshold**	**Collagen by weight ≥ 3%**	**Collagen by weight ≥ 2%**	**Collagen by weight < 3%**	**Collagen by weight < 2%**
Am/P	≥ 0.02	98	96	90	90
Am/C_1_	≥ 0.1	99	97	82	84
Am/C_2_	≥ 0.2	97	94	90	92
Am/C_1_ and Am/C_2_*	Am/C_1_ ≥ 0.1 Am/C_2_ ≥ 0.2	99	97	82	84

*If two indices are used in conjunction, only one of the two thresholds need to be satisfied to keep samples, whereas samples need to fail both thresholds for elimination.

However, as nucleation of BAp crystals on collagen fibres can involve the chemical interaction between negatively charged carboxyl groups (COO^-^) and positively charged calcium ions (Ca^2+^) of the calcium carbonate crystals, the peak heights at c. 1410 cm^-1^ and c. 872 cm^-1^ could be potentially used instead of phosphate in a modified Am/X ratio (where X can be either the 1410 cm^-1^ or the 872 cm^-1^ peak height after baseline correction). *In vitro* experiments conducted by Rhee et al. [77] have demonstrated that the crystals formed on the collagen membrane are carbonate-containing hydroxyapatite (HAp) crystals, and the shift of the carboxylate band after nucleation indicated that there was a chemical interaction between the carboxylate group of the collagen and the nucleated HAp crystals.

Indeed, the use of the Am/C_1_ (1640 cm^-1^ / 1410 cm^-1^) with a threshold at 0.1 can identify 99% of samples with collagen wt. % ≥ 3 (S3 Fig), and 97% of samples with collagen wt. % ≥ 2 (Table 1 and S4 Table). This threshold can also eliminate 82% of bones with collagen yields < 3%, and 84% of bones with collagen yields < 2% (Table 1 and S4 Table). On the other hand, the Am/C_2_ ratio (1640 cm^-1^ / 872 cm^-1^) successfully identifies c. 97% of samples with collagen wt. ≥ 3% (S4 Fig), and c. 94% of samples with collagen wt. ≥ 2% with a 0.2 cut-off (Table 1 and S4 Table). This is accompanied by an elimination of c. 90% of samples with poorly preserved collagen (i.e. below 3% collagen wt.), which increases to c. 92% when the cut-off for collagen content is 2% (Table 1). When the Am/C_1_ and Am/C_2_ thresholds are combined, c. 99% of samples containing ≥ 3% collagen by weight or c. 97% of samples containing ≥ 2% collagen by weight can be identified, while eliminating c. 82% of samples containing below 3% collagen by weight or c. 84% of samples containing less than 2% collagen by weight.

Although both indices display strong correlation with collagen yields (R^2^ = 0.73 and R^2^ = 0.72, for Am/C_1_ and Am/C_2_, respectively; polynomial order = 2), post-mortem fragmentation of the polypeptide chains may lead to the survival of peptide sequences containing different numbers of the preserved carboxyl sites that are considered to be involved in cation binding [78]. Similarly, structural differences exist between BAp carbonate and exogenous calcium carbonate polymorphs (e.g. calcite; [79,80]) that might have been incorporated into BAp post-mortem and interact with the carboxyl groups (COO^-^) of collagen.

## 4. Summary

This study proposes the use of FTIR-ATR for the successful identification of archaeological bone samples containing > 1% endogenous DNA, ≥ 3% or ≥ 2% collagen by weight, and establishes thresholds for three commonly used mid-IR indices (IRSF, C/P, Am/P) with significant implications for palaeogenetic and palaeoproteomic research costs. Specifically:

1. The use of a ‘moderate’ cut-off point at 4.2 IRSF successfully identifies 100% of samples with endogenous DNA yields > 10%, 100% of bones with endogenous DNA yields > 1%, while it eliminates about half of samples with endogenous DNA below 1%.
2. The use of a ‘strict’ cut-off point at 3.7 IRSF successfully identifies c. 90% of samples with endogenous DNA yields > 10%, c.80 % of bones with endogenous DNA yields > 1%, while it eliminates c. 70% of samples with endogenous DNA below 1%.
3. The success rates of this approach are not affected by variances in the bioinformatic pipelines followed in the various aDNA labs.
4. The complementary use of already existing collagen yields (cut-off point = 5%; for example when collagen data have already been generated for radiocarbon / stable isotope / palaeoproteomic studies) can further improve screening for endogenous DNA (i.e. successful identification of 100% of bones with endogenous DNA yields > 1%; elimination of c. 85% of the specimens with endogenous DNA < 1%).
5. The use of the Am/P cut-off point at 0.02 allows the successful identification of 98% of the specimens with collagen wt. % ≥ 3%, while it eliminates about 90% of bones with less than 3% collagen yields. Additionally, it allows the successful identification of 96% of the specimens with collagen wt. % ≥ 2%, while it eliminates about 90% of bones with less than 2% collagen yields
6. The combined use of cut-off points at 0.1 for Am/C_1_ and 0.2 for Am/C_2_ allows the successful identification of c. 99% of samples containing over 3% collagen by weight and c. 97% of samples containing over 2% collagen by weight, while it eliminates c. 82% of samples containing below 3% collagen by weight or c. 84% of samples containing below 2% collagen by weight.

## Supporting information

S1 Table List of samples. Skeletal elements, species, origin, archaeological period and chronological age of each sample. The number in the species column denotes the different individuals with more than one sample. L = left; R = right; P = proximal diaphysis; M = mid diaphysis; D = distal diaphysis.
(DOCX)

S2 Table Repository information. * N/A: not available.
(DOCX)

S3 Table Reproducibility and comparability of mid-IR data. Alpha Platinum versus Vertex 70v vacuum FTIR-ATR. * Alpha Platinum; ** Vertex 70v.
(DOCX)

S4 Table FTIR, collagen wt. % and endogenous DNA data. Infrared splitting factor (IRSF), carbonate-to-phosphate (C/P), amide-to-phosphate (Am/P), amide-to-carbonate_1_ (Am/C_1_), amide-to-carbonate_2_ (Am/C_2_). The + symbol next to samples’ names indicate samples that sampling for DNA analysis preceded. The letter next to endogenous DNA yields denotes the ancient DNA lab the data originate, i.e. C = Copenhagen, D = Dublin, and M = Mainz. N/A: not applicable.
(DOCX)

S1 Fig Endogenous DNA-crystallinity relationship. Distribution of samples with well- (> 10%; green/solid diamond), moderately- (1–10%; yellow/large checkerboard), and poorly-preserved (< 1%; red/dotted) endogenous DNA in categories based on crystallinity (n = 85). Endogenous DNA % were estimated using the same bioinformatics pipeline (see section 2—supporting information for details) for all samples to eliminate the potential effects of the different bioinformatics protocols followed by the three different labs on the estimated yields. THA2, THA3, and THA11 samples were not reprocessed, thus excluded from this graph. The c. 90% of the well-preserved specimens with endogenous DNA > 10% (n = 45) display IRSF values < 3.7, and only c. 10% (n = 7) have 3.7 ≤ IRSF ≤ 4.2. The samples that yield endogenous DNA below 1% predominantly display IRSF values over 3.7 (c. 70%; n = 14), with a small subset (c. 30%; n = 6) characterized by crystallinity below the 3.7 threshold. All samples with IRSF > 4.2 have endogenous DNA yields below 1%. Success rates are similar to those reported in the text, suggesting that this screening method is not affected by the bioinformatics pipeline.
(DOCX)

S2 Fig Endogenous DNA-carbonate content relationship. Distribution of samples with well- (> 10%; green/solid diamond), moderately- (1–10%; yellow/large checkerboard), and poorly-preserved (< 1%; red/dotted) endogenous DNA in categories based on carbonate content (n = 85). Endogenous DNA % were also estimated using the same bioinformatics pipeline (see section 2—supporting information for details) for all samples to eliminate the potential effects of the different bioinformatics protocols followed by the three different labs on the estimated yields. THA2, THA3, and THA11 samples were not reprocessed, thus excluded from this graph. The c. 85% of the specimens with endogenous DNA > 1% (n = 64) display C/P values > 0.13, and only c. 15% (n = 11) have endogenous DNA yields below 1%. When C/P drops below 0.13, samples with endogenous DNA yields < 1% prevail (i.e. c. 90%; n = 9), and only c. 10% (n = 1) of samples contain more than 1% endogenous DNA. Success rates are similar to those reported in the text, suggesting that this screening method is not affected by the different bioinformatics pipeline.
(DOCX)

S3 Fig Am/C_1_-collagen content relationship. (a) Collagen preservation shows strong polynomial correlation with Am/C_1_ (R^2^ = 0.73; polynomial order = 2). The red line represents the proposed Am/C_1_ = 0.1 cut-off point. The circles represent the petrous bones and rhombuses the other skeletal elements. (b) Distribution of samples with well- (≥ 3%; green/solid diamond), and poorly-preserved (< 3%; red/dotted) collagen in categories based on Am/C_1_.
(DOCX)

S4 Fig Am/C_2_-collagen content relationship. (a) Collagen preservation shows strong polynomial correlation with Am/C_2_ (R^2^ = 0.72; polynomial order = 2). The red line represents the proposed Am/C_2_ = 0.2 cut-off point. The circles represent the petrous bones and rhombuses the other skeletal elements. (b) Distribution of samples with well- (≥ 3%; green/solid diamond), and poorly-preserved (< 3%; red/dotted) collagen in categories based on Am/C_2_.
(DOCX)

S1 Text (DOCX)
