# Supplementary material for: Screening archaeological bone for palaeogenetic and palaeoproteomic studies
Source: PLoS One. 2020 Jun 25;15(6):e0235146. doi: 10.1371/journal.pone.0235146 (PMC7316274; doi:10.1371/journal.pone.0235146)
Supplement: S1 Table — Skeletal elements, species, origin, archaeological period and chronological age of each sample. The number in the species column denotes the different individuals with more than one sample. L = left; R = right; P = proximal diaphysis; M = mid diaphysis; D = distal diaphysis. (DOCX) [file pone.0235146.s001.docx]

**S1 Table. List of samples.** Skeletal elements, species, origin, archaeological periods and chronological age of each sample. The number in the species column denotes the different individuals with more than one sample. L=left; R=right; P=proximal diaphysis; M=mid diaphysis; D=distal diaphysis.

| **Sample** | **Skeletal element** | **Species** | **Country** | **Site** | **Period** | **Date** |
| --- | --- | --- | --- | --- | --- | --- |
| BED1 | Petrous | Aurochs | Germany | Bedburg-Königshoven | Early Mesolithic | 10000-10400 BC |
| BED2 | Petrous | Aurochs | Germany | Bedburg-Königshoven | Early Mesolithic | 9500-9700 BC |
| BED3 | Petrous | Aurochs | Germany | Bedburg-Königshoven | Early Mesolithic | 9500-9700 BC |
| BED4 | Petrous | Aurochs | Germany | Bedburg-Königshoven | Early Mesolithic | 9600-9900 BC |
| BED9 | Petrous | Aurochs | Germany | Bedburg-Königshoven | Early Mesolithic | 9600-9900 BC |
| MAR1 | R. Petrous | Human 1 | Greece | Maroulas, Kythnos | Mesolithic | 8800-8700 BC |
| MAR2 | Rib | Human 1 | Greece | Maroulas, Kythnos | Mesolithic | 8800-8700 BC |
| MAR3 | R. Clavicle | Human 1 | Greece | Maroulas, Kythnos | Mesolithic | 8800-8700 BC |
| MAR4 | R. Humerus (D) | Human 1 | Greece | Maroulas, Kythnos | Mesolithic | 8800-8700 BC |
| MAR5 | L. Humerus (D) | Human 1 | Greece | Maroulas, Kythnos | Mesolithic | 8800-8700 BC |
| MAR6 | L. Ulna (D) | Human 1 | Greece | Maroulas, Kythnos | Mesolithic | 8800-8700 BC |
| MAR7 | R. Fibula (P) | Human 1 | Greece | Maroulas, Kythnos | Mesolithic | 8800-8700 BC |
| MAR8 | R. Fibula (D) | Human 1 | Greece | Maroulas, Kythnos | Mesolithic | 8800-8700 BC |
| MAR9 | R. Petrous | Human | Greece | Maroulas, Kythnos | Mesolithic | 8800-8700 BC |
| MAR10 | L. Petrous | Human | Greece | Maroulas, Kythnos | Mesolithic | 8800-8700 BC |
| MAR11 | R. Tibia (M) | Human | Greece | Maroulas, Kythnos | Mesolithic | 8800-8700 BC |
| MAR12 | L. Tibia (M) | Human | Greece | Maroulas, Kythnos | Mesolithic | 8800-8700 BC |
| MAR13 | Tibia | Human | Greece | Maroulas, Kythnos | Mesolithic | 8800-8700 BC |
| MAR14 | Femur | Human | Greece | Maroulas, Kythnos | Mesolithic | 8800-8700 BC |
| MAR15 | Humerus | Human | Greece | Maroulas, Kythnos | Mesolithic | 8800-8700 BC |
| MAR16 | Petrous | Human | Greece | Maroulas, Kythnos | Mesolithic | 8800-8700 BC |
| VEM139 | Petrous | Cattle | Jordan | Ain Ghazal | Neolithic | 7500-5500 BC |
| VEM140 | Petrous | Goat | Jordan | Ain Ghazal | Neolithic | 7500-5500 BC |
| VEM141 | Petrous | Sheep | Jordan | Ain Ghazal | Neolithic | 7500-5500 BC |
| VEM143 | Petrous | Sheep | Jordan | Ain Ghazal | Neolithic | 7500-5500 BC |
| SAR24 | R. Petrous | Sheep | Greece | Sarakenos Cave, Boeotia | Early Neolithic | 6400-6000 BC |
| SAR28 | L. Petrous | Sheep | Greece | Sarakenos Cave, Boeotia | Early Neolithic | 6400-6000 BC |
| SAR35 | R. Petrous | Sheep | Greece | Sarakenos Cave, Boeotia | Middle Neolithic | 5750-5600 BC |
| SAR38 | R. Petrous | Cattle | Greece | Sarakenos Cave, Boeotia | Middle Neolithic | 5750-5600 BC |
| SAR1 | Mandible (L. I2) | Human | Greece | Sarakenos Cave, Boeotia | Middle Neolithic | 5750-5600 BC |
| SAR2 | Mandible (L. M2) | Human | Greece | Sarakenos Cave, Boeotia | Late Neolithic Ia | 5000-4800 BC |
| SAR3 | Mandible (L. M1) | Human | Greece | Sarakenos Cave, Boeotia | Late Neolithic Ia | 5000-4800 BC |
| SAR4 | R. Femur (D) | Human | Greece | Sarakenos Cave, Boeotia | Late Neolithic Ia | 5000-4800 BC |
| SAR5 | R. Humerus (P) | Human | Greece | Sarakenos Cave, Boeotia | Late Neolithic Ia | 5000-4800 BC |
| SAR6 | R. Humerus (D) | Human | Greece | Sarakenos Cave, Boeotia | Late Neolithic Ia | 5000-4800 BC |
| SAR7 | R. Tibia (D) | Human | Greece | Sarakenos Cave, Boeotia | Late Neolithic Ia | 5000-4800 BC |
| SAR40 | R. Petrous | Sheep | Greece | Sarakenos Cave, Boeotia | Late Neolithic Ia | 5000-4800 BC |
| SAR8 | R. Petrous | Human | Greece | Sarakenos Cave, Boeotia | Late Neolithic Ib | 4800-4500 BC |
| SAR9 | Mandible (R. M3) | Human | Greece | Sarakenos Cave, Boeotia | Late Neolithic Ib | 4800-4500 BC |
| SAR10 | L. Femur (M) | Human | Greece | Sarakenos Cave, Boeotia | Late Neolithic II | 4300-4000 BC |
| SAR11 | L. Ulna (M) | Human | Greece | Sarakenos Cave, Boeotia | Late Neolithic II | 4300-4000 BC |
| SAR12 | Metatarsal (fragment) | Human | Greece | Sarakenos Cave, Boeotia | Late Neolithic II | 4300-4000 BC |
| SAR13 | L. Femur (P) | Human | Greece | Sarakenos Cave, Boeotia | Late Neolithic II | 4300-4000 BC |
| SAR14 | L. Femur (D) | Human | Greece | Sarakenos Cave, Boeotia | Late Neolithic II | 4300-4000 BC |
| SAR15 | L. Tibia (D) | Human | Greece | Sarakenos Cave, Boeotia | Late Neolithic II | 4300-4000 BC |
| SAR16 | R. Femur (P) | Human | Greece | Sarakenos Cave, Boeotia | Late Neolithic II | 4300-4000 BC |
| SAR17 | R. Femur (D) | Human | Greece | Sarakenos Cave, Boeotia | Late Neolithic II | 4300-4000 BC |
| SAR18 | Mandible (mental spine) | Human | Greece | Sarakenos Cave, Boeotia | LN? | N/A |
| SAR19 | R. Humerus (M) | Human | Greece | Sarakenos Cave, Boeotia | N/A | N/A |
| PRO1 | L. Petrous | Cattle | Greece | Promachon, Serres | Late Neolithic I | 5400-5000 BC |
| PRO2 | R. Petrous | Cattle | Greece | Promachon, Serres | Late Neolithic I | 5400-5000 BC |
| PRO8 | Metacarpal | Cattle | Greece | Promachon, Serres | Late Neolithic I | 5400-5000 BC |
| PRO9 | Femur | Sheep/goat | Greece | Promachon, Serres | Late Neolithic I | 5400-5000 BC |
| THA1 | L. Petrous | Human | Greece | Tharrounia, Euboea | Neolithic | 4300-3300 BC |
| THA2 | L. Petrous | Human | Greece | Tharrounia, Euboea | Neolithic | 4300-3300 BC |
| THA3 | R. Petrous | Human | Greece | Tharrounia, Euboea | Neolithic | 4300-3300 BC |
| THA4 | L. Femur (P) | Human | Greece | Tharrounia, Euboea | Neolithic | 4300-3300 BC |
| THA5 | L. Femur (D) | Human | Greece | Tharrounia, Euboea | Neolithic | 4300-3300 BC |
| THA6 | L. Femur (P) | Human | Greece | Tharrounia, Euboea | Neolithic | 4300-3300 BC |
| THA7 | L. Femur (D) | Human | Greece | Tharrounia, Euboea | Neolithic | 4300-3300 BC |
| THA8 | Unknown element | Human | Greece | Tharrounia, Euboea | Neolithic | 4300-3300 BC |
| THA9 | Tibia | Human | Greece | Tharrounia, Euboea | Neolithic | 4300-3300 BC |
| THA10 | Femur | Human | Greece | Tharrounia, Euboea | Neolithic | 4300-3300 BC |
| THA11 | Femur | Human | Greece | Tharrounia, Euboea | Neolithic | 4300-3300 BC |
| VEM202 | L. Petrous | Cattle 1 | Britain | Ness of Brodgar, Orkney | Neolithic | 3200-2200 BC |
| VEM203 | R. Petrous | Cattle 1 | Britain | Ness of Brodgar, Orkney | Neolithic | 3200-2200 BC |
| VEM204 | Petrous | Red Deer | Britain | Ness of Brodgar, Orkney | Neolithic | 3200-2200 BC |
| VEM205 | Petrous | Cattle | Britain | Ness of Brodgar, Orkney | Neolithic | 3200-2200 BC |
| VEM206 | Petrous | Cattle | Britain | Ness of Brodgar, Orkney | Neolithic | 3200-2200 BC |
| VEM207 | Petrous | Sheep | Britain | Ness of Brodgar, Orkney | Neolithic | 3200-2200 BC |
| VEM208 | Petrous | Sheep | Britain | Ness of Brodgar, Orkney | Neolithic | 3200-2200 BC |
| MAN1 | R. Petrous | Human | Greece | Manika, Euboea | Early Bronze Age | 2900-2300 BC |
| MAN2 | Rib (fragment) | Human | Greece | Manika, Euboea | Early Bronze Age | 2900-2300 BC |
| MAN3 | Femur (M) | Human | Greece | Manika, Euboea | Early Bronze Age | 2900-2300 BC |
| MAN4 | R. Petrous | Human | Greece | Manika, Euboea | Early Bronze Age | 2900-2300 BC |
| MAN5 | L. Petrous | Human | Greece | Manika, Euboea | Early Bronze Age | 2900-2300 BC |
| MAN6 | R. Petrous | Human | Greece | Manika, Euboea | Early Bronze Age | 2900-2300 BC |
| MAN7 | L. Petrous | Human | Greece | Manika, Euboea | Early Bronze Age | 2900-2300 BC |
| MAN8 | R. Humerus (P) | Human | Greece | Manika, Euboea | Early Bronze Age | 2900-2300 BC |
| MAN9 | R. Humerus (D) | Human | Greece | Manika, Euboea | Early Bronze Age | 2900-2300 BC |
| MAN10 | R. Ulna (P) | Human | Greece | Manika, Euboea | Early Bronze Age | 2900-2300 BC |
| MAN11 | R. Ulna (M) | Human | Greece | Manika, Euboea | Early Bronze Age | 2900-2300 BC |
| MAN12 | R. Clavicle (medial end) | Human | Greece | Manika, Euboea | Early Bronze Age | 2900-2300 BC |
| MAN13 | L. Tibia (M) | Human | Greece | Manika, Euboea | Early Bronze Age | 2900-2300 BC |
| MAN14 | R. Fibula (D) | Human | Greece | Manika, Euboea | Early Bronze Age | 2900-2300 BC |
| MAN15 | L. Petrous | Human | Greece | Manika, Euboea | Early Bronze Age | 2900-2300 BC |
| MAN16 | R. Humerus (D) | Human | Greece | Manika, Euboea | Early Bronze Age | 2900-2300 BC |
| MAN17 | Tibia (M) | Human | Greece | Manika, Euboea | Early Bronze Age | 2900-2300 BC |
| MAN18 | R. Humerus (D) | Human | Greece | Manika, Euboea | Early Bronze Age | 2900-2300 BC |
| MAN19 | R. Femur (M) | Human | Greece | Manika, Euboea | Early Bronze Age | 2900-2300 BC |
| MAN20 | R. Tibia (D) | Human | Greece | Manika, Euboea | Early Bronze Age | 2900-2300 BC |
| MAN21 | L. Petrous | Human | Greece | Manika, Euboea | Early Bronze Age | 2900-2300 BC |
| MAN22 | L. Petrous | Human | Greece | Manika, Euboea | Early Bronze Age | 2900-2300 BC |
| MAN23 | R. Femur (P) | Human | Greece | Manika, Euboea | Early Bronze Age | 2900-2300 BC |
| MAN24 | R. Femur (D) | Human | Greece | Manika, Euboea | Early Bronze Age | 2900-2300 BC |
| MAN25 | R. Petrous | Human | Greece | Manika, Euboea | Early Bronze Age | 2900-2300 BC |
| MAN26 | R. Radius (P) | Human | Greece | Manika, Euboea | Early Bronze Age | 2900-2300 BC |
| MAN27 | L. Radius (P) | Human | Greece | Manika, Euboea | Early Bronze Age | 2900-2300 BC |
| MAN28 | Long bone | Human | Greece | Manika, Euboea | Early Bronze Age | 2900-2300 BC |
| MAN29 | Long bone | Human | Greece | Manika, Euboea | Early Bronze Age | 2900-2300 BC |
| MAN30 | R. Radius | Human | Greece | Manika, Euboea | Early Bronze Age | 2900-2300 BC |
| MAN31 | Long bone | Human | Greece | Manika, Euboea | Early Bronze Age | 2900-2300 BC |
| CA1 | Petrous | Human | Central Asia | N/A | Bronze Age | 2100-1800 BC |
| CA2 | Petrous | Human | Central Asia | N/A | Bronze Age | 2100-1800 BC |
| CA3 | Petrous | Human | Central Asia | N/A | Bronze Age | 2100-1800 BC |
| CA4 | Petrous | Human | Central Asia | N/A | Bronze Age | 2100-1800 BC |
| CA5 | Petrous | Human | Central Asia | N/A | Bronze Age | 2100-1800 BC |
| CA6 | Petrous | Human | Central Asia | N/A | Bronze Age | 2100-1800 BC |
| VEM210 | Petrous | Sheep | Britain | Silgenach, South Uist | Bronze Age | 2200-800 BC |
| VEM146 | Petrous | Cattle | Britain | Cladh Hallan, South Uist | Bronze Age | 2200-800 BC |
| VEM147 | Petrous | Sheep | Britain | Cladh Hallan, South Uist | Bronze Age | 2200-800 BC |
| VEM148 | Petrous | Cattle | Britain | Cladh Hallan, South Uist | Bronze Age | 2200-800 BC |
| VEM149 | Petrous | Cattle | Britain | Cladh Hallan, South Uist | Bronze Age | 2200-800 BC |
| VEM178 | Petrous | Cattle | Britain | Cladh Hallan, South Uist | Bronze Age | 2200-800 BC |
| VEM179 | Petrous | Cattle | Britain | Cladh Hallan, South Uist | Bronze Age | 2200-800 BC |
| KAS1 | R. Femur (P) | Human | Greece | Kastrouli, Delphi | Late Bronze Age - Iron Age | 1200-800 BC |
| KAS2 | R. Femur (P) | Human | Greece | Kastrouli, Delphi | Late Bronze Age - Iron Age | 1200-800 BC |
| KAS3 | R. Femur (P) | Human | Greece | Kastrouli, Delphi | Late Bronze Age - Iron Age | 1200-800 BC |
| KAS4 | R. Femur (P) | Human | Greece | Kastrouli, Delphi | Late Bronze Age - Iron Age | 1200-800 BC |
| KAS5 | R. Femur (P) | Human | Greece | Kastrouli, Delphi | Late Bronze Age - Iron Age | 1200-800 BC |
| KAS6 | R. Femur (P) | Human | Greece | Kastrouli, Delphi | Late Bronze Age - Iron Age | 1200-800 BC |
| KAS7 | R. Femur (P) | Human | Greece | Kastrouli, Delphi | Late Bronze Age - Iron Age | 1200-800 BC |
| KAS8 | R. Femur (P) | Human | Greece | Kastrouli, Delphi | Late Bronze Age - Iron Age | 1200-800 BC |
| KAS9 | R. Femur (P) | Human | Greece | Kastrouli, Delphi | Late Bronze Age - Iron Age | 1200-800 BC |
| KAS10 | R. Femur (P) | Human | Greece | Kastrouli, Delphi | Late Bronze Age - Iron Age | 1200-800 BC |
| KAS11 | R. Femur (P) | Human | Greece | Kastrouli, Delphi | Late Bronze Age - Iron Age | 1200-800 BC |
| KAS12 | R. Femur (P) | Human | Greece | Kastrouli, Delphi | Late Bronze Age - Iron Age | 1200-800 BC |
| KAS13 | R. Femur (P) | Human | Greece | Kastrouli, Delphi | Late Bronze Age - Iron Age | 1200-800 BC |
| KAS14 | R. Femur (P) | Human | Greece | Kastrouli, Delphi | Late Bronze Age - Iron Age | 1200-800 BC |
| KAS15 | R. Femur (P) | Human | Greece | Kastrouli, Delphi | Late Bronze Age - Iron Age | 1200-800 BC |
| KAS16 | L. Petrous | Human | Greece | Kastrouli, Delphi | Late Bronze Age - Iron Age | 1200-800 BC |
| KAS17 | R. Petrous | Human | Greece | Kastrouli, Delphi | Late Bronze Age - Iron Age | 1200-800 BC |
| KAS18 | R. Carpometacarpus | Chicken | Greece | Kastrouli, Delphi | Late Bronze Age - Iron Age | 1200-800 BC |
| KAS19 | R. Carpometacarpus (D) | Chicken | Greece | Kastrouli, Delphi | Late Bronze Age - Iron Age | 1200-800 BC |
| KAS22 | Phalanx | Cattle | Greece | Kastrouli, Delphi | Late Bronze Age - Iron Age | 1200-800 BC |
| KAS23 | L. Humerus (D) | Pig | Greece | Kastrouli, Delphi | Late Bronze Age - Iron Age | 1200-800 BC |
| KAS26 | R. Calcaneus | Cattle | Greece | Kastrouli, Delphi | Late Bronze Age - Iron Age | 1200-800 BC |
| KAS28 | Phalanx | Sheep/goat | Greece | Kastrouli, Delphi | Late Bronze Age - Iron Age | 1200-800 BC |
| KAS29 | Long Bone | Sheep/goat | Greece | Kastrouli, Delphi | Late Bronze Age - Iron Age | 1200-800 BC |
| VEM193 | Petrous | Cattle | Britain | Potterne, Wiltshire | Late Bronze Age - Iron Age | 1450-550 BC |
| VEM194 | Petrous | Cattle | Britain | Potterne, Wiltshire | Late Bronze Age - Iron Age | 1450-550 BC |
| VEM195 | Petrous | Cattle | Britain | Potterne, Wiltshire | Late Bronze Age - Iron Age | 1450-550 BC |
| VEM196 | Petrous | Cattle | Britain | Potterne, Wiltshire | Late Bronze Age - Iron Age | 1450-550 BC |
| VEM197 | Petrous | Cattle | Britain | Potterne, Wiltshire | Late Bronze Age - Iron Age | 1450-550 BC |
| VEM198 | Petrous | Cattle | Britain | Potterne, Wiltshire | Late Bronze Age - Iron Age | 1450-550 BC |
| VEM201 | Petrous | Sheep | Britain | Potterne, Wiltshire | Late Bronze Age - Iron Age | 1450-550 BC |
| VEM180 | Petrous | Cattle | Britain | Danebury, Hampshire | Iron Age | 500-100 BC |
| VEM181 | Petrous | Cattle | Britain | Danebury, Hampshire | Iron Age | 500-100 BC |
| VEM182 | Petrous | Cattle | Britain | Danebury, Hampshire | Iron Age | 500-100 BC |
| VEM100 | Petrous | Cattle | Britain | Tanner Row, York | Roman | 100-200 AD |
| VEM209 | Petrous | Sheep | Britain | Bornais, South Uist | Iron Age | 400-600 AD |
| VEM101 | Petrous | Cattle | Britain | Coppergate, York | Medieval | 800-900 AD |
| VEM108 | Petrous | Cattle | Britain | Hungate, York | Medieval | 900-1000 AD |
| VEM111 | Petrous | Cattle | Britain | Coppergate, York | Medieval | 900-1000 AD |
| VEM102 | Petrous | Cattle | Britain | Hungate, York | Medieval | 1200-1300 AD |
| VEM103 | Petrous | Cattle | Britain | Hungate, York | Medieval | 1200-1400 AD |
| MEC1 | L. Petrous | Human 1 | Belgium | St. Rombout, Mechelen | Middle Ages | 900-1800 AD |
| MEC2 | R. Femur (D) | Human 1 | Belgium | St. Rombout, Mechelen | Middle Ages | 900-1800 AD |
| MEC3 | R. Tibia (P) | Human 1 | Belgium | St. Rombout, Mechelen | Middle Ages | 900-1800 AD |
| MEC4 | R. Tibia (D) | Human 1 | Belgium | St. Rombout, Mechelen | Middle Ages | 900-1800 AD |
| MEC5 | R. Petrous | Human 2 | Belgium | St. Rombout, Mechelen | Middle Ages | 900-1800 AD |
| MEC6 | L. Humerus (P) | Human 2 | Belgium | St. Rombout, Mechelen | Middle Ages | 900-1800 AD |
| MEC7 | L. Humerus (D) | Human 2 | Belgium | St. Rombout, Mechelen | Middle Ages | 900-1800 AD |
| MEC8 | L. Radius (P) | Human 2 | Belgium | St. Rombout, Mechelen | Middle Ages | 900-1800 AD |
| MEC9 | L. Radius (D) | Human 2 | Belgium | St. Rombout, Mechelen | Middle Ages | 900-1800 AD |
| MEC10 | R. Petrous | Human 3 | Belgium | St. Rombout, Mechelen | Middle Ages | 900-1800 AD |
| MEC11 | L. Femur (D) | Human 3 | Belgium | St. Rombout, Mechelen | Middle Ages | 900-1800 AD |
| MEC12 | L. Femur (P) | Human 3 | Belgium | St. Rombout, Mechelen | Middle Ages | 900-1800 AD |
| MEC13 | L. Tibia (P) | Human 3 | Belgium | St. Rombout, Mechelen | Middle Ages | 900-1800 AD |
| MEC14 | L. Tibia (D) | Human 3 | Belgium | St. Rombout, Mechelen | Middle Ages | 900-1800 AD |
| MEC15 | L. Petrous | Human 4 | Belgium | St. Rombout, Mechelen | Middle Ages | 900-1800 AD |
| MEC16 | L. Humerus (P) | Human 4 | Belgium | St. Rombout, Mechelen | Middle Ages | 900-1800 AD |
| MEC17 | L. Humerus (D) | Human 4 | Belgium | St. Rombout, Mechelen | Middle Ages | 900-1800 AD |
| MEC18 | L. Radius (P) | Human 4 | Belgium | St. Rombout, Mechelen | Middle Ages | 900-1800 AD |
| MEC19 | L. Radius (D) | Human 4 | Belgium | St. Rombout, Mechelen | Middle Ages | 900-1800 AD |
| MEC20 | L. Petrous | Human 5 | Belgium | St. Rombout, Mechelen | Middle Ages | 900-1800 AD |
| MEC21 | L. Humerus (P) | Human 5 | Belgium | St. Rombout, Mechelen | Middle Ages | 900-1800 AD |
| MEC22 | L. Humerus (D) | Human 5 | Belgium | St. Rombout, Mechelen | Middle Ages | 900-1800 AD |
| MEC23 | L. Radius (P) | Human 5 | Belgium | St. Rombout, Mechelen | Middle Ages | 900-1800 AD |
| MEC24 | L. Radius (D) | Human 5 | Belgium | St. Rombout, Mechelen | Middle Ages | 900-1800 AD |
| MEC25 | R. Petrous | Human 6 | Belgium | St. Rombout, Mechelen | Middle Ages | 900-1800 AD |
| MEC26 | L. Femur (P) | Human 6 | Belgium | St. Rombout, Mechelen | Middle Ages | 900-1800 AD |
| MEC27 | L. Femur (D) | Human 6 | Belgium | St. Rombout, Mechelen | Middle Ages | 900-1800 AD |
| MEC28 | L. Tibia (P) | Human 6 | Belgium | St. Rombout, Mechelen | Middle Ages | 900-1800 AD |
| MEC29 | L. Tibia (D) | Human 6 | Belgium | St. Rombout, Mechelen | Middle Ages | 900-1800 AD |
| MEC30 | L. Petrous | Human 7 | Belgium | St. Rombout, Mechelen | Middle Ages | 900-1800 AD |
| MEC31 | L. Humerus (P) | Human 7 | Belgium | St. Rombout, Mechelen | Middle Ages | 900-1800 AD |
| MEC32 | L. Humerus (D) | Human 7 | Belgium | St. Rombout, Mechelen | Middle Ages | 900-1800 AD |
| MEC33 | L. Radius (P) | Human 7 | Belgium | St. Rombout, Mechelen | Middle Ages | 900-1800 AD |
| MEC34 | L. Radius (D) | Human 7 | Belgium | St. Rombout, Mechelen | Middle Ages | 900-1800 AD |
| MEC35 | R. Petrous | Human 8 | Belgium | St. Rombout, Mechelen | Middle Ages | 900-1800 AD |
| MEC36 | R. Humerus (P) | Human 8 | Belgium | St. Rombout, Mechelen | Middle Ages | 900-1800 AD |
| MEC37 | R. Humerus (D) | Human 8 | Belgium | St. Rombout, Mechelen | Middle Ages | 900-1800 AD |
| MEC38 | R. Radius (P) | Human 8 | Belgium | St. Rombout, Mechelen | Middle Ages | 900-1800 AD |
| MEC39 | R. Radius (D) | Human 8 | Belgium | St. Rombout, Mechelen | Middle Ages | 900-1800 AD |
| MEC40 | L. Petrous | Human 9 | Belgium | St. Rombout, Mechelen | Middle Ages | 900-1800 AD |
| MEC41 | L. Humerus (D) | Human 9 | Belgium | St. Rombout, Mechelen | Middle Ages | 900-1800 AD |
| MEC42 | L. Radius (P) | Human 9 | Belgium | St. Rombout, Mechelen | Middle Ages | 900-1800 AD |
| MEC43 | L. Radius (D) | Human 9 | Belgium | St. Rombout, Mechelen | Middle Ages | 900-1800 AD |
| MEC44 | R. Petrous | Human 10 | Belgium | St. Rombout, Mechelen | Middle Ages | 900-1800 AD |
| MEC45 | R. Humerus (P) | Human 10 | Belgium | St. Rombout, Mechelen | Middle Ages | 900-1800 AD |
| MEC46 | R. Humerus (D) | Human 10 | Belgium | St. Rombout, Mechelen | Middle Ages | 900-1800 AD |
| MEC47 | R. Radius (P) | Human 10 | Belgium | St. Rombout, Mechelen | Middle Ages | 900-1800 AD |
| MEC48 | R. Radius (D) | Human 10 | Belgium | St. Rombout, Mechelen | Middle Ages | 900-1800 AD |
| MEC49 | L. Tibia (P) | Human 11 | Belgium | St. Rombout, Mechelen | Middle Ages | 900-1800 AD |
| MEC50 | L. Tibia (D) | Human 11 | Belgium | St. Rombout, Mechelen | Middle Ages | 900-1800 AD |
| MEC51 | L. Femur (D) | Human 11 | Belgium | St. Rombout, Mechelen | Middle Ages | 900-1800 AD |
| MEC52 | L. Femur (P) | Human 11 | Belgium | St. Rombout, Mechelen | Middle Ages | 900-1800 AD |
| MEC53 | L. Petrous | Human 11 | Belgium | St. Rombout, Mechelen | Middle Ages | 900-1800 AD |
| MEC54 | R. Petrous | Human 12 | Belgium | St. Rombout, Mechelen | Middle Ages | 900-1800 AD |
| MEC55 | R. Femur (P) | Human 12 | Belgium | St. Rombout, Mechelen | Middle Ages | 900-1800 AD |
| MEC56 | R. Femur (D) | Human 12 | Belgium | St. Rombout, Mechelen | Middle Ages | 900-1800 AD |
| MEC57 | R. Tibia (P) | Human 12 | Belgium | St. Rombout, Mechelen | Middle Ages | 900-1800 AD |
| MEC58 | R. Tibia (D) | Human 12 | Belgium | St. Rombout, Mechelen | Middle Ages | 900-1800 AD |
| MEC59 | R. Petrous | Human 13 | Belgium | St. Rombout, Mechelen | Middle Ages | 900-1800 AD |
| MEC60 | R. Humerus (P) | Human 13 | Belgium | St. Rombout, Mechelen | Middle Ages | 900-1800 AD |
| MEC61 | R. Humerus (D) | Human 13 | Belgium | St. Rombout, Mechelen | Middle Ages | 900-1800 AD |
| MEC62 | R. Radius (P) | Human 13 | Belgium | St. Rombout, Mechelen | Middle Ages | 900-1800 AD |
| MEC63 | R. Radius (D) | Human 13 | Belgium | St. Rombout, Mechelen | Middle Ages | 900-1800 AD |
| MEC64 | R. Petrous | Human 14 | Belgium | St. Rombout, Mechelen | Middle Ages | 900-1800 AD |
| MEC65 | R. Humerus (P) | Human 14 | Belgium | St. Rombout, Mechelen | Middle Ages | 900-1800 AD |
| MEC66 | R. Humerus (D) | Human 14 | Belgium | St. Rombout, Mechelen | Middle Ages | 900-1800 AD |
| MEC67 | R. Radius (P) | Human 14 | Belgium | St. Rombout, Mechelen | Middle Ages | 900-1800 AD |
| MEC68 | R. Radius (D) | Human 14 | Belgium | St. Rombout, Mechelen | Middle Ages | 900-1800 AD |
| MEC69 | R. Petrous | Human 15 | Belgium | St. Rombout, Mechelen | Middle Ages | 900-1800 AD |
| MEC70 | R. Humerus (P) | Human 15 | Belgium | St. Rombout, Mechelen | Middle Ages | 900-1800 AD |
| MEC71 | R. Humerus (D) | Human 15 | Belgium | St. Rombout, Mechelen | Middle Ages | 900-1800 AD |
| MEC72 | R. UIna (P) | Human 15 | Belgium | St. Rombout, Mechelen | Middle Ages | 900-1800 AD |
| MEC73 | R. Ulna (D) | Human 15 | Belgium | St. Rombout, Mechelen | Middle Ages | 900-1800 AD |
| MEC74 | L. Petrous | Human 16 | Belgium | St. Rombout, Mechelen | Middle Ages | 900-1800 AD |
| MEC75 | L. Femur (P) | Human 16 | Belgium | St. Rombout, Mechelen | Middle Ages | 900-1800 AD |
| MEC76 | L. Femur (D) | Human 16 | Belgium | St. Rombout, Mechelen | Middle Ages | 900-1800 AD |
| MEC77 | L. Tibia (P) | Human 16 | Belgium | St. Rombout, Mechelen | Middle Ages | 900-1800 AD |
| MEC78 | L. Tibia (D) | Human 16 | Belgium | St. Rombout, Mechelen | Middle Ages | 900-1800 AD |
| MEC79 | R. Petrous | Human 17 | Belgium | St. Rombout, Mechelen | Middle Ages | 900-1800 AD |
| MEC80 | L. Humerus (P) | Human 17 | Belgium | St. Rombout, Mechelen | Middle Ages | 900-1800 AD |
| MEC81 | L. Humerus (D) | Human 17 | Belgium | St. Rombout, Mechelen | Middle Ages | 900-1800 AD |
| MEC82 | L. Ulna (P) | Human 17 | Belgium | St. Rombout, Mechelen | Middle Ages | 900-1800 AD |
| MEC83 | L. Ulna (D) | Human 17 | Belgium | St. Rombout, Mechelen | Middle Ages | 900-1800 AD |
| MEC84 | R. Petrous | Human 18 | Belgium | St. Rombout, Mechelen | Middle Ages | 900-1800 AD |
| MEC85 | R. Fibula (P) | Human 18 | Belgium | St. Rombout, Mechelen | Middle Ages | 900-1800 AD |
| MEC86 | R. Fibula (D) | Human 18 | Belgium | St. Rombout, Mechelen | Middle Ages | 900-1800 AD |
| MEC87 | R. Tibia (D) | Human 18 | Belgium | St. Rombout, Mechelen | Middle Ages | 900-1800 AD |
| MEC88 | R. Petrous | Human 19 | Belgium | St. Rombout, Mechelen | Middle Ages | 900-1800 AD |
| MEC89 | L. Humerus (P) | Human 19 | Belgium | St. Rombout, Mechelen | Middle Ages | 900-1800 AD |
| MEC90 | L. Humerus (D) | Human 19 | Belgium | St. Rombout, Mechelen | Middle Ages | 900-1800 AD |
| MEC91 | L. Ulna (D) | Human 19 | Belgium | St. Rombout, Mechelen | Middle Ages | 900-1800 AD |
| MEC92 | L. Ulna (P) | Human 19 | Belgium | St. Rombout, Mechelen | Middle Ages | 900-1800 AD |
| MEC93 | R. Petrous | Human 20 | Belgium | St. Rombout, Mechelen | Middle Ages | 900-1800 AD |
| MEC94 | R. Humerus (P) | Human 20 | Belgium | St. Rombout, Mechelen | Middle Ages | 900-1800 AD |
| MEC95 | R. Humerus (D) | Human 20 | Belgium | St. Rombout, Mechelen | Middle Ages | 900-1800 AD |
| MEC96 | R. UIna (P) | Human 20 | Belgium | St. Rombout, Mechelen | Middle Ages | 900-1800 AD |
| MEC97 | R. Ulna (D) | Human 20 | Belgium | St. Rombout, Mechelen | Middle Ages | 900-1800 AD |
| MEC98 | L. Petrous | Human 21 | Belgium | St. Rombout, Mechelen | Middle Ages | 900-1800 AD |
| MEC99 | L. Humerus (P) | Human 21 | Belgium | St. Rombout, Mechelen | Middle Ages | 900-1800 AD |
| MEC100 | L. Ulna (P) | Human 21 | Belgium | St. Rombout, Mechelen | Middle Ages | 900-1800 AD |
| MEC101 | L. Ulna (D) | Human 21 | Belgium | St. Rombout, Mechelen | Middle Ages | 900-1800 AD |
| DEN 1 | Petrous | Human | Denmark | Holmens Kirke, Copenhagen | Historical Period | 1650-1850 AD |
| DEN 2 | Petrous | Human | Denmark | Holmens Kirke, Copenhagen | Historical Period | 1650-1850 AD |
| DEN 3 | Petrous | Human | Denmark | Holmens Kirke, Copenhagen | Historical Period | 1650-1850 AD |
| DEN 4 | Petrous | Human | Denmark | Holmens Kirke, Copenhagen | Historical Period | 1650-1850 AD |
| DEN 5 | Petrous | Human | Denmark | Holmens Kirke, Copenhagen | Historical Period | 1650-1850 AD |
| DEN 6 | Petrous | Human | Denmark | Holmens Kirke, Copenhagen | Historical Period | 1650-1850 AD |
| DEN 7 | Petrous | Human | Denmark | Holmens Kirke, Copenhagen | Historical Period | 1650-1850 AD |
| DEN 8 | Petrous | Human | Denmark | Holmens Kirke, Copenhagen | Historical Period | 1650-1850 AD |
| DEN 9 | Petrous | Human | Denmark | Holmens Kirke, Copenhagen | Historical Period | 1650-1850 AD |
