# Supplementary material for: Screening archaeological bone for palaeogenetic and palaeoproteomic studies
Source: PLoS One. 2020 Jun 25;15(6):e0235146. doi: 10.1371/journal.pone.0235146 (PMC7316274; doi:10.1371/journal.pone.0235146)
Supplement: S2 Table — * N/A: not available. (DOCX) [file pone.0235146.s002.docx]

**S2 Table. Repository information**. *N/A: not available

| **Sample** | **Sample information (e.g.crate/bag/code)** | **Repository (institution and country)** | **Contact** |
| --- | --- | --- | --- |
| BED1 | 96/105‐2; Ind.5 | Rheinisches Landesmuseum (archive Meckenheim), Trier, Germany | Birgit Gehlen; Martin Street |
| BED2 | 87/108‐4 Refits to 87/109‐2; Ind.4 | Rheinisches Landesmuseum (archive Meckenheim), Trier, Germany | Birgit Gehlen; Martin Street |
| BED3 | 104/102‐1 (Streu 33); Ind.8 | Rheinisches Landesmuseum (archive Meckenheim), Trier, Germany | Birgit Gehlen; Martin Street |
| BED4 | 97/107 Refits to 97/107 ‐5,6,7; Ind.3 | Rheinisches Landesmuseum (archive Meckenheim), Trier, Germany | Birgit Gehlen; Martin Street |
| BED9 | 93/106‐2; Ind.6 | Rheinisches Landesmuseum (archive Meckenheim), Trier, Germany | Birgit Gehlen; Martin Street |
| MAR1 | Intact burial | Ephorate of Palaeoanthropology and Speleology, Athens, Greece | Adamantios Sampson |
| MAR2 | Intact burial | Ephorate of Palaeoanthropology and Speleology, Athens, Greece | Adamantios Sampson |
| MAR3 | Intact burial | Ephorate of Palaeoanthropology and Speleology, Athens, Greece | Adamantios Sampson |
| MAR4 | Intact burial | Ephorate of Palaeoanthropology and Speleology, Athens, Greece | Adamantios Sampson |
| MAR5 | Intact burial | Ephorate of Palaeoanthropology and Speleology, Athens, Greece | Adamantios Sampson |
| MAR6 | Intact burial | Ephorate of Palaeoanthropology and Speleology, Athens, Greece | Adamantios Sampson |
| MAR7 | Intact burial | Ephorate of Palaeoanthropology and Speleology, Athens, Greece | Adamantios Sampson |
| MAR8 | Intact burial | Ephorate of Palaeoanthropology and Speleology, Athens, Greece | Adamantios Sampson |
| MAR9 | Burials 21-23, Cranium 1, 06 | Ephorate of Palaeoanthropology and Speleology, Athens, Greece | Adamantios Sampson |
| MAR10 | Cranium 2/06 | Ephorate of Palaeoanthropology and Speleology, Athens, Greece | Adamantios Sampson |
| MAR11 | Burials 21-23 | Ephorate of Palaeoanthropology and Speleology, Athens, Greece | Adamantios Sampson |
| MAR12 | Burials 21-23 | Ephorate of Palaeoanthropology and Speleology, Athens, Greece | Adamantios Sampson |
| MAR13 | T Tomi? 13 1A, male 21-23, section I | Ephorate of Palaeoanthropology and Speleology, Athens, Greece | Adamantios Sampson |
| MAR14 | Tomi 1 burial 21-23 | Ephorate of Palaeoanthropology and Speleology, Athens, Greece | Adamantios Sampson |
| MAR15 | Tomi 1 burial 21-23 | Ephorate of Palaeoanthropology and Speleology, Athens, Greece | Adamantios Sampson |
| MAR16 | Burials 21-23, cranio 3, Tom-1 | Ephorate of Palaeoanthropology and Speleology, Athens, Greece | Adamantios Sampson |
| VEM139 | N/A | Institute of Archaeoogy, University College London, London, United Kingdom | Louise Martin |
| VEM140 | N/A | Institute of Archaeoogy, University College London, London, United Kingdom | Louise Martin |
| VEM141 | N/A | Institute of Archaeoogy, University College London, London, United Kingdom | Louise Martin |
| VEM143 | N/A | Institute of Archaeoogy, University College London, London, United Kingdom | Louise Martin |
| SAR24 | F35 – 31,32 | Ephorate of antiquities of Boeotia (Sarakenos Cave collection), Thebes, Greece | Adamantios Sampson |
| SAR28 | F35 – 31,32 | Ephorate of antiquities of Boeotia (Sarakenos Cave collection), Thebes, Greece | Adamantios Sampson |
| SAR35 | F27 – 48 | Ephorate of antiquities of Boeotia (Sarakenos Cave collection), Thebes, Greece | Adamantios Sampson |
| SAR38 | F22 – 46 | Ephorate of antiquities of Boeotia (Sarakenos Cave collection), Thebes, Greece | Adamantios Sampson |
| SAR1 | F18 – 44 | Ephorate of antiquities of Boeotia (Sarakenos Cave collection), Thebes, Greece | Adamantios Sampson |
| SAR2 | D8 | Ephorate of antiquities of Boeotia (Sarakenos Cave collection), Thebes, Greece | Adamantios Sampson |
| SAR3 | D2 – 3,4 | Ephorate of antiquities of Boeotia (Sarakenos Cave collection), Thebes, Greece | Adamantios Sampson |
| SAR4 | F11 – 40 | Ephorate of antiquities of Boeotia (Sarakenos Cave collection), Thebes, Greece | Adamantios Sampson |
| SAR5 | E10 – 23 | Ephorate of antiquities of Boeotia (Sarakenos Cave collection), Thebes, Greece | Adamantios Sampson |
| SAR6 | E10 – 23 | Ephorate of antiquities of Boeotia (Sarakenos Cave collection), Thebes, Greece | Adamantios Sampson |
| SAR7 | E10 – 29 | Ephorate of antiquities of Boeotia (Sarakenos Cave collection), Thebes, Greece | Adamantios Sampson |
| SAR40 | F18 | Ephorate of antiquities of Boeotia (Sarakenos Cave collection), Thebes, Greece | Adamantios Sampson |
| SAR8 | G8 – 13 | Ephorate of antiquities of Boeotia (Sarakenos Cave collection), Thebes, Greece | Adamantios Sampson |
| SAR9 | H11,13 | Ephorate of antiquities of Boeotia (Sarakenos Cave collection), Thebes, Greece | Adamantios Sampson |
| SAR10 | G1 – 26 | Ephorate of antiquities of Boeotia (Sarakenos Cave collection), Thebes, Greece | Adamantios Sampson |
| SAR11 | E11,12 – 3,34 | Ephorate of antiquities of Boeotia (Sarakenos Cave collection), Thebes, Greece | Adamantios Sampson |
| SAR12 | E12,13 – 43,49 | Ephorate of antiquities of Boeotia (Sarakenos Cave collection), Thebes, Greece | Adamantios Sampson |
| SAR13 | E15 – 43 | Ephorate of antiquities of Boeotia (Sarakenos Cave collection), Thebes, Greece | Adamantios Sampson |
| SAR14 | E15 – 43 | Ephorate of antiquities of Boeotia (Sarakenos Cave collection), Thebes, Greece | Adamantios Sampson |
| SAR15 | E1,2 – 28 | Ephorate of antiquities of Boeotia (Sarakenos Cave collection), Thebes, Greece | Adamantios Sampson |
| SAR16 | E3 – 17 | Ephorate of antiquities of Boeotia (Sarakenos Cave collection), Thebes, Greece | Adamantios Sampson |
| SAR17 | E3 – 17 | Ephorate of antiquities of Boeotia (Sarakenos Cave collection), Thebes, Greece | Adamantios Sampson |
| SAR18 | E(?) | Ephorate of antiquities of Boeotia (Sarakenos Cave collection), Thebes, Greece | Adamantios Sampson |
| SAR19 | A – 3 | Ephorate of antiquities of Boeotia (Sarakenos Cave collection), Thebes, Greece | Adamantios Sampson |
| PRO1 | Γ6 – #14 676-8/130-3 78,90 | Ephorate of antiquities of Serres (Promachon repository), Promachon, Serres, Greece | Chaido Koukouli-Chrysanthaki; Georgios Kazantzis |
| PRO2 | Γ6 – #15 131-3/675-7 | Ephorate of antiquities of Serres (Promachon repository), Promachon, Serres, Greece | Chaido Koukouli-Chrysanthaki; Georgios Kazantzis |
| PRO8 | Γ4 – #13 677-9/132-4 79,10 (53829) | Ephorate of antiquities of Serres (Promachon repository), Promachon, Serres, Greece | Chaido Koukouli-Chrysanthaki; Georgios Kazantzis |
| PRO9 | Γ4 – #13 677-9/132-4 79,10 (54045) | Ephorate of antiquities of Serres (Promachon repository), Promachon, Serres, Greece | Chaido Koukouli-Chrysanthaki; Georgios Kazantzis |
| THA1 | Sections Α,Β,Γ - No.1 - Burial 3 (cemetery) | Department of Biology, National and Kapodistrian University of Athens, Athens, Greece | Adamantios Sampson |
| THA2 | Sections Α,Β,Γ - No.1 - Burial 3 (cemetery) | Department of Biology, National and Kapodistrian University of Athens, Athens, Greece | Adamantios Sampson |
| THA3 | Sections Α,Β,Γ - No.1 - Burial 3 (cemetery) | Department of Biology, National and Kapodistrian University of Athens, Athens, Greece | Adamantios Sampson |
| THA4 | Burial 4 (cemetery) – no.6, unlabelled TIV | Department of Biology, National and Kapodistrian University of Athens, Athens, Greece | Adamantios Sampson |
| THA5 | Burial 4 (cemetery) – no.6, unlabelled TIV | Department of Biology, National and Kapodistrian University of Athens, Athens, Greece | Adamantios Sampson |
| THA6 | Burial 4 (cemetery) – no.6, unlabelled TIV | Department of Biology, National and Kapodistrian University of Athens, Athens, Greece | Adamantios Sampson |
| THA7 | Burial 4 (cemetery) – no.6, unlabelled TIV | Department of Biology, National and Kapodistrian University of Athens, Athens, Greece | Adamantios Sampson |
| THA8 | Grave 5 – Bag A | Department of Biology, National and Kapodistrian University of Athens, Athens, Greece | Adamantios Sampson |
| THA9 | Cave Skoteini B7 | Department of Biology, National and Kapodistrian University of Athens, Athens, Greece | Adamantios Sampson |
| THA10 | Grave 1 | Department of Biology, National and Kapodistrian University of Athens, Athens, Greece | Adamantios Sampson |
| THA11 | Grave 4 | Department of Biology, National and Kapodistrian University of Athens, Athens, Greece | Adamantios Sampson |
| VEM202 | N/A | Trinity College Dublin, Dublin, Ireland | Nick Card; Ingrid Mainland |
| VEM203 | N/A | Trinity College Dublin, Dublin, Ireland | Nick Card; Ingrid Mainland |
| VEM204 | N/A | Trinity College Dublin, Dublin, Ireland | Nick Card; Ingrid Mainland |
| VEM205 | N/A | Trinity College Dublin, Dublin, Ireland | Nick Card; Ingrid Mainland |
| VEM206 | N/A | Trinity College Dublin, Dublin, Ireland | Nick Card; Ingrid Mainland |
| VEM207 | N/A | Trinity College Dublin, Dublin, Ireland | Nick Card; Ingrid Mainland |
| VEM208 | N/A | Trinity College Dublin, Dublin, Ireland | Nick Card; Ingrid Mainland |
| VEM209 | N/A | Trinity College Dublin, Dublin, Ireland | Mike Parker Pearson; Niall Sharples |
| VEM210 | N/A | Trinity College Dublin, Dublin, Ireland | Mike Parker Pearson; Niall Sharples |
| MAN1 | Container 1 – Beligianni estate T.51 (No.8 - MN/BE/T52/001-006) | Department of Biology, National and Kapodistrian University of Athens, Athens, Greece | Adamantios Sampson |
| MAN2 | Container 1 – Beligianni estate T.51 (No.15 - MN/BE/T52/017) | Department of Biology, National and Kapodistrian University of Athens, Athens, Greece | Adamantios Sampson |
| MAN3 | Container 1 – Beligianni estate T.51 (Νο.17 - ΜΝ/ΒΕ/Τ52/021) | Department of Biology, National and Kapodistrian University of Athens, Athens, Greece | Adamantios Sampson |
| MAN4 | Container 2 – Beligianni estate T.54 (No. 1 MN/BE/T54/001-006) | Department of Biology, National and Kapodistrian University of Athens, Athens, Greece | Adamantios Sampson |
| MAN5 | Container 2 – Beligianni estate T.54 (No.7 - MN/BE/Τ54/019-020) | Department of Biology, National and Kapodistrian University of Athens, Athens, Greece | Adamantios Sampson |
| MAN6 | Container 2 – Beligianni estate T.54 (No.7 - MN/BE/Τ54/019-020) | Department of Biology, National and Kapodistrian University of Athens, Athens, Greece | Adamantios Sampson |
| MAN7 | Container 2 – Beligianni estate T.54 (No.7 - MN/BE/Τ54/019-020) | Department of Biology, National and Kapodistrian University of Athens, Athens, Greece | Adamantios Sampson |
| MAN8 | Container 2 – Beligianni estate T.54 | Department of Biology, National and Kapodistrian University of Athens, Athens, Greece | Adamantios Sampson |
| MAN9 | Container 2 – Beligianni estate T.54 | Department of Biology, National and Kapodistrian University of Athens, Athens, Greece | Adamantios Sampson |
| MAN10 | Container 2 – Beligianni estate T.54 | Department of Biology, National and Kapodistrian University of Athens, Athens, Greece | Adamantios Sampson |
| MAN11 | Container 2 – Beligianni estate T.54 | Department of Biology, National and Kapodistrian University of Athens, Athens, Greece | Adamantios Sampson |
| MAN12 | Container 2 – Beligianni estate T.54 | Department of Biology, National and Kapodistrian University of Athens, Athens, Greece | Adamantios Sampson |
| MAN13 | Container 2 – Beligianni estate T.54 (No.3 - MN/BE/Τ54/014-015) | Department of Biology, National and Kapodistrian University of Athens, Athens, Greece | Adamantios Sampson |
| MAN14 | Container 2 – Beligianni estate T.54 (No.3 - MN/BE/Τ54/014-015) | Department of Biology, National and Kapodistrian University of Athens, Athens, Greece | Adamantios Sampson |
| MAN15 | Container 20 – Unknown estate TVI-TVIII, TX, TXI, TXIII-TXXIII (MN/UN/TXXVI/001) | Department of Biology, National and Kapodistrian University of Athens, Athens, Greece | Adamantios Sampson |
| MAN16 | Container 20 – Unknown estate TVI-TVIII, TX, TXI, TXIII-TXXIII (Νο.4 - MN/UN/TXXVΙΙI/001) | Department of Biology, National and Kapodistrian University of Athens, Athens, Greece | Adamantios Sampson |
| MAN17 | Container 20 – Unknown estate TVI-TVIII, TX, TXI, TXIII-TXXIII (No.3 - MN/UN/TXXIV/003) | Department of Biology, National and Kapodistrian University of Athens, Athens, Greece | Adamantios Sampson |
| MAN18 | Container 20 – Unknown estate TVI-TVIII, TX, TXI, TXIII-TXXIII (No.1 - MN/UN/TXXIV/001) | Department of Biology, National and Kapodistrian University of Athens, Athens, Greece | Adamantios Sampson |
| MAN19 | Container 20 – Unknown estate TVI-TVIII, TX, TXI, TXIII-TXXIII (No.2 - MN/UN/TXXIV/002) | Department of Biology, National and Kapodistrian University of Athens, Athens, Greece | Adamantios Sampson |
| MAN20 | Container 20 – Unknown estate TVI-TVIII, TX, TXI, TXIII-TXXIII (MN/UN/TXXV/001) | Department of Biology, National and Kapodistrian University of Athens, Athens, Greece | Adamantios Sampson |
| MAN21 | Container 20 – Unknown estate TVI-TVIII, TX, TXI, TXIII-TXXIII (No.1 - Georgiou 87, MN/GE/T87/001) | Department of Biology, National and Kapodistrian University of Athens, Athens, Greece | Adamantios Sampson |
| MAN22 | Container 19 – Unknown estate TI-TV (No.1 - MN/UN/TV/001) | Department of Biology, National and Kapodistrian University of Athens, Athens, Greece | Adamantios Sampson |
| MAN23 | Container 19 – Unknown estate TI-TV (No.6 - MN/UN/TV/006) | Department of Biology, National and Kapodistrian University of Athens, Athens, Greece | Adamantios Sampson |
| MAN24 | Container 19 – Unknown estate TI-TV (No.6 - MN/UN/TV/006) | Department of Biology, National and Kapodistrian University of Athens, Athens, Greece | Adamantios Sampson |
| MAN25 | Container 17A – Georgiou estate T92 (No.2 - MN/GE/T92/004) | Department of Biology, National and Kapodistrian University of Athens, Athens, Greece | Adamantios Sampson |
| MAN26 | Container 17A – Georgiou estate T92 (No.8 - MN/GE/T92/010) | Department of Biology, National and Kapodistrian University of Athens, Athens, Greece | Adamantios Sampson |
| MAN27 | Container 17A – Georgiou estate T92 (No.8 - MN/GE/T92/010) | Department of Biology, National and Kapodistrian University of Athens, Athens, Greece | Adamantios Sampson |
| MAN28 | T.126 | Department of Biology, National and Kapodistrian University of Athens, Athens, Greece | Adamantios Sampson |
| MAN29 | Afendakis estate MN/AF/006 | Department of Biology, National and Kapodistrian University of Athens, Athens, Greece | Adamantios Sampson |
| MAN30 | T.I (T.1) | Department of Biology, National and Kapodistrian University of Athens, Athens, Greece | Adamantios Sampson |
| MAN31 | Belligiani estate – T.129 MN/BE/T129/002 | Department of Biology, National and Kapodistrian University of Athens, Athens, Greece | Adamantios Sampson |
| CA1 | KS4 | Center for GeoGenetics, GLOBE Institute, University of Copenhagen, Copenhagen, Denmark | Morten E. Allentoft |
| CA2 | KS7 | Center for GeoGenetics, GLOBE Institute, University of Copenhagen, Copenhagen, Denmark | Morten E. Allentoft |
| CA3 | KS16 | Center for GeoGenetics, GLOBE Institute, University of Copenhagen, Copenhagen, Denmark | Morten E. Allentoft |
| CA4 | KS20 | Center for GeoGenetics, GLOBE Institute, University of Copenhagen, Copenhagen, Denmark | Morten E. Allentoft |
| CA5 | KS21 | Center for GeoGenetics, GLOBE Institute, University of Copenhagen, Copenhagen, Denmark | Morten E. Allentoft |
| CA6 | KS24 | Center for GeoGenetics, GLOBE Institute, University of Copenhagen, Copenhagen, Denmark | Morten E. Allentoft |
| VEM146 | N/A | Trinity College Dublin, Dublin, Ireland | Mike Parker Pearson; Niall Sharples |
| VEM147 | N/A | Trinity College Dublin, Dublin, Ireland | Mike Parker Pearson; Niall Sharples |
| VEM148 | N/A | Trinity College Dublin, Dublin, Ireland | Mike Parker Pearson; Niall Sharples |
| VEM149 | N/A | Trinity College Dublin, Dublin, Ireland | Mike Parker Pearson; Niall Sharples |
| VEM178 | N/A | Trinity College Dublin, Dublin, Ireland | Mike Parker Pearson; Niall Sharples |
| VEM179 | N/A | Trinity College Dublin, Dublin, Ireland | Mike Parker Pearson; Niall Sharples |
| KAS1 | 9 – 94776397; 52857722A; L121, Sq 6/19, B20116 | Ephorate of antiquities of Phokis, Delphi, Greece | Ioannis Liritzis |
| KAS2 | 9 – 94776397; 52857722A; L121, Sq 6/19, B20116 | Ephorate of antiquities of Phokis, Delphi, Greece | Ioannis Liritzis |
| KAS3 | 9 – 94776397; 52857722A; L121, Sq 6/19, B20116 | Ephorate of antiquities of Phokis, Delphi, Greece | Ioannis Liritzis |
| KAS4 | 11 – 30974738; 12345678; 97483818; L121, Sq 6/19, B20140 | Ephorate of antiquities of Phokis, Delphi, Greece | Ioannis Liritzis |
| KAS5 | 11 – 30974738; 12345678; 97483818; L121, Sq 6/19, B20140 | Ephorate of antiquities of Phokis, Delphi, Greece | Ioannis Liritzis |
| KAS6 | 11 – 30974738; 12345678; 97483818; L121, Sq 6/19, B20140 | Ephorate of antiquities of Phokis, Delphi, Greece | Ioannis Liritzis |
| KAS7 | 11 – 30974738; 12345678; 97483818; L121, Sq 6/19, B20140 | Ephorate of antiquities of Phokis, Delphi, Greece | Ioannis Liritzis |
| KAS8 | 11 – 30974738; 12345678; 97483818; L121, Sq 6/19, B20140 | Ephorate of antiquities of Phokis, Delphi, Greece | Ioannis Liritzis |
| KAS9 | 7 – 48398073; 33640962; 87789391; L121, Sq 6/19, B20073 | Ephorate of antiquities of Phokis, Delphi, Greece | Ioannis Liritzis |
| KAS10 | 1 – 59298376; 87612616; 29077677; L112, B20058 | Ephorate of antiquities of Phokis, Delphi, Greece | Ioannis Liritzis |
| KAS11 | 10 – 34060985; 11111113; 12998749; L121, Sq 6/19, B20160 | Ephorate of antiquities of Phokis, Delphi, Greece | Ioannis Liritzis |
| KAS12 | 1 – 59298376; 65089241; 29077667; L112, B20058 | Ephorate of antiquities of Phokis, Delphi, Greece | Ioannis Liritzis |
| KAS13 | 7 – 48398073; 47609330; 87789391; L121, Sq 6/19, B20073 | Ephorate of antiquities of Phokis, Delphi, Greece | Ioannis Liritzis |
| KAS14 | 10; 11111112; 61112898; L121, Sq 6/19, B20134 | Ephorate of antiquities of Phokis, Delphi, Greece | Ioannis Liritzis |
| KAS15 | 7 – 48398073; 37362902; 84352657; L121, Sq 6/19, B20109 | Ephorate of antiquities of Phokis, Delphi, Greece | Ioannis Liritzis |
| KAS16 | 7 – 48398073; 24499362; 35640748; L112, Sq 6/19, b20089 | Ephorate of antiquities of Phokis, Delphi, Greece | Ioannis Liritzis |
| KAS17 | 10 – 34060985; 11111112; 61112898; L121, Sq 6/19, B20134 | Ephorate of antiquities of Phokis, Delphi, Greece | Ioannis Liritzis |
| KAS18 | 7 – 48398073; 18312153; L120, Sq 6/19, B20108 | Ephorate of antiquities of Phokis, Delphi, Greece | Ioannis Liritzis |
| KAS19 | 9 – 94776397; 16290418; L111, Sq 6/19, B20060 | Ephorate of antiquities of Phokis, Delphi, Greece | Ioannis Liritzis |
| KAS22 | 7 – 48398073; 30713435; L119, Sq 6/19, B20102 | Ephorate of antiquities of Phokis, Delphi, Greece | Ioannis Liritzis |
| KAS23 | 1 – 59298376; 22163458; 72788836; B20001 | Ephorate of antiquities of Phokis, Delphi, Greece | Ioannis Liritzis |
| KAS26 | 10 – 34060985; 11111112; 61112898; L121, Sq 6/19, B20134 | Ephorate of antiquities of Phokis, Delphi, Greece | Ioannis Liritzis |
| KAS28 | 7 – 48398073; 50611878; L110, Sq 5/19, B20063 | Ephorate of antiquities of Phokis, Delphi, Greece | Ioannis Liritzis |
| KAS29 | 7 – 48398073; 18312153; L120, Sq 6/19, B20108 | Ephorate of antiquities of Phokis, Delphi, Greece | Ioannis Liritzis |
| VEM193 | N/A | Trinity College Dublin, Dublin, Ireland | Wiltshire Museum, Devizes, United Kingdom |
| VEM194 | N/A | Trinity College Dublin, Dublin, Ireland | Wiltshire Museum, Devizes, United Kingdom |
| VEM195 | N/A | Trinity College Dublin, Dublin, Ireland | Wiltshire Museum, Devizes, United Kingdom |
| VEM196 | N/A | Trinity College Dublin, Dublin, Ireland | Wiltshire Museum, Devizes, United Kingdom |
| VEM197 | N/A | Trinity College Dublin, Dublin, Ireland | Wiltshire Museum, Devizes, United Kingdom |
| VEM198 | N/A | Trinity College Dublin, Dublin, Ireland | Wiltshire Museum, Devizes, United Kingdom |
| VEM201 | N/A | Trinity College Dublin, Dublin, Ireland | Wiltshire Museum, Devizes, United Kingdom |
| VEM180 | N/A | Trinity College Dublin, Dublin, Ireland | Hampshire Cultural Trust, Winchester, United Kingdom |
| VEM181 | N/A | Trinity College Dublin, Dublin, Ireland | Hampshire Cultural Trust, Winchester, United Kingdom |
| VEM182 | N/A | Trinity College Dublin, Dublin, Ireland | Hampshire Cultural Trust, Winchester, United Kingdom |
| VEM100 | N/A | Trinity College Dublin, Dublin, Ireland | Terry O'Connor; York Archaeological Trust |
| VEM101 | N/A | Trinity College Dublin, Dublin, Ireland | Terry O'Connor; York Archaeological Trust |
| VEM102 | N/A | Trinity College Dublin, Dublin, Ireland | Terry O'Connor; York Archaeological Trust |
| VEM103 | N/A | Trinity College Dublin, Dublin, Ireland | Terry O'Connor; York Archaeological Trust |
| VEM108 | N/A | Trinity College Dublin, Dublin, Ireland | Terry O'Connor; York Archaeological Trust |
| VEM111 | N/A | Trinity College Dublin, Dublin, Ireland | Terry O'Connor; York Archaeological Trust |
| MEC1-4 | GR188-1 | Archaeology department of the city council of Mechelen, Mechelen, Belgium | Katrien Van de Vijver; Bart Robberechts |
| MEC5-9 | 483-1 | Archaeology department of the city council of Mechelen, Mechelen, Belgium | Katrien Van de Vijver; Bart Robberechts |
| MEC10-14 | 27-1 | Archaeology department of the city council of Mechelen, Mechelen, Belgium | Katrien Van de Vijver; Bart Robberechts |
| MEC15-19 | 665-1 | Archaeology department of the city council of Mechelen, Mechelen, Belgium | Katrien Van de Vijver; Bart Robberechts |
| MEC20-24 | 88-1 | Archaeology department of the city council of Mechelen, Mechelen, Belgium | Katrien Van de Vijver; Bart Robberechts |
| MEC25-29 | 246-1 | Archaeology department of the city council of Mechelen, Mechelen, Belgium | Katrien Van de Vijver; Bart Robberechts |
| MEC30-34 | 260-3 | Archaeology department of the city council of Mechelen, Mechelen, Belgium | Katrien Van de Vijver; Bart Robberechts |
| MEC35-39 | 683-1 | Archaeology department of the city council of Mechelen, Mechelen, Belgium | Katrien Van de Vijver; Bart Robberechts |
| MEC40-43 | 435-2 | Archaeology department of the city council of Mechelen, Mechelen, Belgium | Katrien Van de Vijver; Bart Robberechts |
| MEC44-48 | 135-1 | Archaeology department of the city council of Mechelen, Mechelen, Belgium | Katrien Van de Vijver; Bart Robberechts |
| MEC49-53 | 37-1 | Archaeology department of the city council of Mechelen, Mechelen, Belgium | Katrien Van de Vijver; Bart Robberechts |
| MEC54-58 | 303-1 | Archaeology department of the city council of Mechelen, Mechelen, Belgium | Katrien Van de Vijver; Bart Robberechts |
| MEC59-63 | 318-1 | Archaeology department of the city council of Mechelen, Mechelen, Belgium | Katrien Van de Vijver; Bart Robberechts |
| MEC64-68 | 203-6 | Archaeology department of the city council of Mechelen, Mechelen, Belgium | Katrien Van de Vijver; Bart Robberechts |
| MEC69-73 | 597-1 | Archaeology department of the city council of Mechelen, Mechelen, Belgium | Katrien Van de Vijver; Bart Robberechts |
| MEC74-78 | 282-1 | Archaeology department of the city council of Mechelen, Mechelen, Belgium | Katrien Van de Vijver; Bart Robberechts |
| MEC79-83 | 360-1 | Archaeology department of the city council of Mechelen, Mechelen, Belgium | Katrien Van de Vijver; Bart Robberechts |
| MEC84-87 | 358-1 | Archaeology department of the city council of Mechelen, Mechelen, Belgium | Katrien Van de Vijver; Bart Robberechts |
| MEC88-92 | 680-1 | Archaeology department of the city council of Mechelen, Mechelen, Belgium | Katrien Van de Vijver; Bart Robberechts |
| MEC93-97 | 624-1 | Archaeology department of the city council of Mechelen, Mechelen, Belgium | Katrien Van de Vijver; Bart Robberechts |
| MEC98-101 | 422-1 | Archaeology department of the city council of Mechelen, Mechelen, Belgium | Katrien Van de Vijver; Bart Robberechts |
| DEN 1 | NM X681(1) | Holmens Kirke sogn, Sokkelund herred, Holmens Kanal 21, 1060 København K, Denmark | Niels Lynnerup; Morten E. Allentoft |
| DEN 2 | NM X681(3) | Holmens Kirke sogn, Sokkelund herred, Holmens Kanal 21, 1060 København K, Denmark | Niels Lynnerup; Morten E. Allentoft |
| DEN 3 | NM X608(4) | Holmens Kirke sogn, Sokkelund herred, Holmens Kanal 21, 1060 København K, Denmark | Niels Lynnerup; Morten E. Allentoft |
| DEN 4 | NM X608(5) | Holmens Kirke sogn, Sokkelund herred, Holmens Kanal 21, 1060 København K, Denmark | Niels Lynnerup; Morten E. Allentoft |
| DEN 5 | NM Løsfund X3(6) | Holmens Kirke sogn, Sokkelund herred, Holmens Kanal 21, 1060 København K, Denmark | Niels Lynnerup; Morten E. Allentoft |
| DEN 6 | NM Løsfund X4(7) | Holmens Kirke sogn, Sokkelund herred, Holmens Kanal 21, 1060 København K, Denmark | Niels Lynnerup; Morten E. Allentoft |
| DEN 7 | NM Løsfund X2 | Holmens Kirke sogn, Sokkelund herred, Holmens Kanal 21, 1060 København K, Denmark | Niels Lynnerup; Morten E. Allentoft |
| DEN 8 | NM Løsfund X2 | Holmens Kirke sogn, Sokkelund herred, Holmens Kanal 21, 1060 København K, Denmark | Niels Lynnerup; Morten E. Allentoft |
| DEN 9 | NM X nummer(10) | Holmens Kirke sogn, Sokkelund herred, Holmens Kanal 21, 1060 København K, Denmark | Niels Lynnerup; Morten E. Allentoft |
