# Supplementary material for: Screening archaeological bone for palaeogenetic and palaeoproteomic studies
Source: PLoS One. 2020 Jun 25;15(6):e0235146. doi: 10.1371/journal.pone.0235146 (PMC7316274; doi:10.1371/journal.pone.0235146)
Supplement: S3 Table — Alpha Platinum versus Vertex 70v vacuum FTIR-ATR. * Alpha Platinum; ** Vertex 70v. (DOCX) [file pone.0235146.s003.docx]

**S3 Table. Reproducibility and comparability of mid-IR data.** Alpha Platinum versus a Vertex 70v vacuum FTIR-ATR. * Alpha Platinum; ** Vertex 70v

| **Sample** | **IRSF^*^** | **IRSF^**^** | **Difference** | **C/P^*^** | **C/P^**^** | **Difference** | **Am/P^*^** | **Am/P^**^** | **Difference** |
| --- | --- | --- | --- | --- | --- | --- | --- | --- | --- |
| BOV-1 | 3.22 | 3.15 | 0.07 | 0.24 | 0.26 | -0.01 | 0.21 | 0.22 | -0.01 |
| BOV-2 | 3.23 | 3.18 | 0.05 | 0.25 | 0.26 | -0.01 | 0.22 | 0.23 | -0.01 |
| BOV-3 | 3.21 | 3.08 | 0.13 | 0.25 | 0.27 | -0.02 | 0.22 | 0.24 | -0.02 |
| **BOV-Average** | **3.22 ± 0.01** | **3.14 ± 0.05** | **0.08** | **0.25 ± 0.01** | **0.26 ± 0.01** | **-0.02** | **0.22 ±0.01** | **0.23 ± 0.01** | **-0.01** |
| KAS 14-1 | 3.46 | 3.43 | 0.03 | 0.21 | 0.22 | 0.00 | 0.05 | 0.05 | 0.00 |
| KAS 14-2 | 3.23 | 3.49 | -0.26 | 0.26 | 0.23 | 0.03 | 0.06 | 0.05 | 0.02 |
| KAS 14-3 | 3.41 | 3.47 | -0.05 | 0.21 | 0.22 | 0.00 | 0.05 | 0.04 | 0.00 |
| **KAS 14-Average** | **3.37 ± 0.12** | **3.46 ± 0.03** | **-0.10** | **0.23 ± 0.03** | **0.22 ± 0.01** | **0.01** | **0.05 ± 0.01** | **0.04 ± 0.00** | **0.01** |
| SAR 16-1 | 3.60 | 3.50 | 0.09 | 0.21 | 0.23 | -0.02 | 0.03 | 0.04 | 0.00 |
| SAR 16-2 | 3.37 | 3.53 | -0.16 | 0.27 | 0.21 | 0.05 | 0.05 | 0.03 | 0.02 |
| SAR 16-3 | 3.43 | 3.41 | 0.02 | 0.25 | 0.24 | 0.01 | 0.04 | 0.03 | 0.01 |
| **SAR 16-Average** | **3.47 ± 0.12** | **3.48 ± 0.06** | **-0.01** | **0.24 ± 0.03** | **0.23 ± 0.01** | **0.02** | **0.04 ± 0.01** | **0.03 ± 0.00** | **0.01** |
| SAR 19-1 | 3.51 | 3.54 | -0.03 | 0.23 | 0.20 | 0.02 | 0.03 | 0.03 | 0.01 |
| SAR 19-2 | 3.52 | 3.62 | -0.10 | 0.23 | 0.20 | 0.03 | 0.03 | 0.02 | 0.01 |
| SAR 19-3 | 3.63 | 3.59 | 0.04 | 0.20 | 0.21 | -0.01 | 0.03 | 0.03 | 0.00 |
| **SAR 19-Average** | **3.55 ± 0.07** | **3.58 ± 0.04** | **-0.03** | **0.22 ± 0.02** | **0.20 ± 0.01** | **0.01** | **0.03 ± 0.00** | **0.03 ± 0.00** | **0.01** |
| KAS 4-1 | 3.68 | 3.70 | -0.03 | 0.17 | 0.17 | 0.00 | 0.04 | 0.04 | 0.00 |
| KAS 4-2 | 3.70 | 3.66 | 0.04 | 0.17 | 0.17 | 0.00 | 0.04 | 0.03 | 0.01 |
| KAS 4-3 | 3.68 | 3.71 | -0.04 | 0.17 | 0.16 | 0.01 | 0.04 | 0.03 | 0.01 |
| **KAS 4-Average** | **3.69 ± 0.02** | **3.69 ± 0.03** | **-0.01** | **0.17 ± 0.00** | **0.17 ± 0.01** | **0.01** | **0.04 ± 0.00** | **0.03 ± 0.00** | **0.01** |
| MAR 5-1 | 3.70 | 3.89 | -0.19 | 0.24 | 0.19 | 0.05 | 0.01 | 0.00 | 0.01 |
| MAR 5-2 | 3.84 | 3.76 | 0.08 | 0.21 | 0.23 | -0.01 | 0.01 | 0.01 | 0.00 |
| MAR 5-3 | 3.81 | 3.58 | 0.23 | 0.22 | 0.25 | -0.03 | 0.01 | 0.01 | 0.00 |
| **MAR 5-Average** | **3.78 ± 0.07** | **3.74 ± 0.16** | **0.04** | **0.22 ± 0.01** | **0.22 ± 0.03** | **0.00** | **0.01 ± 0.00** | **0.01 ± 0.00** | **0.00** |
| SAR 9-1 | 3.92 | 3.83 | 0.09 | 0.18 | 0.18 | 0.00 | 0.02 | 0.02 | 0.00 |
| SAR 9-2 | 3.97 | 3.74 | 0.23 | 0.17 | 0.18 | -0.01 | 0.02 | 0.02 | 0.00 |
| SAR 9-3 | 3.79 | 3.96 | -0.17 | 0.19 | 0.21 | -0.02 | 0.02 | 0.02 | 0.01 |
| **SAR 9-Average** | **3.89 ± 0.09** | **3.84 ± 0.11** | **0.05** | **0.18 ± 0.01** | **0.19 ± 0.02** | **-0.01** | **0.02 ± 0.00** | **0.02 ± 0.00** | **0.00** |
| KAS 12-1 | 4.18 | 4.07 | 0.10 | 0.19 | 0.20 | -0.02 | 0.04 | 0.04 | 0.00 |
| KAS 12-2 | 4.04 | 4.13 | -0.09 | 0.20 | 0.18 | 0.02 | 0.04 | 0.03 | 0.01 |
| KAS 12-3 | 4.06 | 4.17 | -0.11 | 0.20 | 0.18 | 0.01 | 0.04 | 0.03 | 0.01 |
| **KAS 12-Average** | **4.09 ± 0.08** | **4.12 ± 0.05** | **-0.03** | **0.20 ± 0.01** | **0.19 ± 0.01** | **0.01** | **0.04 ± 0.00** | **0.03 ± 0.00** | **0.00** |
| MEC 61-1 | 4.48 | 4.51 | -0.03 | 0.09 | 0.08 | 0.00 | 0.02 | 0.01 | 0.01 |
| MEC 61-2 | 4.30 | 4.31 | -0.01 | 0.10 | 0.09 | 0.01 | 0.02 | 0.01 | 0.01 |
| MEC 61-3 | 4.32 | 4.37 | -0.04 | 0.09 | 0.10 | -0.01 | 0.02 | 0.02 | 0.00 |
| **MEC 61-Average** | **4.37 ± 0.10** | **4.40 ± 0.10** | **-0.03** | **0.09 ± 0.01** | **0.09 ± 0.01** | **0.00** | **0.02 ± 0.00** | **0.01 ± 0.00** | **0.00** |
| KAS 6-1 | 4.80 | 4.83 | -0.02 | 0.11 | 0.09 | 0.01 | 0.03 | 0.02 | 0.01 |
| KAS 6-2 | 4.78 | 4.90 | -0.12 | 0.10 | 0.10 | 0.01 | 0.02 | 0.02 | 0.01 |
| KAS 6-3 | 4.64 | 4.86 | -0.22 | 0.11 | 0.10 | 0.01 | 0.03 | 0.02 | 0.01 |
| **KAS 6-Average** | **4.74 ± 0.09** | **4.86 ± 0.04** | **-0.12** | **0.11 ± 0.01** | **0.10 ± 0.00** | **0.01** | **0.03 ± 0.00** | **0.02 ± 0.00** | **0.01** |
| MAN 15-1 | 5.10 | 5.03 | 0.07 | 0.10 | 0.12 | -0.01 | 0.01 | 0.01 | 0.00 |
| MAN 15-2 | 5.00 | 5.16 | -0.15 | 0.11 | 0.11 | 0.00 | 0.01 | 0.00 | 0.00 |
| MAN 15-3 | 4.83 | 5.28 | -0.45 | 0.12 | 0.10 | 0.02 | 0.01 | 0.00 | 0.01 |
| **MAN 15-Average** | **4.98 ± 0.13** | **5.15 ± 0.13** | **-0.18** | **0.11 ± 0.01** | **0.11 ± 0.01** | **0.00** | **0.01 ± 0.00** | **0.00 ± 0.00** | **0.00** |
| MAN 29-1 | 5.06 | 5.12 | -0.06 | 0.13 | 0.13 | 0.01 | 0.01 | 0.00 | 0.00 |
| MAN 29-2 | 5.25 | 5.23 | 0.02 | 0.12 | 0.12 | 0.00 | 0.01 | 0.00 | 0.00 |
| MAN 29-3 | 4.98 | 5.10 | -0.12 | 0.14 | 0.13 | 0.01 | 0.01 | 0.01 | 0.00 |
| **MAN 29-Average** | **5.10 ± 0.14** | **5.15 ± 0.07** | **-0.06 ±** | **0.13 ± 0.01** | **0.13 ± 0.01** | **0.00 ±** | **0.01 ± 0.00** | **0.00 ± 0.00** | **0.00 ±** |
| KAS 11-1 | 5.32 | 5.62 | -0.30 | 0.08 | 0.07 | 0.01 | 0.02 | 0.01 | 0.01 |
| KAS 11-2 | 5.33 | 5.73 | -0.41 | 0.08 | 0.06 | 0.01 | 0.02 | 0.01 | 0.01 |
| KAS 11-3 | 5.34 | 5.55 | -0.21 | 0.08 | 0.07 | 0.01 | 0.02 | 0.01 | 0.00 |
| **KAS 11-Average** | **5.33 ± 0.01** | **5.64 ± 0.09** | **-0.31** | **0.08 ± 0.00** | **0.07 ± 0.00** | **0.01** | **0.02 ± 0.00** | **0.01 ± 0.00** | **0.00** |
| MAN 25-1 | 5.31 | 5.59 | -0.28 | 0.09 | 0.09 | 0.00 | 0.01 | 0.01 | 0.00 |
| MAN 25-2 | 5.55 | 5.56 | -0.01 | 0.09 | 0.09 | 0.00 | 0.01 | 0.01 | 0.00 |
| MAN 25-3 | 5.48 | 5.35 | 0.13 | 0.09 | 0.10 | -0.01 | 0.01 | 0.01 | 0.00 |
| **MAN 25-Average** | **5.45 ± 0.12** | **5.50 ± 0.13** | **-0.05** | **0.09 ± 0.00** | **0.10 ± 0.01** | **-0.01** | **0.01 ± 0.00** | **0.01 ± 0.00** | **0.00** |
| MAN 24-1 | 5.71 | 6.22 | -0.51 | 0.08 | 0.08 | 0.01 | 0.01 | 0.00 | 0.00 |
| MAN 24-2 | 5.67 | 5.71 | -0.04 | 0.09 | 0.09 | 0.00 | 0.01 | 0.00 | 0.00 |
| MAN 24-3 | 5.45 | 5.56 | -0.11 | 0.10 | 0.10 | 0.00 | 0.01 | 0.01 | 0.00 |
| **MAN 24-Average** | **5.61 ± 0.14** | **5.83 ± 0.35** | **-0.22** | **0.09 ± 0.01** | **0.09 ± 0.01** | **0.00** | **0.01 ± 0.00** | **0.00 ± 0.00** | **0.00** |
| MAN 32-1 | 5.91 | 5.84 | 0.07 | 0.08 | 0.09 | -0.01 | 0.00 | 0.00 | 0.00 |
| MAN 32-2 | 5.77 | 5.86 | -0.10 | 0.08 | 0.08 | 0.00 | 0.00 | 0.00 | 0.00 |
| MAN 32-3 | 5.83 | 6.08 | -0.25 | 0.08 | 0.08 | 0.00 | 0.00 | 0.00 | 0.00 |
| **MAN 32-Average** | **5.83 ± 0.07** | **5.93 ± 0.13** | **-0.09** | **0.08 ± 0.00** | **0.08 ± 0.00** | **0.00** | **0.00 ± 0.00** | **0.00 ± 0.00** | **0.00** |
| Fluka HAP-1 | 6.23 | 6.21 | 0.02 | N/A | N/A | N/A | N/A | N/A | N/A |
| Fluka HAP-2 | 5.94 | 6.24 | -0.30 | N/A | N/A | N/A | N/A | N/A | N/A |
| Fluka HAP-3 | 6.53 | 6.05 | 0.49 | N/A | N/A | N/A | N/A | N/A | N/A |
| **Fluka HAP-Average** | **6.23 ± 0.30** | **6.17 ± 0.11** | **0.07** | **N/A** | **N/A** | **N/A** | **N/A** | **N/A** | **N/A** |
| **Total variation** |  |  | **-0.06** |  |  | **0.00** |  |  | **0.00** |
