# Supplementary material for: Screening archaeological bone for palaeogenetic and palaeoproteomic studies
Source: PLoS One. 2020 Jun 25;15(6):e0235146. doi: 10.1371/journal.pone.0235146 (PMC7316274; doi:10.1371/journal.pone.0235146)
Supplement: S4 Table — Infrared splitting factor (IRSF), carbonate-to-phosphate (C/P), amide-to-phosphate (Am/P), amide-to-carbonate1 (Am/C1), amide-to-carbonate2 (Am/C2). The + symbol next to samples’ names indicate samples that sampling for DNA analysis preceded. The letter next to endogenous DNA yields denotes the ancient DNA lab the data originate, i.e. C = Copenhagen, D = Dublin, and M = Mainz. N/A: not applicable. (DOCX) [file pone.0235146.s004.docx]

**S4 Table. FTIR, collagen wt. % and endogenous DNA data.** Infrared splitting factor (IRSF), carbonate-to phosphate (C/P), amide-to-phosphate (Am/P), amide-to-carbonate_1_ (Am/C_1_), amide-to-carbonate_2_ (Am/C_2_). The + symbol next to samples’ names indicate samples that sampling for DNA analysis preceded. The letter next to endogenous DNA yields denotes the ancient DNA lab the data originate, i.e. C = Copenhagen, D = Dublin, and M = Mainz. N/A: not applicable.

| **Sample** | **IRSF** | **C/P** | **Am/P** | **Am/C_1_** | **Am/C_2_** | **Collagen wt. %** | **% Endogenous DNA** | **% Endogenous DNA (same pipeline)** | **Total reads sequenced** |
| --- | --- | --- | --- | --- | --- | --- | --- | --- | --- |
| BED1+ | 3.38 ± 0.07 | 0.21 ± 0.01 | 0.06 ± 0.00 | N/A | N/A | N/A | 3.85M | 6.43 | 1,588,827 |
| BED2+ | 3.35 ± 0.04 | 0.21 ± 0.01 | 0.05 ± 0.00 | N/A | N/A | N/A | 0.88M | 1.42 | 790,610 |
| BED3+ | 3.27 ± 0.07 | 0.25 ± 0.02 | 0.06 ± 0.01 | N/A | N/A | N/A | 9.71M | 16.30 | 560,902 |
| BED4+ | 3.31 ± 0.01 | 0.22 ± 0.00 | 0.05 ± 0.00 | N/A | N/A | N/A | 15.53M | 24.29 | 491,776 |
| BED9+ | 3.34 ± 0.03 | 0.23 ± 0.00 | 0.05 ± 0.00 | N/A | N/A | N/A | 1.15M | 1.69 | 199,427 |
| MAR1+ | 3.73 ± 0.14 | 0.24 ± 0.03 | 0.01 ± 0.00 | 0.05 ± 0.00 | 0.08 ± 0.01 | 0.08 | 0.15M | 0.01 | 671,582 |
| MAR2 | 3.77 ± 0.11 | 0.21 ± 0.02 | 0.02 ± 0.00 | 0.07 ± 0.00 | 0.11 ± 0.00 | 0.31 | N/A | N/A | N/A |
| MAR3 | 3.78 ± 0.09 | 0.22 ± 0.02 | 0.01 ± 0.00 | 0.04 ± 0.00 | 0.07 ± 0.00 | 0.38 | N/A | N/A | N/A |
| MAR4 | 3.73 ± 0.08 | 0.24 ± 0.02 | 0.01 ± 0.00 | 0.05 ± 0.00 | 0.09 ± 0.00 | 1.03 | N/A | N/A | N/A |
| MAR5 | 3.78 ± 0.07 | 0.22 ± 0.01 | 0.01 ± 0.00 | 0.05 ± 0.00 | 0.09 ± 0.00 | 0.48 | N/A | N/A | N/A |
| MAR6 | 3.84 ± 0.10 | 0.22 ± 0.02 | 0.01 ± 0.00 | 0.04 ± 0.00 | 0.07 ± 0.00 | 0.23 | N/A | N/A | N/A |
| MAR7 | 3.85 ± 0.16 | 0.27 ± 0.03 | 0.01 ± 0.00 | 0.03 ± 0.00 | 0.07 ± 0.00 | 0.58 | N/A | N/A | N/A |
| MAR8 | 3.80 ± 0.05 | 0.23 ± 0.01 | 0.00 ± 0.00 | 0.00 ± 0.00 | 0.00 ± 0.00 | 0.17 | N/A | N/A | N/A |
| MAR9+ | 4.88 ± 0.26 | 0.12 ± 0.09 | 0.02 ± 0.00 | 0.09 ± 0.00 | 0.12 ±0.00 | 0.63 | 0.12M | 0.01 | 595,808 |
| MAR10+ | 4.95 ± 0.19 | 0.11 ± 0.01 | 0.01 ± 0.00 | 0.12 ± 0.01 | 0.16 ± 0.01 | 0.36 | 0.18M | 0.02 | 505,113 |
| MAR11 | 3.65 ± 0.04 | 0.29 ± 0.01 | 0.00 ± 0.00 | 0.01 ± 0.00 | 0.01 ± 0.00 | 0.00 | N/A | N/A | N/A |
| MAR12 | 3.70 ± 0.06 | 0.29 ± 0.01 | 0.07 ± 0.00 | 0.25 ± 0.00 | 0.36 ± 0.00 | 0.48 | N/A | N/A | N/A |
| MAR13 | 3.67 ± 0.06 | 0.26 ± 0.01 | 0.01 ± 0.00 | 0.05 ± 0.00 | 0.08 ± 0.00 | 0.29 | N/A | N/A | N/A |
| MAR14 | 3.67 ± 0.14 | 0.25 ± 0.03 | 0.05 ± 0.01 | 0.19 ± 0.00 | 0.30 ± 0.01 | 0.32 | N/A | N/A | N/A |
| MAR15 | 3.57 ± 0.10 | 0.28 ± 0.03 | 0.03 ± 0.00 | 0.09 ± 0.00 | 0.15 ± 0.00 | 0.00 | N/A | N/A | N/A |
| MAR16+ | 3.43 ± 0.10 | 0.34 ± 0.03 | 0.02 ± 0.00 | 0.05 ± 0.00 | 0.08 ± 0.00 | 0.78 | 0.41M | 0.01 | 769,459 |
| VEM139+ | 3.43 ± 0.05 | 0.24 ± 0.00 | 0.01 ± 0.00 | N/A | N/A | N/A | 0.07D | 0.42 | 82,919 |
| VEM140+ | 3.37 ± 0.05 | 0.21 ± 0.01 | 0.01 ± 0.00 | N/A | N/A | N/A | 3.10D | 7.14 | 43,129 |
| VEM141+ | 3.49 ± 0.08 | 0.22 ± 0.01 | 0.01 ± 0.00 | N/A | N/A | N/A | 0.06D | 0.69 | 117,035 |
| VEM143+ | 3.46 ± 0.12 | 0.21 ± 0.02 | 0.01 ± 0.00 | N/A | N/A | N/A | 0.08D | 0.58 | 60,105 |
| SAR1 | 3.32 ± 0.06 | 0.31 ± 0.01 | 0.13 ± 0.01 | 0.42 ± 0.01 | 0.82 ± 0.03 | 14.40 | N/A | N/A | N/A |
| SAR2 | 3.43 ± 0.08 | 0.23 ± 0.02 | 0.05 ± 0.00 | 0.21 ± 0.00 | 0.39 ± 0.01 | 9.13 | N/A | N/A | N/A |
| SAR3 | 3.56 ± 0.07 | 0.27 ± 0.02 | 0.06 ± 0.00 | 0.21 ± 0.00 | 0.36 ± 0.00 | 11.07 | N/A | N/A | N/A |
| SAR4 | 3.59 ± 0.07 | 0.21 ± 0.01 | 0.03 ± 0.00 | 0.15 ± 0.00 | 0.27 ± 0.00 | 4.72 | N/A | N/A | N/A |
| SAR5 | 3.33 ± 0.05 | 0.32 ± 0.01 | 0.11 ± 0.00 | 0.34 ± 0.00 | 0.63 ± 0.01 | 19.45 | N/A | N/A | N/A |
| SAR6 | 3.32 ± 0.06 | 0.31 ± 0.02 | 0.11 ± 0.01 | 0.35 ± 0.00 | 0.67 ± 0.01 | 20.51 | N/A | N/A | N/A |
| SAR7 | 3.29 ± 0.08 | 0.30 ± 0.02 | 0.19 ± 0.02 | 0.63 ± 0.02 | 1.31 ± 0.03 | 20.29 | N/A | N/A | N/A |
| SAR8+ | 3.32 ± 0.01 | 0.28 ± 0.01 | 0.13 ± 0.01 | 0.46 ± 0.00 | 0.93 ± 0.01 | 14.95 | 60.60M | 77.63 | 624,588 |
| SAR9 | 3.89 ± 0.09 | 0.18 ± 0.01 | 0.02 ± 0.00 | 0.12 ± 0.00 | 0.20 ± 0.01 | 6.18 | N/A | N/A | N/A |
| SAR10 | 3.13 ± 0.04 | 0.32 ± 0.02 | 0.21 ± 0.01 | 0.64 ± 0.01 | 1.34 ± 0.03 | 22.55 | N/A | N/A | N/A |
| SAR11 | 3.34 ± 0.02 | 0.26 ± 0.01 | 0.14 ± 0.00 | 0.53 ± 0.00 | 1.05 ± 0.01 | 13.06 | N/A | N/A | N/A |
| SAR12 | 3.61 ± 0.08 | 0.29 ± 0.03 | 0.04 ± 0.01 | 0.15 ± 0.00 | 0.23 ± 0.01 | 13.39 | N/A | N/A | N/A |
| SAR13 | 3.41 ± 0.03 | 0.25 ± 0.01 | 0.11 ± 0.00 | 0.44 ± 0.00 | 0.86 ± 0.01 | 16.71 | N/A | N/A | N/A |
| SAR14 | 3.36 ± 0.02 | 0.28 ± 0.00 | 0.17 ± 0.00 | 0.61 ± 0.01 | 1.23 ± 0.02 | 21.13 | N/A | N/A | N/A |
| SAR15 | 3.44 ± 0.02 | 0.25 ± 0.00 | 0.14 ± 0.00 | 0.56 ± 0.001 | 1.13 ± 0.02 | 21.41 | N/A | N/A | N/A |
| SAR16 | 3.47 ± 0.12 | 0.24 ± 0.03 | 0.04 ± 0.01 | 0.17 ± 0.00 | 0.29 ± 0.01 | 14.42 | N/A | N/A | N/A |
| SAR17 | 3.47 ± 0.05 | 0.25 ± 0.01 | 0.07 ± 0.01 | 0.28 ± 0.02 | 0.52 ± 0.04 | 7.16 | N/A | N/A | N/A |
| SAR18 | 3.39 ± 0.01 | 0.25 ± 0.00 | 0.15 ± 0.00 | 0.60 ± 0.01 | 1.21 ± 0.02 | 21.11 | N/A | N/A | N/A |
| SAR19 | 3.55 ± 0.07 | 0.22 ± 0.02 | 0.03 ± 0.00 | 0.30 ± 0.26 | 0.51 ± 0.44 | 8.26 | N/A | N/A | N/A |
| SAR24+ | 3.29 ± 0.00 | 0.31 ± 0.01 | 0.07 ± 0.00 | 0.23 ± 0.00 | 0.42 ± 0.01 | 14.65 | 19.62D | 28.92 | 325,571 |
| SAR28+ | 3.33 ± 0.01 | 0.30 ± 0.00 | 0.06 ± 0.00 | 0.20 ± 0.00 | 0.35 ± 0.01 | 7.38 | 15.55D | 25.27 | 250,761 |
| SAR35+ | 3.40 ± 0.06 | 0.25 ± 0.01 | 0.08 ± 0.01 | 0.32 ± 0.01 | 0.58 ± 0.02 | 10.89 | 33.44D | 49.95 | 364,714 |
| SAR38+ | 3.23 ± 0.01 | 0.33 ± 0.01 | 0.05 ± 0.00 | 0.16 ± 0.01 | 0.28 ± 0.01 | 9.21 | 15.35D | 26.49 | 468,772 |
| SAR40+ | 3.50 ± 0.02 | 0.25 ± 0.01 | 0.09 ± 0.01 | 0.37 ± 0.01 | 0.72 ± 0.03 | 5.28 | 48.85D | 75.61 | 578,080 |
| PRO1+ | 3.20 ± 0.02 | 0.32 ± 0.01 | 0.06 ± 0.00 | 0.19 ± 0.00 | 0.37 ± 0.01 | 7.88 | 18.53D | 34.75 | 50,019 |
| PRO2+ | 3.28 ± 0.01 | 0.26 ± 0.00 | 0.05 ± 0.01 | 0.20 ± 0.00 | 0.36 ± 0.01 | 7.08 | 0.18D | 0.34 | 324,175 |
| PRO8+ | 4.00 ± 0.14 | 0.19 ± 0.01 | 0.01 ± 0.00 | 0.06 ± 0.00 | 0.09 ± 0.00 | 1.46 | 0.06D | 0.11 | 614,738 |
| PRO9 | 3.29 ± 0.05 | 0.29 ± 0.01 | 0.10 ± 0.01 | 0.35 ± 0.01 | 0.68 ± 0.01 | 12.02 | N/A | N/A | N/A |
| THA1+ | 3.71 ± 0.03 | 0.20 ± 0.00 | 0.04 ± 0.00 | 0.20 ± 0.00 | 0.37 ± 0.01 | 5.97 | 3.42M | 4.31 | 587,867 |
| THA2+ | 4.16 ± 0.13 | 0.16 ± 0.02 | 0.00 ± 0.00 | 0.00 ± 0.00 | 0.00 ± 0.00 | 0.84 | 0M | N/A | N/A |
| THA3+ | 3.93 ± 0.15 | 0.18 ± 0.02 | 0.04 ± 0.01 | 0.23 ± 0.02 | 0.43 ± 0.03 | 8.18 | 0M | N/A | N/A |
| THA4 | 3.51 ± 0.10 | 0.25 ± 0.02 | 0.03 ± 0.00 | 0.14 ± 0.01 | 0.25 ± 0.01 | 5.36 | N/A | N/A | N/A |
| THA5 | 3.56 ± 0.17 | 0.24 ± 0.04 | 0.05 ± 0.01 | 0.22 ± 0.02 | 0.41 ± 0.04 | 9.87 | N/A | N/A | N/A |
| THA6 | 3.51 ± 0.04 | 0.24 ± 0.02 | 0.03 ± 0.00 | 0.12 ± 0.00 | 0.22 ± 0.00 | 4.34 | N/A | N/A | N/A |
| THA7 | 3.53 ± 0.09 | 0.26 ± 0.02 | 0.04 ± 0.01 | 0.16 ± 0.00 | 0.30 ± 0.00 | 7.13 | N/A | N/A | N/A |
| THA8+ | 3.57 ± 0.06 | 0.24 ± 0.02 | 0.03 ± 0.00 | 0.12 ± 0.01 | 0.22 ± 0.01 | 1.77 | 0.06M | 0.01 | 2,296,184 |
| THA9+ | 3.66 ± 0.05 | 0.25 ± 0.01 | 0.07 ± 0.00 | 0.29 ± 0.01 | 0.51 ± 0.02 | 12.40 | 4.1M | 5.15 | 1,409,034 |
| THA10 | 3.62 ± 0.08 | 0.22 ± 0.02 | 0.03 ± 0.00 | 0.14 ± 0.00 | 0.26 ± 0.00 | 5.62 | N/A | N/A | N/A |
| THA11+ | 3.63 ± 0.06 | 0.23 ± 0.01 | 0.03 ± 0.00 | 0.14 ± 0.00 | 0.27 ± 0.01 | 4.57 | 0.09M | N/A | N/A |
| VEM202+ | 3.50 ± 0.07 | 0.16 ± 0.01 | 0.05 ± 0.00 | N/A | N/A | N/A | 37.86D | 60.04 | 520,042 |
| VEM203+ | 3.43 ± 0.07 | 0.19 ± 0.02 | 0.06 ± 0.01 | N/A | N/A | N/A | 45.13D | 70.70 | 386,078 |
| VEM204+ | 3.32 ± 0.03 | 0.23 ± 0.01 | 0.07 ± 0.00 | N/A | N/A | N/A | 16.19D | 34.66 | 850,057 |
| VEM205+ | 3.53 ± 0.04 | 0.17 ± 0.01 | 0.04 ± 0.00 | N/A | N/A | N/A | 24.46D | 39.07 | 625,447 |
| VEM206+ | 3.70 ± 0.06 | 0.17 ± 0.01 | 0.04 ± 0.00 | N/A | N/A | N/A | 40.84D | 64.01 | 765,844 |
| VEM207+ | 3.75 ± 0.09 | 0.15 ± 0.02 | 0.05 ± 0.01 | N/A | N/A | N/A | 47.11D | 69.68 | 502,999 |
| VEM208+ | 3.82 ± 0.09 | 0.14 ± 0.01 | 0.04 ± 0.00 | N/A | N/A | N/A | 43.54D | 64.14 | 404,539 |
| MAN1+ | 3.69 ± 0.09 | 0.16 ± 0.02 | 0.05 ± 0.01 | 0.29 ± 0.01 | 0.49 ± 0.02 | 4.95 | 52.29M | 64.04 | 511,487 |
| MAN2 | 4.01 ± 0.07 | 0.15 ± 0.01 | 0.02 ± 0.00 | 0.16 ± 0.00 | 0.25 ± 0.00 | 4.83 | N/A | N/A | N/A |
| MAN3 | 4.32 ± 0.08 | 0.12 ± 0.01 | 0.02 ± 0.00 | 0.19 ± 0.00 | 0.32 ± 0.01 | 8.54 | N/A | N/A | N/A |
| MAN4+ | 3.43 ± 0.01 | 0.23 ± 0.00 | 0.07 ± 0.00 | 0.30 ± 0.00 | 0.55 ± 0.00 | 7.57 | 25.10M | 33.57 | 622,202 |
| MAN5+ | 3.53 ± 0.10 | 0.23 ± 0.02 | 0.04 ± 0.01 | 0.17 ± 0.00 | 0.32 ± 0.01 | 7.06 | 2.77M | 3.35 | 390,690 |
| MAN6+ | 3.57 ± 0.05 | 0.22 ± 0.01 | 0.06 ± 0.00 | 0.26 ± 0.00 | 0.49 ± 0.01 | 7.28 | 1.83M | 2.26 | 468,300 |
| MAN7+ | 3.64 ± 0.03 | 0.21 ± 0.01 | 0.04 ± 0.00 | 0.21 ± 0.00 | 0.39 ± 0.01 | 5.93 | 2.65M | 3.21 | 351,640 |
| MAN8 | 4.75 ± 0.14 | 0.12 ± 0.01 | 0.01 ± 0.00 | 0.06 ± 0.00 | 0.09 ± 0.01 | 0.00 | N/A | N/A | N/A |
| MAN9 | 4.69 ± 0.13 | 0.12 ± 0.01 | 0.01 ± 0.00 | 0.07 ± 0.00 | 0.11 ± 0.00 | 0.00 | N/A | N/A | N/A |
| MAN10 | 4.47 ± 0.24 | 0.14 ± 0.02 | 0.01 ± 0.00 | 0.09 ± 0.04 | 0.11 ± 0.00 | 0.00 | N/A | N/A | N/A |
| MAN11 | 4.67 ± 0.07 | 0.12 ± 0.01 | 0.01 ± 0.00 | 0.06 ± 0.00 | 0.10 ± 0.00 | 0.00 | N/A | N/A | N/A |
| MAN12 | 4.15 ± 0.08 | 0.15 ± 0.01 | 0.01 ± 0.00 | 0.09 ± 0.00 | 0.14 ± 0.00 | 0.72 | N/A | N/A | N/A |
| MAN13 | 5.80 ± 0.11 | 0.07 ± 0.00 | 0.01 ± 0.00 | 0.09 ± 0.00 | 0.12 ± 0.00 | 0.00 | N/A | N/A | N/A |
| MAN14 | 5.12 ± 0.12 | 0.09 ± 0.01 | 0.01 ± 0.00 | 0.08 ± 0.00 | 0.12 ± 0.01 | 0.00 | N/A | N/A | N/A |
| MAN15+ | 4.98 ± 0.13 | 0.11 ± 0.01 | 0.01 ± 0.00 | 0.07 ± 0.00 | 0.10 ± 0.00 | 0.00 | 0.41M | 0.09 | 327,011 |
| MAN16 | 5.05 ± 0.16 | 0.09 ± 0.01 | 0.01 ± 0.00 | 0.08 ± 0.01 | 0.13 ± 0.01 | 0.00 | N/A | N/A | N/A |
| MAN17 | 4.33 ± 0.16 | 0.15 ± 0.01 | 0.01 ± 0.00 | 0.08 ± 0.00 | 0.13 ± 0.01 | 6.98 | N/A | N/A | N/A |
| MAN18 | 5.79 ± 0.28 | 0.08 ± 0.01 | 0.01 ± 0.00 | 0.08 ± 0.00 | 0.11 ± 0.01 | 2.58 | N/A | N/A | N/A |
| MAN19 | 5.91 ± 0.14 | 0.06 ± 0.00 | 0.01 ± 0.00 | 0.10 ± 0.00 | 0.14 ± 0.00 | 1.84 | N/A | N/A | N/A |
| MAN20 | 5.90 ± 0.18 | 0.07 ± 0.01 | 0.01 ± 0.00 | 0.08 ± 0.00 | 0.10 ± 0.00 | 0.40 | N/A | N/A | N/A |
| MAN21+ | 4.95 ± 0.06 | 0.10 ± 0.00 | 0.01 ± 0.00 | 0.09 ± 0.00 | 0.14 ± 0.01 | 0.00 | 0.3M | 0.07 | 295,222 |
| MAN22+ | 5.71 ± 0.06 | 0.08 ± 0.00 | 0.01 ± 0.00 | 0.07 ± 0.00 | 0.08 ± 0.00 | 0.00 | 0.45M | 0.13 | 279,201 |
| MAN23 | 5.60 ± 0.34 | 0.08 ± 0.01 | 0.01 ± 0.00 | 0.08 ± 0.00 | 0.12 ± 0.01 | 1.63 | N/A | N/A | N/A |
| MAN24 | 5.61 ± 0.14 | 0.09 ± 0.01 | 0.01 ± 0.00 | 0.08 ± 0.00 | 0.12 ± 0.00 | 0.00 | N/A | N/A | N/A |
| MAN25+ | 5.45 ± 0.12 | 0.09 ± 0.00 | 0.01 ± 0.00 | 0.08 ± 0.00 | 0.11 ± 0.00 | 0.00 | 0.5M | 0.06 | 410,723 |
| MAN26 | 5.62 ± 0.14 | 0.07 ± 0.01 | 0.01 ± 0.00 | 0.07 ± 0.00 | 0.11 ± 0.00 | 0.00 | N/A | N/A | N/A |
| MAN27 | 4.95 ± 0.12 | 0.11 ± 0.01 | 0.01 ± 0.00 | 0.06 ± 0.00 | 0.09 ± 0.00 | 0.39 | N/A | N/A | N/A |
| MAN28+ | 5.10 ± 0.14 | 0.13 ± 0.01 | 0.01 ± 0.00 | 0.05 ± 0.00 | 0.06 ± 0.00 | 0.00 | 0.13M | 0.01 | 655,820 |
| MAN29+ | 5.75 ± 0.27 | 0.08 ± 0.01 | 0.01 ± 0.00 | 0.08 ± 0.01 | 0.11 ± 0.01 | 0.00 | 0.64M | 0.03 | 589,326 |
| MAN30+ | 5.00 ± 0.37 | 0.09 ± 0.02 | 0.01 ± 0.00 | 0.07 ± 0.00 | 0.11 ± 0.01 | 0.00 | 0.17M | 0.01 | 648,355 |
| MAN31+ | 5.83 ± 0.07 | 0.08 ± 0.00 | 0.00 ± 0.00 | 0.05 ± 0.00 | 0.07 ± 0.00 | 2.10 | 0.36M | 0.02 | 555,297 |
| CA1+ | 4.28 ± 0.08 | 0.10 ± 0.00 | 0.08 ± 0.00 | 0.82 ± 0.02 | 1.57 ± 0.04 | 15.26 | 0.12C | 0.17 | 8,618,593 |
| CA2+ | 3.86 ± 0.02 | 0.14 ± 0.01 | 0.10 ± 0.00 | 0.72 ± 0.00 | 1.34 ± 0.02 | 17.74 | 5.77C | 8.17 | 6,942,194 |
| CA3+ | 3.76 ± 0.02 | 0.19 ± 0.01 | 0.14 ± 0.01 | 0.75 ± 0.01 | 1.48 ± 0.04 | 17.29 | 32.01C | 43.22 | 8,543,920 |
| CA4+ | 4.07 ± 0.12 | 0.14 ± 0.02 | 0.02 ± 0.00 | 0.11 ± 0.00 | 0.19 ± 0.00 | 10.53 | 9.58C | 13.50 | 5,811,241 |
| CA5+ | 4.42 ± 0.15 | 0.07 ± 0.01 | 0.04 ± 0.00 | 0.60 ± 0.01 | 0.89 ± 0.01 | 13.60 | N/A | N/A | N/A |
| CA6+ | 3.82 ± 0.06 | 0.18 ± 0.01 | 0.07 ± 0.01 | 0.41 ± 0.01 | 0.74 ± 0.01 | 13.59 | 0.46C | 0.64 | 9,992,868 |
| VEM210+ | 3.18 ± 0.06 | 0.27 ± 0.03 | 0.08 ± 0.01 | N/A | N/A | N/A | 50.46D | 82.83 | 376,508 |
| VEM146+ | 3.27 ± 0.02 | 0.23 ± 0.00 | 0.06 ± 0.00 | N/A | N/A | N/A | 59.13D | 90.66 | 182,592 |
| VEM147+ | 3.23 ± 0.03 | 0.27 ± 0.02 | 0.07 ± 0.00 | N/A | N/A | N/A | 42.03D | 80.61 | 132,518 |
| VEM148+ | 3.27 ± 0.01 | 0.23 ± 0.00 | 0.05 ± 0.00 | N/A | N/A | N/A | 71.12D | 64.04 | 17,469 |
| VEM149+ | 3.22 ± 0.02 | 0.25 ± 0.00 | 0.06 ± 0.00 | N/A | N/A | N/A | 51.99D | 85.86 | 138,382 |
| VEM178+ | 3.22 ± 0.04 | 0.27 ± 0.01 | 0.07 ± 0.01 | N/A | N/A | N/A | 58.16D | 89.92 | 822,946 |
| VEM179+ | 3.31 ± 0.01 | 0.23 ± 0.00 | 0.06 ± 0.00 | N/A | N/A | N/A | 54.26D | 83.12 | 810,766 |
| KAS1 | 5.42 ± 0.05 | 0.10 ± 0.00 | 0.01 ± 0.00 | 0.11 ± 0.00 | 0.15 ± 0.00 | 2.57 | N/A | N/A | N/A |
| KAS2 | 3.49 ± 0.02 | 0.22 ± 0.01 | 0.04 ± 0.00 | 0.18 ± 0.00 | 0..34 ± 0.00 | 6.68 | N/A | N/A | N/A |
| KAS3 | 4.79 ± 0.11 | 0.16 ± 0.01 | 0.01 ± 0.00 | 0.09 ± 0.01 | 0.11 ± 0.01 | 2.26 | N/A | N/A | N/A |
| KAS4 | 3.69 ± 0.02 | 0.17 ± 0.00 | 0.04 ± 0.00 | 0.23 ± 0.00 | 0.40 ± 0.00 | 7.14 | N/A | N/A | N/A |
| KAS5 | 4.15 ± 0.13 | 0.18 ± 0.01 | 0.02 ± 0.00 | 0.09 ± 0.00 | 0.14 ± 0.01 | 5.48 | N/A | N/A | N/A |
| KAS6 | 4.74 ± 0.09 | 0.11 ± 0.01 | 0.03 ± 0.00 | 0.24 ± 0.00 | 0.38 ± 0.01 | 5.79 | N/A | N/A | N/A |
| KAS7 | 5.47 ± 0.09 | 0.09 ± 0.00 | 0.01 ± 0.00 | 0.13 ± 0.00 | 0.18 ± 0.01 | 0.92 | N/A | N/A | N/A |
| KAS8 | 4.14 ± 0.17 | 0.14 ± 0.02 | 0.05 ± 0.01 | 0.36 ± 0.01 | 0.64 ± 0.02 | 11.29 | N/A | N/A | N/A |
| KAS9 | 4.12 ± 0.11 | 0.13 ± 0.01 | 0.05 ± 0.01 | 0.41 ± 0.01 | 0.68 ± 0.01 | 12.07 | N/A | N/A | N/A |
| KAS10 | 4.25 ± 0.06 | 0.12 ± 0.01 | 0.05 ± 0.00 | 0.42 ± 0.00 | 0.72 ± 0.01 | 9.68 | N/A | N/A | N/A |
| KAS11 | 5.33 ± 0.01 | 0.08 ± 0.00 | 0.02 ± 0.00 | 0.22 ± 0.00 | 0.32 ± 0.01 | 2.86 | N/A | N/A | N/A |
| KAS12 | 4.09 ± 0.08 | 0.20 ± 0.01 | 0.04 ± 0.00 | 0.20 ± 0.00 | 0.30 ± 0.01 | 8.14 | N/A | N/A | N/A |
| KAS13 | 3.80 ± 0.16 | 0.16 ± 0.03 | 0.04 ± 0.01 | 0.25 ± 0.01 | 0.45 ± 0.03 | 11.00 | N/A | N/A | N/A |
| KAS14 | 3.37 ± 0.12 | 0.23 ± 0.03 | 0.05 ± 0.01 | 0.22 ± 0.01 | 0.42 ± 0.03 | 6.59 | N/A | N/A | N/A |
| KAS15 | 5.37 ± 0.35 | 0.11 ± 0.02 | 0.01 ± 0.00 | 0.11 ± 0.01 | 0.13 ± 0.01 | 0.86 | N/A | N/A | N/A |
| KAS16+ | 4.12 ± 0.12 | 0.15 ± 0.01 | 0.04 ± 0.00 | 0.26 ± 0.01 | 0.43 ± 0.01 | 10.47 | 21.22M | 27.71 | 695,213 |
| KAS17+ | 4.07 ± 0.08 | 0.16 ± 0.01 | 0.03 ± 0.00 | 0.18 ± 0.00 | 0.29 ± 0.01 | 10.62 | 28.93M | 38.50 | 493,728 |
| KAS18 | 3.57 ± 0.01 | 0.18 ± 0.00 | 0.06 ± 0.00 | 0.34 ± 0.01 | 0.61 ± 0.02 | 10.89 | N/A | N/A | N/A |
| KAS19 | 3.60 ± 0.04 | 0.18 ± 0.01 | 0.04 ± 0.00 | 0.20 ± 0.00 | 0.35 ± 0.00 | 9.21 | N/A | N/A | N/A |
| KAS22 | 3.52 ± 0.06 | 0.25 ± 0.01 | 0.03 ± 0.00 | 0.13 ± 0.00 | 0.23 ± 0.01 | 5.28 | N/A | N/A | N/A |
| KAS23 | 3.61 ± 0.04 | 0.25 ± 0.01 | 0.04 ± 0.00 | 0.15 ± 0.01 | 0.27 ± 0.02 | 7.70 | N/A | N/A | N/A |
| KAS26 | 4.10 ± 0.24 | 0.16 ± 0.03 | 0.01 ± 0.00 | 0.09 ± 0.00 | 0.14 ± 0.01 | 1.90 | N/A | N/A | N/A |
| KAS28 | 3.51 ± 0.06 | 0.25 ± 0.02 | 0.08 ± 0.01 | 0.31 ± 0.01 | 0.64 ± 0.03 | 13.23 | N/A | N/A | N/A |
| KAS29 | 3.91 ± 0.05 | 0.15 ± 0.01 | 0.06 ± 0.01 | 0.43 ± 0.03 | 0.78 ± 0.07 | 10.23 | N/A | N/A | N/A |
| VEM193+ | 3.20 ± 0.01 | 0.23 ± 0.01 | 0.09 ± 0.00 | N/A | N/A | N/A | 35.95D | 60.35 | 299,980 |
| VEM194+ | 3.24 ± 0.06 | 0.20 ± 0.02 | 0.05 ± 0.01 | N/A | N/A | N/A | 30.67D | 49.01 | 597,754 |
| VEM195+ | 3.19 ± 0.08 | 0.23 ± 0.03 | 0.07 ± 0.01 | N/A | N/A | N/A | 12.33D | 22.17 | 260,284 |
| VEM196+ | 3.18 ± 0.04 | 0.21 ± 0.02 | 0.06 ± 0.01 | N/A | N/A | N/A | 48.01D | 72.80 | 352,692 |
| VEM197+ | 3.32 ± 0.03 | 0.21 ± 0.01 | 0.08 ± 0.01 | N/A | N/A | N/A | 14.37D | 23.68 | 469,699 |
| VEM198+ | 3.25 ± 0.00 | 0.21 ± 0.00 | 0.05 ± 0.00 | N/A | N/A | N/A | 10.32D | 18.10 | 337,334 |
| VEM201+ | 3.28 ± 0.01 | 0.24 ± 0.00 | 0.10 ± 0.00 | N/A | N/A | N/A | 35.83D | 62.78 | 417,593 |
| VEM180+ | 3.15 ± 0.04 | 0.25 ± 0.02 | 0.06 ± 0.01 | N/A | N/A | N/A | 48.20D | 64.98 | 433,732 |
| VEM181+ | 3.28 ± 0.03 | 0.22 ± 0.00 | 0.05 ± 0.00 | N/A | N/A | N/A | 21.40D | 35.03 | 547,151 |
| VEM182+ | 3.41 ± 0.02 | 0.22 ± 0.01 | 0.04 ± 0.00 | N/A | N/A | N/A | 56.20D | 91.27 | 951,348 |
| VEM100+ | 3.11 ± 0.02 | 0.25 ± 0.01 | 0.07 ± 0.00 | N/A | N/A | N/A | 55.00D | 86.14 | 1,379,428 |
| VEM209+ | 3.23 ± 0.04 | 0.29 ± 0.01 | 0.10 ± 0.00 | N/A | N/A | N/A | 55.04D | 79.75 | 344,504 |
| VEM101+ | 3.16 ± 0.03 | 0.26 ± 0.01 | 0.07 ± 0.00 | N/A | N/A | N/A | 33.00D | 64.52 | 1,158,977 |
| VEM108+ | 3.21 ± 0.07 | 0.24 ± 0.01 | 0.07 ± 0.00 | N/A | N/A | N/A | 43.00D | 78.46 | 2,044,232 |
| VEM111+ | 3.09 ± 0.04 | 0.29 ± 0.02 | 0.07 ± 0.01 | N/A | N/A | N/A | 46.00D | 85.25 | 1,044,092 |
| VEM102+ | 3.23 ± 0.04 | 0.21 ± 0.01 | 0.05 ± 0.00 | N/A | N/A | N/A | 41.00D | 69.02 | 998,775 |
| VEM103+ | 3.25 ± 0.05 | 0.22 ± 0.02 | 0.06 ± 0.01 | N/A | N/A | N/A | 25.00D | 43.55 | 1,536,289 |
| MEC1 | 4.34 ± 0.07 | 0.08 ± 0.01 | 0.07 ± 0.00 | 0.93 ± 0.01 | 1.11 ± 0.02 | 14.76 | N/A | N/A | N/A |
| MEC2 | 4.27 ± 0.10 | 0.11 ± 0.01 | 0.02 ± 0.00 | 0.20 ± 0.00 | 0.31 ± 0.00 | 7.62 | N/A | N/A | N/A |
| MEC3 | 4.19 ± 0.10 | 0.12 ± 0.01 | 0.02 ± 0.00 | 0.17 ± 0.00 | 0.26 ± 0.00 | 7.01 | N/A | N/A | N/A |
| MEC4 | 3.88 ± 0.08 | 0.15 ± 0.01 | 0.03 ± 0.00 | 0.20 ± 0.01 | 0.32 ± 0.01 | 4.45 | N/A | N/A | N/A |
| MEC5 | 3.75 ± 0.02 | 0.16 ± 0.00 | 0.04 ± 0.00 | 0.24 ± 0.00 | 0.40 ± 0.00 | 7.18 | N/A | N/A | N/A |
| MEC6 | 3.66 ± 0.06 | 0.18 ± 0.01 | 0.07 ± 0.01 | 0.38 ± 0.01 | 0.67 ± 0.02 | 19.84 | N/A | N/A | N/A |
| MEC7 | 3.68 ± 0.08 | 0.18 ± 0.01 | 0.06 ± 0.00 | 0.35 ± 0.00 | 0.40 ± 0.28 | 19.52 | N/A | N/A | N/A |
| MEC8 | 3.54 ± 0.13 | 0.20 ± 0.03 | 0.13 ± 0.02 | 0.62 ± 0.01 | 1.18 ± 0.05 | 16.27 | N/A | N/A | N/A |
| MEC9 | 3.65 ± 0.05 | 0.19 ± 0.01 | 0.15 ± 0.01 | 0.78 ± 0.01 | 1.51 ± 0.03 | 20.69 | N/A | N/A | N/A |
| MEC10 | 3.87 ± 0.04 | 0.12 ± 0.00 | 0.06 ± 0.00 | 0.52 ± 0.00 | 0.82 ± 0.03 | 12.94 | N/A | N/A | N/A |
| MEC11 | 3.77 ± 0.14 | 0.16 ± 0.03 | 0.09 ± 0.02 | 0.53 ± 0.02 | 0.94 ± 0.05 | 16.52 | N/A | N/A | N/A |
| MEC12 | 3.86 ± 0.06 | 0.15 ± 0.01 | 0.07 ± 0.00 | 0.49 ± 0.00 | 0.82 ± 0.01 | 19.48 | N/A | N/A | N/A |
| MEC13 | 3.99 ± 0.06 | 0.13 ± 0.01 | 0.04 ± 0.00 | 0.32 ± 0.00 | 0.53 ± 0.01 | 14.13 | N/A | N/A | N/A |
| MEC14 | 3.98 ± 0.14 | 0.13 ± 0.02 | 0.04 ± 0.00 | 0.28 ± 0.00 | 0.47 ± 0.00 | 15.93 | N/A | N/A | N/A |
| MEC15 | 3.40 ± 0.06 | 0.21 ± 0.02 | 0.07 ± 0.01 | 0.33 ± 0.01 | 0.61 ± 0.02 | 8.32 | N/A | N/A | N/A |
| MEC16 | 3.68 ± 0.17 | 0.18 ± 0.03 | 0.04 ± 0.01 | 0.21 ± 0.01 | 0.37 ± 0.02 | 12.74 | N/A | N/A | N/A |
| MEC17 | 3.57 ± 0.07 | 0.20 ± 0.01 | 0.05 ± 0.01 | 0.21 ± 0.00 | 0.36 ± 0.01 | 9.83 | N/A | N/A | N/A |
| MEC18 | 3.65 ± 0.12 | 0.19 ± 0.02 | 0.02 ± 0.00 | 0.12 ± 0.01 | 0.21 ± 0.02 | 7.22 | N/A | N/A | N/A |
| MEC19 | 3.44 ± 0.04 | 0.23 ± 0.01 | 0.07 ± 0.00 | 0.29 ± 0.01 | 0.52 ± 0.02 | 15.59 | N/A | N/A | N/A |
| MEC20 | 3.86 ± 0.05 | 0.12 ± 0.01 | 0.09 ± 0.01 | 0.80 ± 0.01 | 1.31 ± 0.04 | 16.25 | N/A | N/A | N/A |
| MEC21 | 3.63 ± 0.04 | 0.20 ± 0.01 | 0.17 ± 0.01 | 0.85 ± 0.01 | 1.70 ± 0.04 | 21.71 | N/A | N/A | N/A |
| MEC22 | 3.58 ± 0.04 | 0.20 ± 0.01 | 0.15 ± 0.01 | 0.73 ± 0.00 | 1.43 ± 0.01 | 21.35 | N/A | N/A | N/A |
| MEC23 | 3.60 ± 0.12 | 0.16 ± 0.02 | 0.11 ± 0.01 | 0.69 ± 0.01 | 1.30 ± 0.02 | 18.44 | N/A | N/A | N/A |
| MEC24 | 3.56 ± 0.01 | 0.18 ± 0.01 | 0.16 ± 0.01 | 0.91 ± 0.00 | 1.73 ± 0.01 | 21.28 | N/A | N/A | N/A |
| MEC25 | 3.98 ± 0.04 | 0.11 ± 0.01 | 0.08 ± 0.01 | 0.77 ± 0.00 | 1.22 ± 0.01 | 11.85 | N/A | N/A | N/A |
| MEC26 | 3.73 ± 0.09 | 0.17 ± 0.01 | 0.04 ± 0.00 | 0.25 ± 0.01 | 0.42 ± 0.01 | 13.46 | N/A | N/A | N/A |
| MEC27 | 3.81 ± 0.02 | 0.16 ± 0.00 | 0.04 ± 0.00 | 0.25 ± 0.00 | 0.41 ± 0.01 | 14.40 | N/A | N/A | N/A |
| MEC28 | 3.83 ± 0.10 | 0.16 ± 0.02 | 0.04 ± 0.01 | 0.25 ± 0.01 | 0.42 ± 0.01 | 9.87 | N/A | N/A | N/A |
| MEC29 | 3.97 ± 0.18 | 0.14 ± 0.02 | 0.03 ± 0.01 | 0.21 ± 0.01 | 0.34 ± 0.01 | 9.64 | N/A | N/A | N/A |
| MEC30 | 4.07 ± 0.09 | 0.09 ± 0.01 | 0.05 ± 0.01 | 0.56 ± 0.01 | 0.83 ± 0.01 | 12.60 | N/A | N/A | N/A |
| MEC31 | 3.88 ± 0.03 | 0.15 ± 0.00 | 0.02 ± 0.00 | 0.15 ± 0.00 | 0.24 ± 0.01 | 8.54 | N/A | N/A | N/A |
| MEC32 | 3.83 ± 0.18 | 0.16 ± 0.02 | 0.02 ± 0.00 | 0.15 ± 0.01 | 0.24 ± 0.01 | 5.89 | N/A | N/A | N/A |
| MEC33 | 3.75 ± 0.14 | 0.18 ± 0.02 | 0.03 ± 0.00 | 0.15 ± 0.00 | 0.24 ± 0.01 | 6.65 | N/A | N/A | N/A |
| MEC34 | 3.90 ± 0.08 | 0.16 ± 0.01 | 0.02 ± 0.00 | 0.13 ± 0.00 | 0.20 ± 0.00 | 6.16 | N/A | N/A | N/A |
| MEC35 | 3.43 ± 0.07 | 0.19 ± 0.01 | 0.06 ± 0.00 | 0.33 ± 0.01 | 0.59 ± 0.02 | 9.38 | N/A | N/A | N/A |
| MEC36 | 3.83 ± 0.04 | 0.14 ± 0.00 | 0.03 ± 0.00 | 0.22 ± 0.02 | 0.36 ± 0.03 | 8.04 | N/A | N/A | N/A |
| MEC37 | 3.81 ± 0.14 | 0.16 ± 0.02 | 0.03 ± 0.00 | 0.20 ± 0.01 | 0.33 ± 0.01 | 9.06 | N/A | N/A | N/A |
| MEC38 | 3.80 ± 0.13 | 0.17 ± 0.02 | 0.03 ± 0.01 | 0.16 ± 0.02 | 0.26 ± 0.04 | 8.04 | N/A | N/A | N/A |
| MEC39 | 3.83 ± 0.12 | 0.18 ± 0.02 | 0.03 ± 0.00 | 0.16 ± 0.01 | 0.25 ± 0.02 | 10.21 | N/A | N/A | N/A |
| MEC40 | 3.37 ± 0.06 | 0.24 ± 0.02 | 0.11 ± 0.01 | 0.48 ± 0.01 | 0.92 ± 0.02 | 16.41 | N/A | N/A | N/A |
| MEC41 | 3.56 ± 0.23 | 0.22 ± 0.06 | 0.07 ± 0.03 | 0.30 ± 0.03 | 0.55 ± 0.10 | 16.78 | N/A | N/A | N/A |
| MEC42 | 3.79 ± 0.22 | 0.16 ± 0.03 | 0.05 ± 0.01 | 0.31 ± 0.01 | 0.55 ± 0.02 | 17.72 | N/A | N/A | N/A |
| MEC43 | 3.58 ± 0.09 | 0.20 ± 0.02 | 0.14 ± 0.01 | 0.71 ± 0.01 | 1.40 ± 0.04 | 17.53 | N/A | N/A | N/A |
| MEC44 | 3.90 ± 0.10 | 0.13 ± 0.01 | 0.06 ± 0.01 | 0.50 ± 0.01 | 0.76 ± 0.04 | 9.27 | N/A | N/A | N/A |
| MEC45 | 4.20 ± 0.12 | 0.11 ± 0.01 | 0.02 ± 0.00 | 0.21 ± 0.01 | 0.32 ± 0.02 | 10.85 | N/A | N/A | N/A |
| MEC46 | 3.90 ± 0.17 | 0.14 ± 0.02 | 0.03 ± 0.00 | 0.21 ± 0.01 | 0.34 ± 0.01 | 11.73 | N/A | N/A | N/A |
| MEC47 | 3.86 ± 0.19 | 0.15 ± 0.03 | 0.04 ± 0.01 | 0.24 ± 0.01 | 0.39 ± 0.04 | 7.21 | N/A | N/A | N/A |
| MEC48 | 3.78 ± 0.14 | 0.16 ± 0.02 | 0.04 ± 0.01 | 0.24 ± 0.01 | 0.38 ± 0.02 | 10.07 | N/A | N/A | N/A |
| MEC49 | 3.67 ± 0.08 | 0.17 ± 0.02 | 0.05 ± 0.01 | 0.32 ± 0.01 | 0.54 ± 0.01 | 11.53 | N/A | N/A | N/A |
| MEC50 | 3.74 ± 0.10 | 0.16 ± 0.02 | 0.05 ± 0.01 | 0.27 ± 0.01 | 0.45 ± 0.02 | 9.91 | N/A | N/A | N/A |
| MEC51 | 3.64 ± 0.01 | 0.18 ± 0.00 | 0.06 ± 0.00 | 0.32 ± 0.00 | 0.56 ± 0.01 | 13.44 | N/A | N/A | N/A |
| MEC52 | 3.62 ± 0.09 | 0.19 ± 0.02 | 0.07 ± 0.01 | 0.37 ± 0.01 | 0.65 ± 0.02 | 18.45 | N/A | N/A | N/A |
| MEC53 | 3.97 ± 0.07 | 0.11 ± 0.01 | 0.12 ± 0.01 | 1.08 ± 0.03 | 1.30 ± 0.02 | 14.93 | N/A | N/A | N/A |
| MEC54 | 4.28 ± 0.11 | 0.10 ± 0.01 | 0.05 ± 0.00 | 0.47 ± 0.00 | 0.76 ± 0.00 | 14.20 | N/A | N/A | N/A |
| MEC55 | 4.39 ± 0.22 | 0.09 ± 0.01 | 0.04 ± 0.01 | 0.45 ± 0.01 | 0.71 ± 0.01 | 15.81 | N/A | N/A | N/A |
| MEC56 | 4.00 ± 0.10 | 0.13 ± 0.01 | 0.03 ± 0.00 | 0.20 ± 0.01 | 0.31 ± 0.01 | 5.51 | N/A | N/A | N/A |
| MEC57 | 4.04 ± 0.20 | 0.14 ± 0.02 | 0.02 ± 0.01 | 0.18 ± 0.01 | 0.28 ± 0.02 | 8.99 | N/A | N/A | N/A |
| MEC58 | 3.99 ± 0.09 | 0.14 ± 0.01 | 0.03 ± 0.00 | 0.21 ± 0.01 | 0.34 ± 0.02 | 5.87 | N/A | N/A | N/A |
| MEC59 | 3.84 ± 0.13 | 0.15 ± 0.02 | 0.07 ± 0.01 | 0.46 ± 0.01 | 0.77 ± 0.03 | 8.87 | N/A | N/A | N/A |
| MEC60 | 4.25 ± 0.15 | 0.11 ± 0.02 | 0.02 ± 0.00 | 0.20 ± 0.00 | 0.31 ± 0.00 | 8.95 | N/A | N/A | N/A |
| MEC61 | 4.37 ± 0.10 | 0.09 ± 0.01 | 0.02 ± 0.00 | 0.21 ± 0.01 | 0.31 ± 0.02 | 4.42 | N/A | N/A | N/A |
| MEC62 | 4.25 ± 0.07 | 0.11 ± 0.01 | 0.03 ± 0.00 | 0.25 ± 0.00 | 0.38 ± 0.01 | 5.23 | N/A | N/A | N/A |
| MEC63 | 4.22 ± 0.23 | 0.11 ± 0.02 | 0.03 ± 0.01 | 0.25 ± 0.01 | 0.35 ± 0.01 | 5.52 | N/A | N/A | N/A |
| MEC64 | 3.76 ± 0.12 | 0.13 ± 0.03 | 0.10 ± 0.02 | 0.75 ± 0.01 | 1.20 ± 0.07 | 13.67 | N/A | N/A | N/A |
| MEC65 | 4.23 ± 0.05 | 0.10 ± 0.00 | 0.08 ± 0.00 | 0.80 ± 0.01 | 1.34 ± 0.05 | 18.79 | N/A | N/A | N/A |
| MEC66 | 4.08 ± 0.18 | 0.11 ± 0.02 | 0.08 ± 0.01 | 0.71 ± 0.01 | 1.19 ± 0.01 | 17.30 | N/A | N/A | N/A |
| MEC67 | 4.30 ± 0.12 | 0.11 ± 0.01 | 0.02 ± 0.00 | 0.21 ± 0.01 | 0.33 ± 0.01 | 4.41 | N/A | N/A | N/A |
| MEC68 | 4.43 ± 0.23 | 0.10 ± 0.02 | 0.03 ± 0.01 | 0.32 ± 0.00 | 0..52 ± 0.02 | 9.73 | N/A | N/A | N/A |
| MEC69 | 3.71 ± 0.05 | 0.16 ± 0.01 | 0.05 ± 0.00 | 0.28 ± 0.01 | 0.47 ± 0.02 | 7.24 | N/A | N/A | N/A |
| MEC70 | 3.94 ± 0.18 | 0.14 ± 0.02 | 0.03 ± 0.00 | 0.20 ± 0.00 | 0.32 ± 0.01 | 10.57 | N/A | N/A | N/A |
| MEC71 | 3.86 ± 0.09 | 0.15 ± 0.01 | 0.04 ± 0.00 | 0.28 ± 0.01 | 0.46 ± 0.00 | 12.72 | N/A | N/A | N/A |
| MEC72 | 3.76 ± 0.09 | 0.16 ± 0.02 | 0.06 ± 0.01 | 0.35 ± 0.01 | 0.58 ± 0.01 | 14.71 | N/A | N/A | N/A |
| MEC73 | 3.98 ± 0.12 | 0.14 ± 0.01 | 0.02 ± 0.00 | 0.17 ± 0.00 | 0.27 ± 0.00 | 8.77 | N/A | N/A | N/A |
| MEC74 | 4.12 ± 0.05 | 0.10 ± 0.01 | 0.06 ± 0.00 | 0.64 ± 0.00 | 1.00 ± 0.01 | 9.91 | N/A | N/A | N/A |
| MEC75 | 3.58 ± 0.12 | 0.21 ± 0.03 | 0.05 ± 0.01 | 0.23 ± 0.01 | 0.38 ± 0.02 | 9.05 | N/A | N/A | N/A |
| MEC76 | 3.88 ± 0.11 | 0.15 ± 0.01 | 0.03 ± 0.00 | 0.19 ± 0.01 | 0.32 ± 0.01 | 6.25 | N/A | N/A | N/A |
| MEC77 | 3.69 ± 0.10 | 0.17 ± 0.02 | 0.06 ± 0.01 | 0.32 ± 0.01 | 0.56 ± 0.01 | 7.50 | N/A | N/A | N/A |
| MEC78 | 3.80 ± 0.13 | 0.16 ± 0.02 | 0.04 ± 0.00 | 0.26 ± 0.00 | 0.44 ± 0.00 | 10.31 | N/A | N/A | N/A |
| MEC79 | 4.01 ± 0.05 | 0.11 ± 0.01 | 0.08 ± 0.01 | 0.75 ± 0.02 | 1.16 ± 0.02 | 15.09 | N/A | N/A | N/A |
| MEC80 | 3.76 ± 0.07 | 0.15 ± 0.02 | 0.12 ± 0.01 | 0.79 ± 0.00 | 1.46 ± 0.06 | 20.93 | N/A | N/A | N/A |
| MEC81 | 3.75 ± 0.01 | 0.14 ± 0.00 | 0.13 ± 0.01 | 0.93 ± 0.02 | 1.74 ± 0.06 | 20.51 | N/A | N/A | N/A |
| MEC82 | 3.97 ± 0.10 | 0.12 ± 0.01 | 0.11 ± 0.01 | 0.95 ± 0.01 | 1.68 ± 0.04 | 20.19 | N/A | N/A | N/A |
| MEC83 | 3.60 ± 0.07 | 0.17 ± 0.02 | 0.14 ± 0.01 | 0.82 ± 0.01 | 1.59 ± 0.04 | 22.24 | N/A | N/A | N/A |
| MEC84 | 3.80 ± 0.09 | 0.17 ± 0.02 | 0.04 ± 0.01 | 0.24 ± 0.01 | 0.40 ± 0.02 | 10.07 | N/A | N/A | N/A |
| MEC85 | 3.75 ± 0.15 | 0.23 ± 0.03 | 0.03 ± 0.01 | 0.12 ± 0.01 | 0.19 ± 0.01 | 4.88 | N/A | N/A | N/A |
| MEC86 | 3.77 ± 0.15 | 0.18 ± 0.02 | 0.02 ± 0.00 | 0.13 ± 0.00 | 0.21 ± 0.00 | 6.69 | N/A | N/A | N/A |
| MEC87 | 3.83 ± 0.13 | 0.18 ± 0.02 | 0.02 ± 0.00 | 0.12 ± 0.01 | 0.19 ± 0.01 | 8.14 | N/A | N/A | N/A |
| MEC88 | 3.58 ± 0.09 | 0.22 ± 0.02 | 0.08 ± 0.01 | 0.37 ± 0.02 | 0.61 ± 0.05 | 1.58 | N/A | N/A | N/A |
| MEC89 | 4.13 ± 0.16 | 0.12 ± 0.02 | 0.03 ± 0.00 | 0.21 ± 0.00 | 0.32 ± 0.01 | 7.10 | N/A | N/A | N/A |
| MEC90 | 3.95 ± 0.04 | 0.14 ± 0.01 | 0.04 ± 0.00 | 0.28 ± 0.01 | 0.46 ± 0.01 | 14.47 | N/A | N/A | N/A |
| MEC91 | 3.75 ± 0.14 | 0.15 ± 0.02 | 0.05 ± 0.01 | 0.31 ± 0.01 | 0.55 ± 0.02 | 16.01 | N/A | N/A | N/A |
| MEC92 | 4.05 ± 0.09 | 0.12 ± 0.01 | 0.03 ± 0.00 | 0.28 ± 0.00 | 0.45 ± 0.01 | 10.75 | N/A | N/A | N/A |
| MEC93 | 3.60 ± 0.04 | 0.17 ± 0.01 | 0.06 ± 0.00 | 0.35 ± 0.01 | 0.59 ± 0.03 | 11.76 | N/A | N/A | N/A |
| MEC94 | 3.75 ± 0.10 | 0.17 ± 0.02 | 0.03 ± 0.00 | 0.18 ± 0.00 | 0.29 ± 0.01 | 5.39 | N/A | N/A | N/A |
| MEC95 | 3.80 ± 0.08 | 0.15 ± 0.01 | 0.03 ± 0.00 | 0.18 ± 0.01 | 0.30 ± 0.01 | 6.06 | N/A | N/A | N/A |
| MEC96 | 3.89 ± 0.10 | 0.15 ± 0.01 | 0.02 ± 0.00 | 0.16 ± 0.01 | 0.27 ± 0.03 | 5.48 | N/A | N/A | N/A |
| MEC97 | 3.53 ± 0.12 | 0.22 ± 0.03 | 0.03 ± 0.01 | 0.15 ± 0.01 | 0.25 ± 0.02 | 4.57 | N/A | N/A | N/A |
| MEC98 | 3.51 ± 0.05 | 0.20 ± 0.01 | 0.08 ± 0.00 | 0.41 ± 0.00 | 0.75 ± 0.01 | 13.70 | N/A | N/A | N/A |
| MEC99 | 3.69 ± 0.17 | 0.19 ± 0.03 | 0.05 ± 0.01 | 0.28 ± 0.01 | 0.49 ± 0.02 | 8.93 | N/A | N/A | N/A |
| MEC100 | 3.51 ± 0.07 | 0.22 ± 0.02 | 0.15 ± 0.02 | 0.68 ± 0.01 | 1.33 ± 0.05 | 19.36 | N/A | N/A | N/A |
| MEC101 | 3.98 ± 0.16 | 0.15 ± 0.02 | 0.02 ± 0.00 | 0.16 ± 0.01 | 0.25 ± 0.01 | 6.38 | N/A | N/A | N/A |
| DEN1+ | 3.33 ± 0.04 | 0.25 ± 0.01 | 0.17 ± 0.00 | 0.65 ± 0.01 | 1.27 ± 0.02 | 14.50 | 54.79C | 57.23 | 12,829,091 |
| DEN2+ | 3.24 ± 0.02 | 0.22 ± 0.00 | 0.10 ± 0.00 | 0.43 ± 0.01 | 0.82 ± 0.01 | 11.19 | 41.62C | 54.99 | 18,285,612 |
| DEN3+ | 3.33 ± 0.01 | 0.26 ± 0.00 | 0.16 ± 0.00 | 0.63 ± 0.01 | 1.22 ± 0.04 | 17.84 | 34.90C | 49.29 | 12,668,469 |
| DEN4+ | 3.97 ± 0.11 | 0.11 ± 0.01 | 0.05 ± 0.01 | 0.50 ± 0.01 | 0.50 ± 0.44 | 10.71 | 6.97C | 9.22 | 2,793,133 |
| DEN5+ | 3.68 ± 0.02 | 0.15 ± 0.00 | 0.10 ± 0.00 | 0.65 ± 0.01 | 1.13 ± 0.01 | 11.33 | 4.74C | 6.55 | 19,245,474 |
| DEN6+ | 3.33 ± 0.02 | 0.22 ± 0.01 | 0.14 ± 0.01 | 0.62 ± 0.01 | 1.21 ± 0.02 | 12.91 | 56.34C | 78.37 | 8,768,821 |
| DEN7+ | 3.38 ± 0.03 | 0.20 ± 0.01 | 0.11 ± 0.01 | 0.55 ± 0.01 | 1.03 ± 0.03 | 15.73 | 43.77C | 60.59 | 12,722,298 |
| DEN8+ | 3.27 ± 0.02 | 0.18 ± 0.00 | 0.08 ± 0.00 | 0.45 ± 0.00 | 0.78 ± 0.00 | 10.80 | 43.22C | 56.73 | 12,026,170 |
| DEN9+ | 3.62 ± 0.10 | 0.14 ± 0.02 | 0.07 ± 0.01 | 0.45 ± 0.01 | 0.72 ± 0.02 | 7.42 | 3.54C | 4.30 | 2,399,751 |
