# Supplementary material for: Screening archaeological bone for palaeogenetic and palaeoproteomic studies
Source: PLoS One. 2020 Jun 25;15(6):e0235146. doi: 10.1371/journal.pone.0235146 (PMC7316274; doi:10.1371/journal.pone.0235146)
Supplement: S1 Fig — Distribution of samples with well- (> 10%; green/solid diamond), moderately- (1–10%; yellow/large checkerboard), and poorly-preserved (< 1%; red/dotted) endogenous DNA in categories based on crystallinity (n = 85). Endogenous DNA % were estimated using the same bioinformatics pipeline (see section 2—supporting information for details) for all samples to eliminate the potential effects of the different bioinformatics protocols followed by the three different labs on the estimated yields. THA2, THA3, and THA11 samples were not reprocessed, thus excluded from this graph. The c. 90% of the well-preserved specimens with endogenous DNA > 10% (n = 45) display IRSF values < 3.7, and only c. 10% (n = 7) have 3.7 ≤ IRSF ≤ 4.2. The samples that yield endogenous DNA below 1% predominantly display IRSF values over 3.7 (c. 70%; n = 14), with a small subset (c. 30%; n = 6) characterized by crystallinity below the 3.7 threshold. All samples with IRSF > 4.2 have endogenous DNA yields below 1%. Success rates are similar to those reported in the text, suggesting that this screening method is not affected by the bioinformatics pipeline. (DOCX) [file pone.0235146.s005.docx]

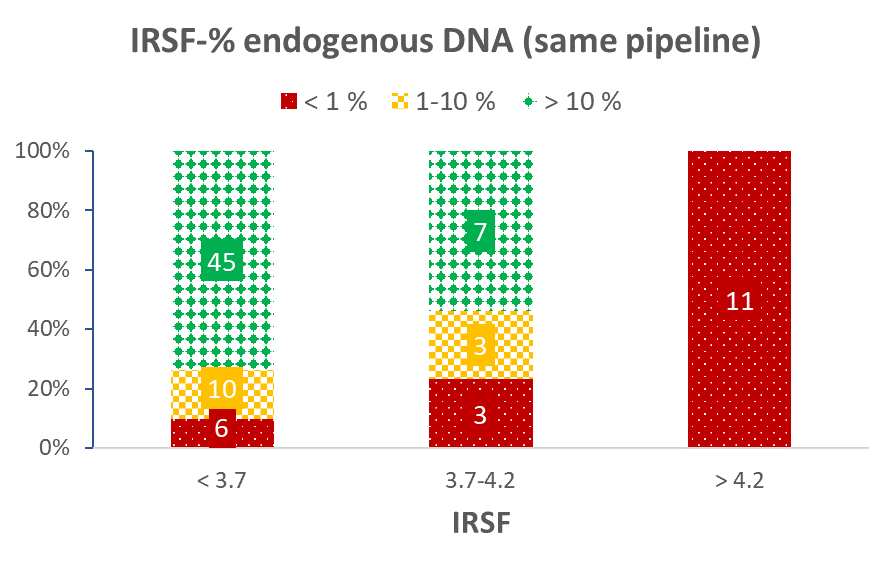


**S1 Figure**. **Endogenous DNA-crystallinity relationship**. Distribution of samples with well- (> 10 %; green/solid diamond), moderately- (1-10 %; yellow/large checkerboard), and poorly-preserved (< 1 %; red/dotted) endogenous DNA in categories based on crystallinity (n = 85). Endogenous DNA % were estimated using the same bioinformatics pipeline (see section 2 - supporting information for details) for all samples to eliminate the potential effects of the different bioinformatics protocols followed by the three different labs on the estimated yields. THA2, THA3, and THA11 samples were not reprocessed, thus excluded from this graph.

The c. 90 % of the well-preserved specimens with endogenous DNA > 10 % (n = 45) display IRSF values < 3.7, and only c. 10 % (n = 7) have 3.7 ≤ IRSF ≤ 4.2. The samples that yield endogenous DNA below 1 % predominantly display IRSF values over 3.7 (c. 70 %; n = 14), with a small subset (c. 30 %; n = 6) characterized by crystallinity below the 3.7 threshold. All samples with IRSF > 4.2 have endogenous DNA yields below 1 %. Success rates are similar to those reported in the text, suggesting that this screening method is not affected by the bioinformatics pipeline.
