# Supplementary material for: Screening archaeological bone for palaeogenetic and palaeoproteomic studies
Source: PLoS One. 2020 Jun 25;15(6):e0235146. doi: 10.1371/journal.pone.0235146 (PMC7316274; doi:10.1371/journal.pone.0235146)
Supplement: S2 Fig — Distribution of samples with well- (> 10%; green/solid diamond), moderately- (1–10%; yellow/large checkerboard), and poorly-preserved (< 1%; red/dotted) endogenous DNA in categories based on carbonate content (n = 85). Endogenous DNA % were also estimated using the same bioinformatics pipeline (see section 2—supporting information for details) for all samples to eliminate the potential effects of the different bioinformatics protocols followed by the three different labs on the estimated yields. THA2, THA3, and THA11 samples were not reprocessed, thus excluded from this graph. The c. 85% of the specimens with endogenous DNA > 1% (n = 64) display C/P values > 0.13, and only c. 15% (n = 11) have endogenous DNA yields below 1%. When C/P drops below 0.13, samples with endogenous DNA yields < 1% prevail (i.e. c. 90%; n = 9), and only c. 10% (n = 1) of samples contain more than 1% endogenous DNA. Success rates are similar to those reported in the text, suggesting that this screening method is not affected by the different bioinformatics pipeline. (DOCX) [file pone.0235146.s006.docx]

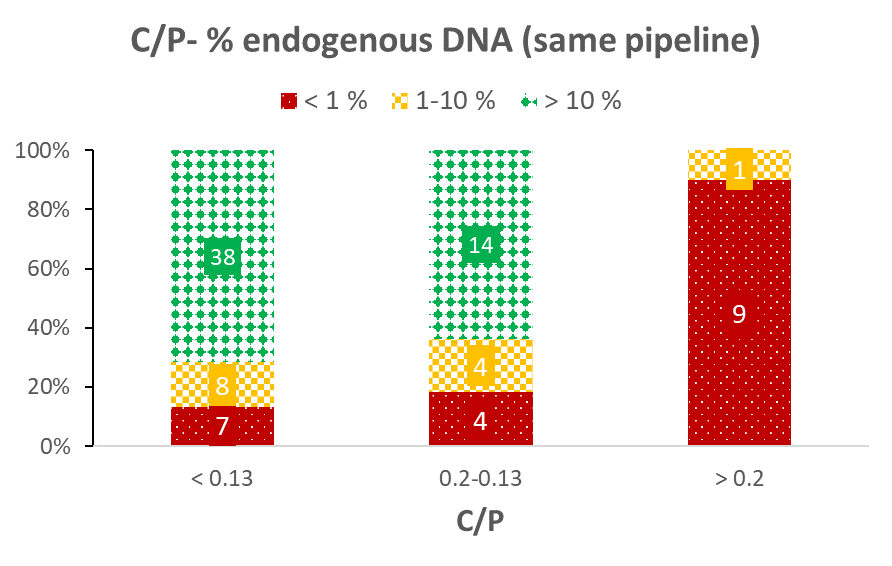


**S2 Figure**. **Endogenous DNA-carbonate content relationship**. Distribution of samples with well- (> 10 %; green/solid diamond), moderately- (1-10 %; yellow/large checkerboard), and poorly-preserved (< 1 %; red/dotted) endogenous DNA in categories based on carbonate content (n = 85). Endogenous DNA % were also estimated using the same bioinformatics pipeline (see section 2 - supporting information for details) for all samples to eliminate the potential effects of the different bioinformatics protocols followed by the three different labs on the estimated yields. THA2, THA3, and THA11 samples were not reprocessed, thus excluded from this graph.

The c. 85 % of the specimens with endogenous DNA > 1 % (n = 64) display C/P values > 0.13, and only c. 15 % (n = 11) have endogenous DNA yields below 1 %. When C/P drops below 0.13, samples with endogenous DNA yields < 1 % prevail (i.e. c. 90 %; n = 9), and only c. 10 % (n = 1) of samples contain more than 1 % endogenous DNA. Success rates are similar to those reported in the text, suggesting that this screening method is not affected by the different bioinformatics pipeline.
