# Supplementary material for: Screening archaeological bone for palaeogenetic and palaeoproteomic studies
Source: PLoS One. 2020 Jun 25;15(6):e0235146. doi: 10.1371/journal.pone.0235146 (PMC7316274; doi:10.1371/journal.pone.0235146)
Supplement: S3 Fig — (a) Collagen preservation shows strong polynomial correlation with Am/C1 (R2 = 0.73; polynomial order = 2). The red line represents the proposed Am/C1 = 0.1 cut-off point. The circles represent the petrous bones and rhombuses the other skeletal elements. (b) Distribution of samples with well- (≥ 3%; green/solid diamond), and poorly-preserved (< 3%; red/dotted) collagen in categories based on Am/C1. (DOCX) [file pone.0235146.s007.docx]

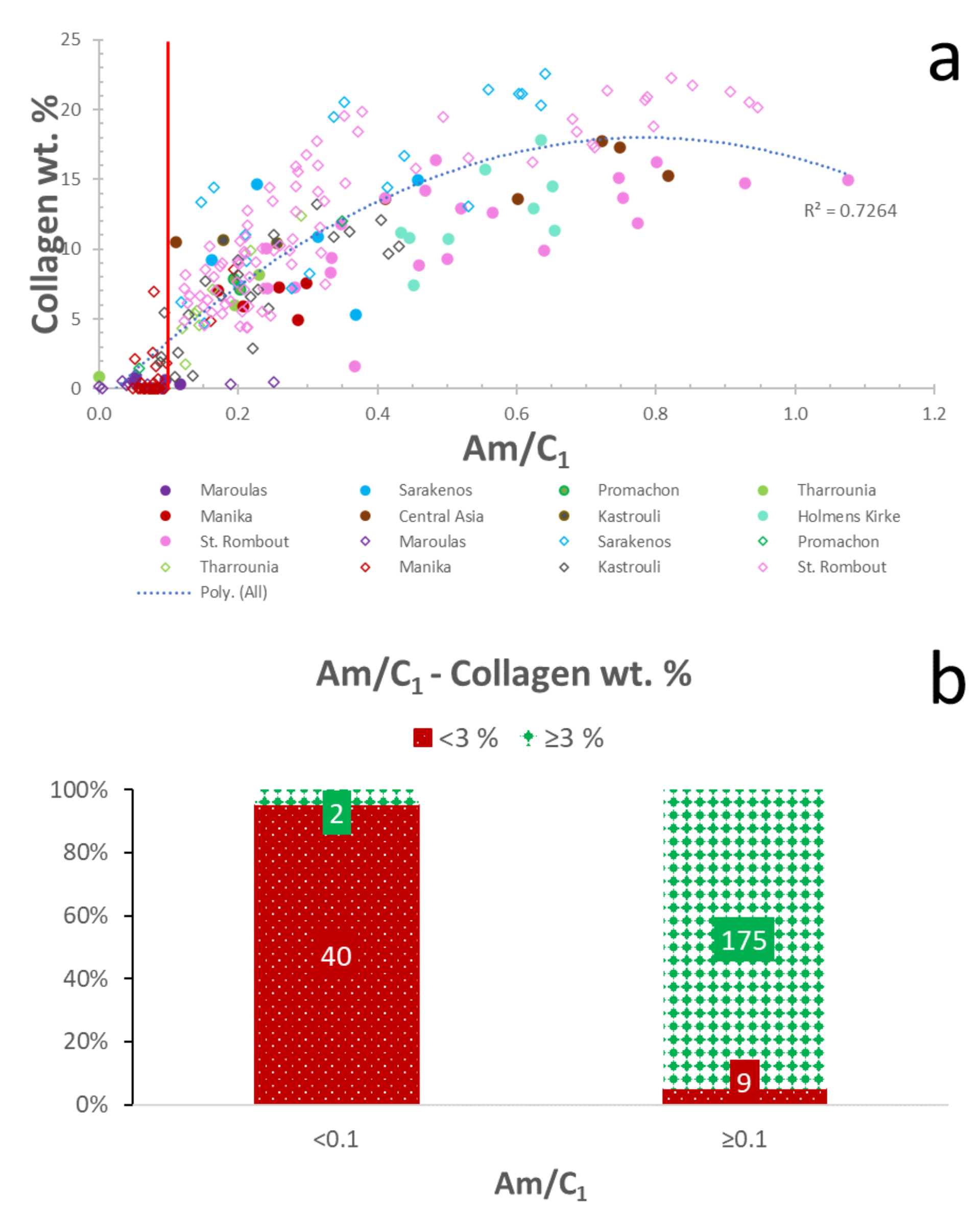


**S3 Figure. Am/C_1_-collagen content relationship**. (a) Collagen preservation shows strong polynomial correlation with Am/C_1_ (R^2^ = 0.73; polynomial order = 2). The red line represents the proposed Am/C_1_ = 0.1 cut-off point. The circles represent the petrous bones and rhombuses the other skeletal elements. (b) Distribution of samples with well- (≥ 3 %; green/solid diamond), and poorly-preserved (< 3 %; red/dotted) collagen in categories based on Am/C_1_.
