# Supplementary material for: Screening archaeological bone for palaeogenetic and palaeoproteomic studies
Source: PLoS One. 2020 Jun 25;15(6):e0235146. doi: 10.1371/journal.pone.0235146 (PMC7316274; doi:10.1371/journal.pone.0235146)
Supplement: S4 Fig — (a) Collagen preservation shows strong polynomial correlation with Am/C2 (R2 = 0.72; polynomial order = 2). The red line represents the proposed Am/C2 = 0.2 cut-off point. The circles represent the petrous bones and rhombuses the other skeletal elements. (b) Distribution of samples with well- (≥ 3%; green/solid diamond), and poorly-preserved (< 3%; red/dotted) collagen in categories based on Am/C2. (DOCX) [file pone.0235146.s008.docx]

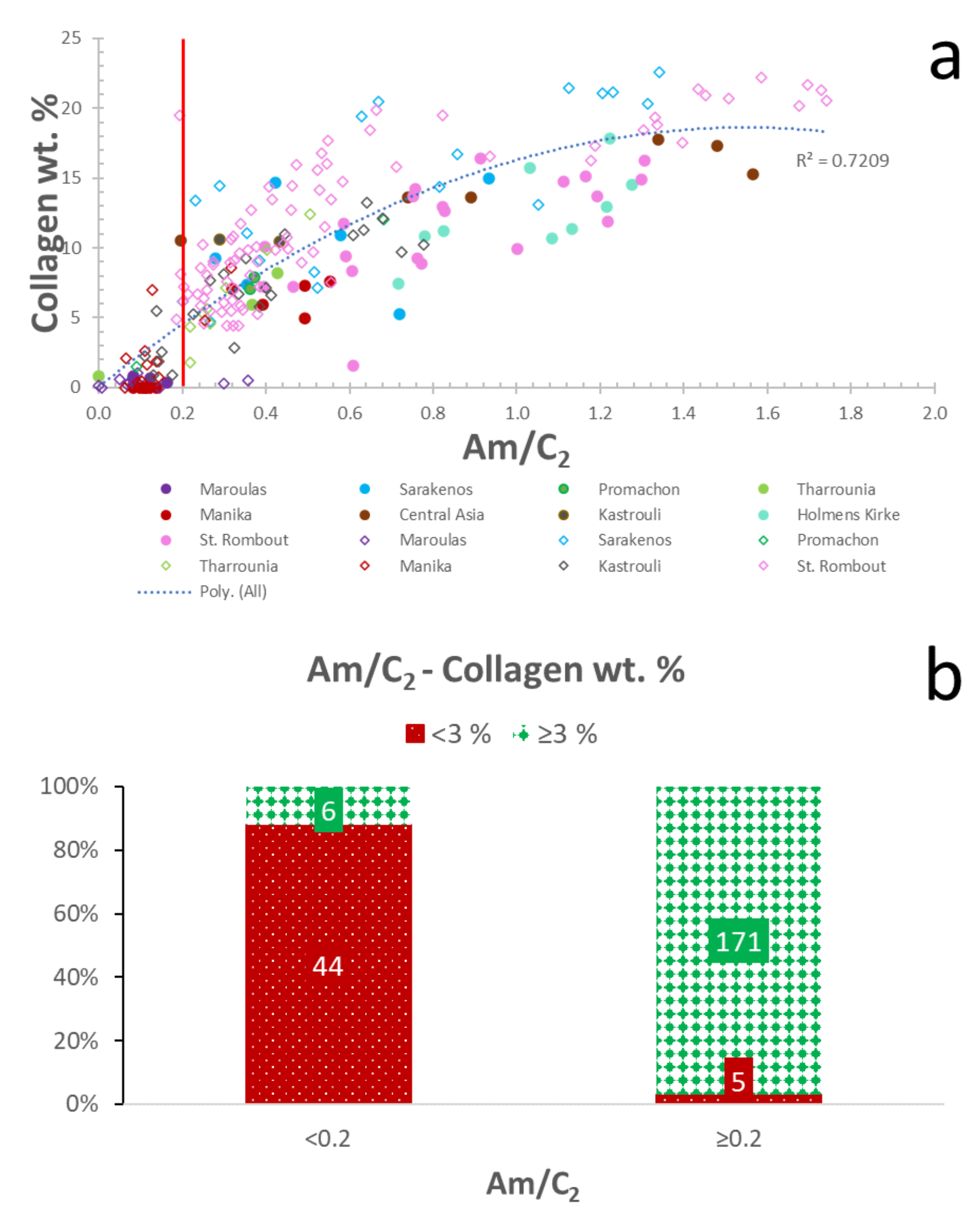


**S4 Figure**. **Am/C_2_-collagen content relationship**. (a) Collagen preservation shows strong polynomial correlation with Am/C_2_ (R^2^ = 0.72; polynomial order = 2). The red line represents the proposed Am/C_2_ = 0.2 cut-off point. The circles represent the petrous bones and rhombuses the other skeletal elements. (b) Distribution of samples with well- (≥ 3 %; green/solid diamond), and poorly-preserved (< 3 %; red/dotted) collagen in categories based on Am/C_2_.
