# Supplementary material for: Screening archaeological bone for palaeogenetic and palaeoproteomic studies
Source: PLoS One. 2020 Jun 25;15(6):e0235146. doi: 10.1371/journal.pone.0235146 (PMC7316274; doi:10.1371/journal.pone.0235146)
Supplement: S1 Text — (DOCX) [file pone.0235146.s009.docx]

## Section 2. Ancient DNA Methods

### Trinity College Dublin

##### Sample Preparation

All sample preparation, extraction and library preparation were performed in a dedicated Ancient DNA laboratory in the Smurfit Institute of Genetics, Trinity College Dublin. Standard aDNA protocols were used throughout the process [(Cooper and Poinar, 2000; Pääbo *et al.*, 2004)](https://paperpile.com/c/tBHMOY/DtUtx+DCsOu). Samples were decontaminated via UV exposure, the removal of surface layers using a dental drill and further UV exposure. Bones were drilled and then powdered with the use of a mixer mill (MM 400, Retsch).

##### DNA Extraction

Extractions of between 0.09 g and 0.3 g of bone powder were performed as described by [(Verdugo *et al.*, 2019)](https://paperpile.com/c/tBHMOY/xw5Kh), based upon [(Yang *et al.*, 1998; MacHugh *et al.*, 2000)](https://paperpile.com/c/tBHMOY/GMrcT+Xfv09). Briefly, the bone powder was incubated at 37^o^C using an Eppendorf ThermoMixer® at 700rpm with 1ml of lysis buffer (Tris HCl pH 7.4 - 20 mM; Proteinase K, recombinant, PCR Grade – 0.65 U/ml; Sarkosyl® NL 30 – 0.7 %; EDTA pH 8 – 47.5 mM) for 24 hours. Samples were then centrifuged for 10 minutes at 10,000 rpm, supernatant removed, fresh lysis buffer added and the incubation and centrifugation steps were repeated. For some samples this was repeated for a third time. As contamination is more likely to be accessed during the first lysis stage [(Vilstrup *et al.*, 2013)](https://paperpile.com/c/tBHMOY/xnIvJ) supernatant from either the second or third lysis stage was taken to the next stage. A Tris-EDTA wash was performed by transferring 1 mL of supernatant to Amicon® Ultra 4 mL filter (Merck Millipore) and adding 3 mL of 1X Tris-EDTA. Each sample was then centrifuged at 2500 rpm until the final volume was reduced to 100ul. The final step of DNA purification was carried out using the “QIAQuick minElute purification kit” (Qiagen) and eluted in 40 -60 µL of EB buffer + Tween® 20 (Sigma-Aldrich). Extraction controls were always included and sequenced for contamination control.

##### Library Preparation

Double stranded libraries were constructed from 16.25ul - 30ul of DNA extract based on the protocol of [(Meyer and Kircher, 2010)](https://paperpile.com/c/tBHMOY/GFJb) with modifications as reported in [(Gamba *et al.*, 2014)](https://paperpile.com/c/tBHMOY/7GlaI). Briefly, blunt end repair was performed using NEBNext® End Repair Module (New England BioLabs Inc.) and during the final step of adapter fill in *Bst* activity was arrested through heat inactivation (20 min at 80°C). Indexing PCRs were performed using Accuprime™ Pfx Supermix (Life Technology), primer IS4 (0.2 μM) and indexing primers(0.2 μM) [(Meyer and Kircher, 2010)](https://paperpile.com/c/tBHMOY/GFJb). 3μl of library were added to a total volume of 25μl of PCR mix and amplified using 5 min at 95°C, 10-12 cycles of 15 sec at 95°C, 30 sec at 60°C and 30 sec at 68°C followed by a final extension of 5 min at 68°C (Gamba et al 2014). PCR products were purified using MinElute MinElute PCR Purification Kit, QIAGEN. Samples VEM180-182 were USER treated prior to blunt end repair as per Verdugo and colleagues 2019.

Assessment of amplified libraries was achieved via either the Agilent 2100 Bioanalyzer or the Agilent 2200 TapeStation, following manufacturer's instructions. Libraries were screened on an Illumina MiSeqTM platform at TrinSeq (Trinity Genome Sequencing Laboratory, Trinity College Dublin, Ireland), using 50 bp single-end sequencing and a PhiX control at 1%.

### University of Copenhagen

##### Sample Preparation

The petrous bones were sampled by cutting off the apex part, slice by slice, until the otic capsule was reached. The samples (fragment of the otic capsule) were crushed and pulverized.

##### DNA Extraction

Bone powder was digested using a buffer containing (per ml) 929 μl EDTA, 10 μl TE buffer, 10 μl proteinase K, 50 μl N-laurylsacrosine and 1 μl phenolred. A volume of 2 ml was used for a 15-minute pre-digestion [1] followed by a full 24h-digestion at 37C in 4 ml digestion buffer. The DNA was extracted using a silica-in-solution extraction method [(Rohland and Hofreiter, 2007)](https://paperpile.com/c/tBHMOY/uqxD), using the binding buffer from Allentoft et al. [(2015)](https://paperpile.com/c/tBHMOY/kpb4/?noauthor=1), which is highly efficient in binding short DNA fragments.

##### Library Preparation

Blunt-ended double-stranded libraries were built using the NEBNext DNA Prep Master Mix Set (New England Biolabs Inc.) with modifications outlined previously [(Orlando *et al.*, 2013; Allentoft *et al.*, 2015)](https://paperpile.com/c/tBHMOY/kpb4+6E1w). The libraries were amplified with indexed Illumina-specific adapters, prepared as in Meyer and Kircher [(Meyer and Kircher, 2010)](https://paperpile.com/c/tBHMOY/GFJb). The DNA concentrations of the amplified libraries were measured on a Bioanalyzer before the libraries were pooled equimolarly and sequenced (100 bp, single read) on an Illumina HiSeq 2500 platform.

### Johannes Gutenberg-University Mainz

##### Sample Preparation

Sample preparation, DNA extraction and library preparation for all samples were conducted in dedicated ancient DNA facilities of the Palaeogenetics Group at Johannes Gutenberg-University Mainz.

The animal samples (Bed*) were processed as described for sample CTC in Botigué et al. [(2017)](https://paperpile.com/c/tBHMOY/wzJq/?noauthor=1) with slight modification as described below. The inner core of the petrous bones was separated from the outer bone layers by stepwise milling and removing of bone powder. However, sample Bed4 was too soft for stepwise milling and completely powderised already at the first milling step. From the inner layer of samples Bed 1-4 (or mixed layers in case of Bed4), 0,3g of bone powder was used to extract the DNA.

For the human long bones, sample preparation, DNA extraction and library preparation followed the protocol described in Hofmanová et al. [(2016)](https://paperpile.com/c/tBHMOY/WMoR/?noauthor=1).

The human petrous bones were prepared as described in Hofmanová et al. [(2016)](https://paperpile.com/c/tBHMOY/WMoR/?noauthor=1).

##### DNA Extraction

For the animal samples, extraction was performed as described for sample CTC in Botigué et al. [(2017)](https://paperpile.com/c/tBHMOY/wzJq/?noauthor=1) with slight modification as described below. The extraction set-up included a washing step (pre-lysis) prior to full bone lysis. For this initial step, the bone powder was incubated for 1 hour at 37°C with 2ml of EDTA (0.5 M, pH8; Ambion®, Applied Biosystems), 250 µl of N-Laurylsarcosine (0.5 %, Merck) and 30 µl of Proteinase K (18 U, Roche) under regular slow vortexing every 10 minutes. The main lysis was performed using 12 ml of EDTA, 250 µl of N-Laurylsarcosine and 30 µl of Proteinase K. For initial washing, 1g of bone powder from sample Bed9 was incubated with 2ml of EDTA at room temperature in a rocking shaker. The supernatant was removed after short centrifugation, and the step was repeated once. The remaining bone powder underwent full lysis using 10 ml of EDTA, 250µl N-Laurylsarcosine and 30µl of Proteinase K.

For the human petrous bone samples, DNA extraction followed the protocol by Yang et al. [(1998)](https://paperpile.com/c/tBHMOY/GMrcT/?noauthor=1) with the modification described in MacHugh et al. [(2000)](https://paperpile.com/c/tBHMOY/Xfv09/?noauthor=1) and Gamba et al. [(2014)](https://paperpile.com/c/tBHMOY/7GlaI/?noauthor=1) as well as additional modifications described below. For each sample, 0.15 g of bone powder were used for extraction. Prior to extraction a pre-lysis was performed by adding 1 mL of EDTA (0.5 M, pH8, Ambion/Applied Biosystems, Life technologies, Darmstadt, Germany) to the bone powder and incubating at room temperature for 10 to 30 minutes. The solution was centrifuged at maximum speed to pellet the powder and the supernatant was removed. Lysis was performed on rocking shakers at 37°C for 24 hours (900 rpm) using 1 ml of extraction buffer containing EDTA (950 μl, 0.5 M, pH8, Ambion/Applied Biosystems, Life technologies, Darmstadt, Germany), Tris-HCl (20 μl, 1 M, pH8, Life Technologies, Carlsbad, United States), N-Laurylsarcosine (17 µl, 5%, Merck Millipore, Darmstadt, Germany) and Proteinase K (13 µl; 20 mg/ml, Roche, Mannheim, Germany). After 24 hours, the samples were centrifuged for 10 minutes at 10,000 rpm, the supernatant was removed and stored in a fridge until further processing. A second lysis step was performed following the same procedure. Following lysis, the supernatants from the lysis steps were either treated separately or merged. The extracts were washed on Amicon Filters (Amicon Ultra-4 30 kDA, 15 ml, Merck Millipore, Darmstadt, Germany) by twice adding 3ml 1X Tris-EDTA, followed by centrifugation at 2500 rpm for 20 minutes. After washing, the sample was concentrated to 100 µl. Samples were subsequently purified with the QIAgen MinElute kit (Qiagen, Venlo, Netherlands) following the manufacturer’s instructions but incubating for 5 minutes during elution with 44 µl elution buffer (preheated to 65°C).

##### Library Preparation

The following protocol was used for the human petrous bone samples: libraries were prepared according to the protocol by Kircher et al. [(2012)](https://paperpile.com/c/tBHMOY/wXoB/?noauthor=1) with slight modifications. Blunt End repair was performed using the NEBNext End Repair Module (New England Biolabs, Ipswich, Massachusetts, United States): 20 µl of DNA extract are mixed with NEBNext End Repair Reaction Buffer (10X, 7 µl), NEBNext End Repair Enzyme Mix (3.5 µl) and nuclease-free water (39.5 µl; for a final reaction volume of 70 µl) and incubated for 15 minutes at 25°C followed by 5 minutes at 12°C. In the adapter ligation step hybridized adapters P5 and P7 (IDT, Leuven, Belgium) were used at a concentration of 0.75 µM. 3 µl of Fill-In product (total volume: 40 µl) were amplified using AccuPrime^TM^ Pfx SuperMix (20 µl; Thermo Fisher Scientific, Waltham, Massachusetts, United States) in one PCR parallel (final reaction volume: 25 µl; final primer concentration: 200 nM each). Double indexing followed Kircher et al. [(2012)](https://paperpile.com/c/tBHMOY/wXoB/?noauthor=1), but using index sequences from the NexteraXT index Kit v2 (Illumina). For each sample the PCR was performed in 12 cycles; the PCR temperature profile followed the manufacturer’s recommendations but using an annealing temperature of 60°C for 30 seconds and performing a final elongation step for 5 minutes.

For purification during library preparation the MinElute PCR Purification Kit (Qiagen, Hilden, Germany) was used, while amplified libraries were purified with the MSB® Spin PCRapace (Invitek, Stratec Molecular, Berlin, Germany). Libraries were quantified by Qubit® Fluorometric quantitation (dsDNA HS assay, Invitrogen) and measurement on the Agilent 2100 Bioanalyzer System (High Sensitivity DNA, Agilent Technologies).

Miseq screening (50 bp single end sequencing) of all samples was performed at StarSEQ GmbH (Mainz, Germany).

### Bioinformatic Pipeline - All Samples

Cutadapt v.1.11 [(Martin, 2011)](https://paperpile.com/c/tBHMOY/vbyBg) was implemented to remove adapter sequences from the sequencing reads, with a final minimum read length of 30 base employed, allowing for a one base overlap between the adapter and the read. Sequence reads were aligned using Burrows-Wheeler Algorithm (BWA) (version 0.7.5) [(Li and Durbin, 2009)](https://paperpile.com/c/tBHMOY/XN1HK) with the seed disabled [(Schubert *et al.*, 2012)](https://paperpile.com/c/tBHMOY/2hH1) to the appropriate reference genome; *Bos taurus* genome, build ARS-UCD1.2; *Ovis aries*, build Oar_rambouillet_v1.0; *Capra hircus*, build ARS1; *Homo sapiens*, build hg19 including rCRS. Duplicates were removed using Picard (version 2.20.3) and reads with a mapping quality inferior to 25 removed by SAMtools (version 1.9) [(Li *et al.*, 2009)](https://paperpile.com/c/tBHMOY/zf88P).

Target species endogenous DNA content was determined as the number of mapped reads divided by the number of reads retained post trimming. While the library sequencing efficiency was determined as the number of target species reads post duplication removal divided by the total number of reads before trimming [(Hansen *et al.*, 2017)](https://paperpile.com/c/tBHMOY/PkM20). For completion, the library efficiency post mapping quality filtering was also calculated by dividing the number of reads aligned post mapping quality filtering by the total number of reads.

Ancient DNA carries signatures of post-mortem deamination, characterised by an increase of cytosine to thymine misincorporation clustered toward the read termini [(Brotherton *et al.*, 2007; Ginolhac *et al.*, 2011)](https://paperpile.com/c/tBHMOY/sDHkq+NDdE3). This chemical signature of aDNA can be utilised to assess the degree of deamination present and therefore judge the authenticity of the aDNA. MapDamage (2.0) [(Jónsson *et al.*, 2013)](https://paperpile.com/c/tBHMOY/XchJB) was performed to assess the patterns of miscorporation in the samples.

### References

[Allentoft, M. E. *et al.* (2015) ‘Population genomics of Bronze Age Eurasia’, *Nature*, 522(7555), pp. 167–172. doi:](http://paperpile.com/b/tBHMOY/kpb4) [10.1038/nature14507](http://dx.doi.org/10.1038/nature14507)[.](http://paperpile.com/b/tBHMOY/kpb4)

[Botigué, L. R. *et al.* (2017) ‘Ancient European dog genomes reveal continuity since the Early Neolithic’, *Nature communications*, 8, p. 16082. doi:](http://paperpile.com/b/tBHMOY/wzJq) [10.1038/ncomms16082](http://dx.doi.org/10.1038/ncomms16082)[.](http://paperpile.com/b/tBHMOY/wzJq)

[Brotherton, P. *et al.* (2007) ‘Novel high-resolution characterization of ancient DNA reveals C> U-type base modification events as the sole cause of post mortem miscoding lesions’, *Nucleic acids research*. Oxford University Press, 35(17), pp. 5717–5728. Available at:](http://paperpile.com/b/tBHMOY/sDHkq) <https://academic.oup.com/nar/article-abstract/35/17/5717/2402212>[.](http://paperpile.com/b/tBHMOY/sDHkq)

[Cooper, A. and Poinar, H. N. (2000) ‘Ancient DNA: do it right or not at all’, *Science*. faculty.ksu.edu.sa, 289(5482), pp. 1139–1139. Available at:](http://paperpile.com/b/tBHMOY/DtUtx) <http://faculty.ksu.edu.sa/archaeology/Publications/General/Ancient%20DNA.doc>[.](http://paperpile.com/b/tBHMOY/DtUtx)

[Gamba, C. *et al.* (2014) ‘Genome flux and stasis in a five millennium transect of European prehistory’, *Nature communications*, 5, p. 5257. doi:](http://paperpile.com/b/tBHMOY/7GlaI) [10.1038/ncomms6257](http://dx.doi.org/10.1038/ncomms6257)[.](http://paperpile.com/b/tBHMOY/7GlaI)

[Ginolhac, A. *et al.* (2011) ‘mapDamage: testing for damage patterns in ancient DNA sequences’, *Bioinformatics* . Oxford Univ Press, 27(15), pp. 2153–2155. doi:](http://paperpile.com/b/tBHMOY/NDdE3) [10.1093/bioinformatics/btr347](http://dx.doi.org/10.1093/bioinformatics/btr347)[.](http://paperpile.com/b/tBHMOY/NDdE3)

[Hansen, H. B. *et al.* (2017) ‘Comparing Ancient DNA Preservation in Petrous Bone and Tooth Cementum’, *PloS one*, 12(1), p. e0170940. doi:](http://paperpile.com/b/tBHMOY/PkM20) [10.1371/journal.pone.0170940](http://dx.doi.org/10.1371/journal.pone.0170940)[.](http://paperpile.com/b/tBHMOY/PkM20)

[Hofmanová, Z. *et al.* (2016) ‘Early farmers from across Europe directly descended from Neolithic Aegeans’, *Proceedings of the National Academy of Sciences of the United States of America*, 113(25), pp. 6886–6891. doi:](http://paperpile.com/b/tBHMOY/WMoR) [10.1073/pnas.1523951113](http://dx.doi.org/10.1073/pnas.1523951113)[.](http://paperpile.com/b/tBHMOY/WMoR)

[Jónsson, H. *et al.* (2013) ‘mapDamage2.0: fast approximate Bayesian estimates of ancient DNA damage parameters’, *Bioinformatics* . Oxford Univ Press, 29(13), pp. 1682–1684. doi:](http://paperpile.com/b/tBHMOY/XchJB) [10.1093/bioinformatics/btt193](http://dx.doi.org/10.1093/bioinformatics/btt193)[.](http://paperpile.com/b/tBHMOY/XchJB)

[Kircher, M., Sawyer, S. and Meyer, M. (2012) ‘Double indexing overcomes inaccuracies in multiplex sequencing on the Illumina platform’, *Nucleic acids research*, 40(1), p. e3. doi:](http://paperpile.com/b/tBHMOY/wXoB) [10.1093/nar/gkr771](http://dx.doi.org/10.1093/nar/gkr771)[.](http://paperpile.com/b/tBHMOY/wXoB)

[Li, H. *et al.* (2009) ‘The Sequence Alignment/Map format and SAMtools’, *Bioinformatics* , 25(16), pp. 2078–2079. doi:](http://paperpile.com/b/tBHMOY/zf88P) [10.1093/bioinformatics/btp352](http://dx.doi.org/10.1093/bioinformatics/btp352)[.](http://paperpile.com/b/tBHMOY/zf88P)

[Li, H. and Durbin, R. (2009) ‘Fast and accurate short read alignment with Burrows–Wheeler transform’, *Bioinformatics* . Oxford University Press, 25(14), pp. 1754–1760. doi:](http://paperpile.com/b/tBHMOY/XN1HK) [10.1093/bioinformatics/btp324](http://dx.doi.org/10.1093/bioinformatics/btp324)[.](http://paperpile.com/b/tBHMOY/XN1HK)

[MacHugh, D. E. *et al.* (2000) ‘The extraction and analysis of ancient DNA from bone and teeth: a survey of current methodologies’, *Ancient biomolecules*. Taylor & Francis, 3, pp. 81–102. Available at:](http://paperpile.com/b/tBHMOY/Xfv09) <http://eprints.hud.ac.uk/id/eprint/25228/> [(Accessed: 30 January 2018).](http://paperpile.com/b/tBHMOY/Xfv09)

[Martin, M. (2011) ‘Cutadapt removes adapter sequences from high-throughput sequencing reads’, *EMBnet.journal*. journal.embnet.org, 17(1), pp. 10–12. doi:](http://paperpile.com/b/tBHMOY/vbyBg) [10.14806/ej.17.1.200](http://dx.doi.org/10.14806/ej.17.1.200)[.](http://paperpile.com/b/tBHMOY/vbyBg)

[Meyer, M. and Kircher, M. (2010) ‘Illumina sequencing library preparation for highly multiplexed target capture and sequencing’, *Cold Spring Harbor protocols*, 2010(6), p. db.prot5448. doi:](http://paperpile.com/b/tBHMOY/GFJb) [10.1101/pdb.prot5448](http://dx.doi.org/10.1101/pdb.prot5448)[.](http://paperpile.com/b/tBHMOY/GFJb)

[Orlando, L. *et al.* (2013) ‘Recalibrating Equus evolution using the genome sequence of an early Middle Pleistocene horse’, *Nature*, 499(7456), pp. 74–78. doi:](http://paperpile.com/b/tBHMOY/6E1w) [10.1038/nature12323](http://dx.doi.org/10.1038/nature12323)[.](http://paperpile.com/b/tBHMOY/6E1w)

[Pääbo, S. *et al.* (2004) ‘Genetic analyses from ancient DNA’, *Annual review of genetics*. annualreviews.org, 38, pp. 645–679. doi:](http://paperpile.com/b/tBHMOY/DCsOu) [10.1146/annurev.genet.37.110801.143214](http://dx.doi.org/10.1146/annurev.genet.37.110801.143214)[.](http://paperpile.com/b/tBHMOY/DCsOu)

[Rohland, N. and Hofreiter, M. (2007) ‘Comparison and optimization of ancient DNA extraction’, *BioTechniques*, 42(3), pp. 343–352. doi:](http://paperpile.com/b/tBHMOY/uqxD) [10.2144/000112383](http://dx.doi.org/10.2144/000112383)[.](http://paperpile.com/b/tBHMOY/uqxD)

[Schubert, M. *et al.* (2012) ‘Improving ancient DNA read mapping against modern reference genomes’, *BMC genomics*, 13, p. 178. doi:](http://paperpile.com/b/tBHMOY/2hH1) [10.1186/1471-2164-13-178](http://dx.doi.org/10.1186/1471-2164-13-178)[.](http://paperpile.com/b/tBHMOY/2hH1)

[Verdugo, M. P. *et al.* (2019) ‘Ancient cattle genomics, origins, and rapid turnover in the Fertile Crescent’, *Science*. American Association for the Advancement of Science, 365(6449), pp. 173–176. doi:](http://paperpile.com/b/tBHMOY/xw5Kh) [10.1126/science.aav1002](http://dx.doi.org/10.1126/science.aav1002)[.](http://paperpile.com/b/tBHMOY/xw5Kh)

[Vilstrup, J. T. *et al.* (2013) ‘Mitochondrial phylogenomics of modern and ancient equids’, *PloS one*, 8(2), p. e55950. doi:](http://paperpile.com/b/tBHMOY/xnIvJ) [10.1371/journal.pone.0055950](http://dx.doi.org/10.1371/journal.pone.0055950)[.](http://paperpile.com/b/tBHMOY/xnIvJ)

[Yang, D. Y. *et al.* (1998) ‘Improved DNA extraction from ancient bones using silica-based spin columns’, *American Journal of Physical Anthropology: The Official Publication of the American Association of Physical Anthropologists*. Wiley Online Library, 105(4), pp. 539–543. Available at:](http://paperpile.com/b/tBHMOY/GMrcT) <https://onlinelibrary.wiley.com/doi/abs/10.1002/(SICI)1096-8644(199804)105:4%3C539::AID-AJPA10%3E3.0.CO;2-1>[.](http://paperpile.com/b/tBHMOY/GMrcT)
